# Effect of the Synthetic Method on the Properties of Ni-Based Hydrogen Oxidation Catalysts

**DOI:** 10.1021/acsaem.0c03157

**Published:** 2021-04-01

**Authors:** Elena S. Davydova, Maidhily Manikandan, Dario R. Dekel, Svein Sunde

**Affiliations:** ^†^The Wolfson Department of Chemical Engineering and ^§^The Nancy & Stephen Grand Technion Energy Program (GTEP), Technion—Israel Institute of Technology, Haifa 3200003, Israel; ‡ Department of Materials Science and Engineering, Norwegian University of Science and Technology (NTNU), NO-7491 Trondheim, Norway

**Keywords:** alkaline anion-exchange membrane, fuel cell, solvothermal reduction, chemical reduction, Raman

## Abstract

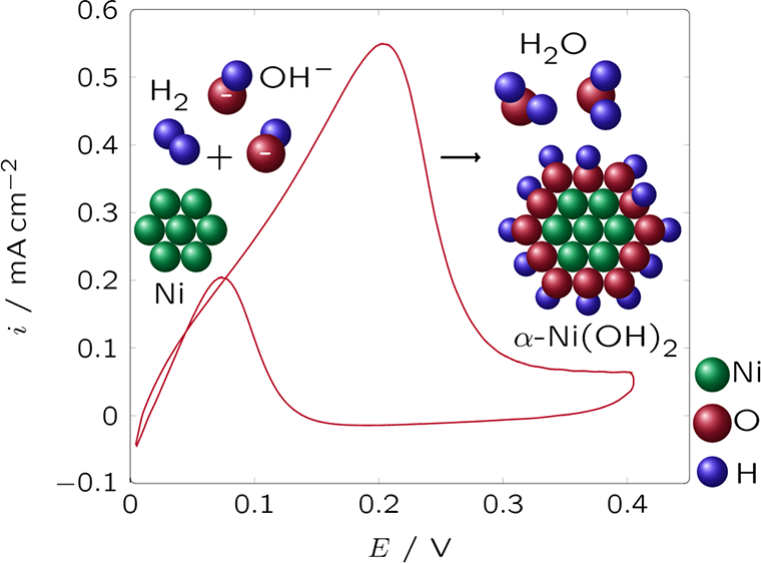

The latest progress in alkaline anion-exchange membranes has led to the expectation that less costly catalysts than those of the platinum-group metals may be used in anion-exchange membrane fuel cell devices. In this work, we compare structural properties and the catalytic activity for the hydrogen-oxidation reaction (HOR) for carbon-supported nanoparticles of Ni, Ni_3_Co, Ni_3_Cu, and Ni_3_Fe, synthesized by chemical and solvothermal reduction of metal precursors. The catalysts are well dispersed on the carbon support, with particle diameter in the order of 10 nm, and covered by a layer of oxides and hydroxides. The activity for the HOR was assessed by voltammetry in hydrogen-saturated aqueous solutions of 0.1 mol dm^–1^ KOH. A substantial activation by potential cycling of the pristine catalysts synthesized by solvothermal reduction is necessary before these become active for the HOR; in situ Raman spectroscopy shows that after activation the surface of the Ni/C, Ni_3_Fe, and Ni_3_Co catalysts is fully reduced at 0 V, whereas the surface of the Ni_3_Cu catalyst is not. The activation procedure had a smaller but negative impact on the catalysts synthesized by chemical reduction. After activation, the exchange-current densities normalized with respect to the ECSA (electrochemically active surface area) were approximately independent of composition but relatively high compared to catalysts of larger particle diameter.

## Introduction

Recently, alkaline anion-exchange membrane fuel cells (AEMFCs) have undergone a rapid progression.^1−3^ This progress is the result of the development of anion-exchange membranes (AEMs) with higher OH^–^ ion conductivity and stability,^4,5^ as well as the growth in the understanding of the AEMFC water management.^6^ The progress is also significantly driven by the advancement in oxygen reduction^7,8^ and hydrogen oxidation electrocatalysts^9−12^ for alkaline media. Until now, mainly platinum group metals (PGMs), namely Pt, PtRu, and Pd-based^13−15^ catalysts, are tested in fuel cells at relatively high loading levels, up to 0.6 mg cm^–2^, on either or both electrodes of AEMFCs.^1,11^ The use of alkaline AEMs opens up possibilities beyond PGMs, significantly expanding the range of non-noble electrode materials, which demonstrate prominent stability in base conditions. Moreover, the presence of hydroxide ions leverages the kinetics of O_2_ reduction on non-noble catalysts via slightly improving their catalytic activity and hindering the reaction for the PGM-based catalysts.^16,17^ In order to benefit from the alkalinity as the main advantage of AEMFCs and, consequently, to enjoy potentially lower costs due to the use of abundant electrode materials, reliable (i.e., active, selective, tolerant to the impurities, and stable) PGM-free electrocatalysts must be developed. This would allow keeping pace with the progress in the AEM development and meeting the future demands of AEMFCs. In the coming years, the PGM-free anodes may become the bottleneck of the AEMFC technology development, whereas the non-noble ORR electrocatalysts are quite advanced^7,8,18^ and already available commercially. Until now, Ni-based materials remain the only alternative to PGMs toward hydrogen electrooxidation, though they are still far from being competitive with Pt.^15,15,19−21,21−24^

The early period of Ni-based electrocatalysts for the hydrogen-oxidation reaction (HOR) was associated with Raney Ni, doped with transition metals^25^ and with the characteristic particle sizes of a few micrometers. The main drawbacks of Raney Ni lie in the unpredictable deactivation during the aging and the difficulty in handling due to the necessity of keeping pyrophoric solids wet or passivated. In fuel cells with anion-exchange membranes, the use of large catalyst particles is not acceptable. Also, the lack of liquid electrolyte to “refresh” the particle surface negatively affects catalyst performance.

Therefore, new synthetic approaches are being implemented for the development of the next generation of Ni-based HOR electrocatalysts for AEMFCs. Among them are electrochemical,^21,26^ physical vapor deposition (PVD),^27,28^ thermochemical,^15,21,23^ and hydrothermal methods,^29^ as well as chemical reduction.^19^ Floner et al.^30^ investigated the effect of Ni surface texture on the HOR catalysis using polycrystalline bulk Ni and Ni facets (100), (110), and (111). They showed that on the oxygen-free surfaces the Ni(110) facet has the highest catalytic activity. However, anodic oxidation of (111), (110), and (100) facets resulted in disordered, polycrystalline surfaces.^30^ Tang et al.^27^ deployed the PVD method to formulate a series of the bulk Ni–Ag (Ni_3_Ag, NiAg, NiAg_3_) catalyst layers. This did, however, result in an undesirable segregation of the metals and the formation of bulk Ni–Ag layers, giving a relatively low HOR activity.^27^ In contrast to these Ni–Ag systems, the use of combinatorial magnetron cosputtering allowed Wang et al.^28^ to reach high degrees of alloying in bulk Ni_*x*_Cu_*y*_ binary catalysts, the optimum Cu content being 40%. On the other hand, Cherstiouk et al.^23^ and Oshchepkov et al.^22^ found that in nanosized carbon-supported Ni–Cu/C catalysts synthesized via thermal reduction of Ni(OAc)_2_ and Cu(OAc)_2_ in H_2_ at 250 °C the optimum catalytic activity for the HOR was reached at a significantly lower amount of Cu (<5%). Also, carbon-supported catalysts with high Ni:metal ratios, Ni_9.5_Cu_0.5_/C^2^ and Ni_9_Mo_1_/C,^15^ have been obtained via thermochemical reduction of simple inorganic precursors at 550 °C in H_2_. The use of these catalysts in AEMFC anodes resulted in the relatively high beginning-of-life power density values of 350 mW cm_geom_^–2^ at 80 °C^2^ and 120 mW cm^–2^_geom_ at 70 °C,^15^ respectively. Sheng et al.^26^ proposed electrochemical deposition of a series of the bulk binary Ni–Mo, Ni–Co and ternary Co_*x*_Ni_*y*_Mo_*z*_ catalysts (*x* = 0–1.05, *y* = 4.17–5.22, *z* = 1) resulting in thick (ca. 0.6 μm) but highly active catalytic layers. The results of Oshchepkov et al.^22^ indicate that for monometallic, carbon-supported Ni catalysts, electrochemical deposition is perhaps the most promising of the available synthesis methods so far. Hence, a thorough analysis of the published data reveals that the synthesis method and alloying and/or doping of Ni play a significant role in the promotion of the HOR electrocatalysis on Ni-based materials. However, a systematic study is still needed to identify promising directions for the further improvement of Ni-based catalysts for the HOR as well as less promising ones.

A significant aspect of Ni-based catalysts is that their limited activity implies larger overpotentials than Pt electrodes for a given current and surface area. A larger overpotential may, in turn, lead to the formation of Ni hydroxide,^31,32^ which is inactive for the HOR.^15^ Therefore, one needs to ensure in operando that the catalyst will stay metallic below this potential range, either by design or by imposing operational constraints. In addition to investigating the effect of alloying elements’ catalytic activity per se, it is therefore also of interest to investigate whether this potential range is affected or not.

In this work, we aim to increase the understanding of the role of the synthetic approach in the alloying of the components of the binary Ni–M catalysts (M = Cu, Co, Fe), as well as to elucidate the impact of the alloying degree on the HOR electrocatalysis in alkaline medium. We have therefore synthesized two series of carbon-supported Ni–M catalysts (M = Cu, Co, Fe) with similar nominal compositions using low-temperature chemical and high-temperature solvothermal reduction methods to achieve these goals. Below we compare the structure, composition, and morphology of electrocatalysts made by chemical and solvothermal reduction, respectively, as assessed by X-ray diffraction (XRD), transmission and scanning electron microscopy (TEM), and energy-dispersive X-ray spectroscopy (EDS). The differences in the chemical (oxidation) state of the catalyst surfaces were therefore assessed by X-ray photoelectron spectroscopy (XPS), hydrogen temperature-programmed reduction (H_2_-TPR), and cyclic voltammetry (CV), for both electrocatalysts made by both chemical and solvothermal reduction. The chemical state of the solvothermally reduced catalysts was characterized by means of in situ Raman spectroscopy. The comparison of the electrocatalytic activity of the materials in the HOR is made with the use of conventional thin-layer rotating-disc electrodes (RDEs) in liquid alkaline electrolytes. Finally, the electrochemical parameters of the catalysts are analyzed in terms of microkinetic models recently proposed in the literature.

## Experimental Section

### Synthesis of the Catalysts

#### Chemical Reduction

Monometallic Ni/C and bimetallic Ni_3_M/C carbon-supported electrocatalysts were synthesized via the chemical reduction (CR) method at 0 °C, using sodium borohydride as the reducing agent.^19,20^ To synthesize monometallic catalyst, 100 mg of VXCMAX22 (Cabot, BET (Brunauer–Emmett–Teller) surface area ca. 1500 m^2^ g^–1^^33^ carbon black), denoted as C, was suspended in 15 mL of isopropyl alcohol (HPLC Plus GC, 99.9%, Sigma-Aldrich) in an ultrasound bath (XUBA3, Grant Instruments) and then mixed with 15 mL of aqueous solution containing 1.7 mmol of NiCl_2_·H_2_O (99.3%, Alfa Aesar). The mixture was cooled in an ice bath and deaerated by flowing Ar (99.999%, Maxima). For the synthesis of bimetallic Ni_3_M/C catalysts, 15 mL of the solutions containing 0.567 mmol of either FeCl_2_·4 H_2_O (99.95%, Sigma-Aldrich), CoCl_2_·6 H_2_O (99.99%, Alfa Aesar), or CuSO_4_·5 H_2_O (for analysis, Merck) was added to the carbon–NiCl_2_ mixture. The intended weight ratio of nickel to carbon in monometallic Ni/C catalyst was 1:1. The same Ni/C weight ratio was kept for the bimetallic Ni_3_M/C catalysts, whereas the atomic ratio of Ni/M was 3:1. An ice-cold solution containing 3.4 mmol of NaBH_4_ (99.99%, Sigma-Aldrich) in 25 mL of 0.01 mol dm^–3^ KOH (AR, BioLab) was used as the reducing agent. The reduction of the metal precursors was carried out in the ice bath by dropping NaBH_4_ solution into the mixture while stirring. The precipitates were separated and rinsed by milli-Q H_2_O (18.2 MΩ cm) in the centrifuge (Eppendorf 5804) five times at 10 000 rpm for 10 min. The samples were placed in the vacuum oven (1407-2, MRC) at room temperature and then dried at 80 °C for 24 h. Before the samples were removed from the vacuum, the oven was cooled back to room temperature. The samples were stored in desiccators under vacuum and handled in the air. Three batches of each catalyst were synthesized to guarantee the reproducibility of the synthetic procedure. The samples synthesized by chemical reduction are denoted as Ni/C-CR and Ni_3_M/C-CR.

No measures were taken to passivate the samples,^15^ which were exposed to air during handling and before being stored in a dry desiccator under vacuum.

#### Solvothermal Reduction

All chemicals and materials were used as purchased and without further purification. Nickel acetylacetonate (Ni(acac)_2_, 95%), cobalt acetylacetonate (Co(acac)_2_, 97%), iron acetylacetonate (Fe(acac)_2_, 97%), oleylamine (OA, 70%), and trioctylphosphine (TOP, 97%) were purchased from Sigma-Aldrich. Vulcan XC-72 (Cabot) carbon with a surface area of 232 m^–2^g^–1^ was used as a support.

The carbon-supported monodispersed Ni/C and Ni_3_M/C (M = Co, Cu, and Fe) were synthesized by a solvothermal reduction (STR) method under an Ar atmosphere in a round-bottom flask attached to a Schlenk line. The size-tunable nanoparticles were achieved by a solution-phase synthesis using OA as a solvent and reducing agent and TOP as a stabilizing agent. The synthesis procedure was adapted from a previously reported procedure for the monodispersed Ni nanoparticles.^34^ Briefly, carbon-supported Ni nanoparticles were prepared by transferring carbon black and Ni(acac)_2_ (3.4 mmol) to a 100 mL round-bottom flask followed by the addition of OA (63.8 mmol) and TOP (20.17 mmol) except for one Ni sample (number 5, see below) for which the amount of TOP added was reduced to 6.8 mmol. The reaction mixture was degassed at 100 °C for 30 min in order to remove any moisture and then heated to 210 °C at a heating rate of 5 °C min^–1^. The mixture was kept at 210 °C for 45 min. The reaction mixture was maintained under an Ar atmosphere during the whole process. The solution was subsequently cooled to room temperature and transferred to a centrifuge tube and washed multiple times with toluene and isopropyl alcohol and finally with toluene and acetone. The synthesis resulted in a fine powder (as-prepared catalyst), which was then dried under vacuum overnight. The carbon-supported Ni nanoparticles were annealed at 500 °C for 2 h under H_2_/Ar (5 vol %) mixture to avoid immediate surface oxidation of the very air-sensitive Ni particles and also to increase the crystallinity. The bimetallic carbon-supported nanoparticles were also synthesized in a similar way as described above keeping the Ni/M weight ratio constant as 3:1 with the total metal loading of 50 wt % in the Ni_3_M/C catalyst. The samples made by solvothermal reduction are denoted as Ni/C-STR and Ni_3_M/C-STR. The thermal treatment at 500 °C in this case had the additional function of alloying the metal components.

As for the samples prepared by chemical reduction, the samples prepared by solvothermal reduction were also handled in air, and no measures were taken to passivate them.

### Physical and Chemical Characterization

Transmission electron microscopy (TEM) images were obtained on either a FEI Tecnai T20 LaB_6_ or JEOL JEM-2100F field-emission gun (FEG) microscope operated at 200 kV. The catalyst powders were dispersed in isopropyl alcohol (2 mg of catalyst per 10 mL) in an ultrasound bath for 2 h and spray-cast onto a Cu grid coated with holey carbon (300 mesh, Agar Scientific) and left to dry on the TEM grids at room temperature.

The particle size histograms were obtained either from the TEM images, which were collected with a FEI Tecnai T20 at 200 kV or from scanning transmission electron microscopy (STEM) images collected with an S-5500 Hitachi at an acceleration voltage of 30 kV.

Energy-dispersive X-ray spectroscopy (EDS) and element maps were collected on a Zeiss Ultra-Plus high-resolution scanning electron microscope (HR-SEM) or JEOL JEM-2100F. The EDS spectra were collected at an accelerating voltage of 12 kV with the data collecting time in the range from 50 through 150 s. The STEM elemental mapping was performed with an acceleration voltage of 20 kV with the samples dispersed on holey carbon 200 mesh Cu TEM grids (Agar Scientific).

X-ray diffraction (XRD) data were collected using either a Rigaku SmartLab diffractometer with Cu X-ray source (λ = 0.154 06 nm) or a Bruker D8 A25 DaVinci X-ray Diffractometer (λ = 0.1548 nm). On the Rigaku instrument the diffractograms were recorded at medium resolution in a parallel beam geometry at a tube current of 150 mA and a tube voltage of 45 kV in θ/2θ scan mode with a scan rate of 1°  min^–1^ in 0.01° steps in a range of diffraction angles from 20 to 80°. Powder X-ray diffraction measurements on the Bruker instrument were performed with an increment of 0.013° in the same range as on the Rigaku instrument. A 20 mm diameter single-crystal Si plate was used as the sample holder to minimize the background. Phases were identified via matching with the International Centre for Diffraction Data (ICDD) PDF4+ (2017) database and the Inorganic Crystal Structure (ICSD) database. Crystallite sizes of the metallic nanoparticles were estimated using Scherrer’s equation.

X-ray photoelectron spectroscopy (XPS) measurements were performed in UHV (2.5 × 10^–10^ Torr base pressure) using either a 5600 Multi-Technique System (PHI, USA) or an Axis Ultra DLD (Kratos Analytical). The samples were irradiated with an Al K monochromated source (1486.6 eV), and the outcoming electrons were analyzed by a spherical capacitor analyzer using a slit aperture of 0.8 mm. Survey spectra were registered in a wide energy range (0–1400 eV) at a low resolution. A pass energy of 160 eV was used for survey scans. In addition, region scans were conducted at a pass energy of 20 eV using a step size of 0.1 eV. Utility multiplex spectra were taken for different peaks in a low energy range window at an intermediate (utility) resolution. Atomic concentration was calculated for all the elements present. The accuracy of the calculation of atomic concentration (AC) was ±2, ±5, ±10, and ±20% for atomic concentrations around 50, 20, 5, and 1%, respectively. The measured spectra were analyzed using either Casa XPS (version 2.3.19) or XPS peak software, and a Lorentzian asymmetric (LA) line shape was used for each component.

The H_2_ temperature-programmed reduction (H_2_-TPR) profile for the materials was obtained using an AutoChem 2920 (Micromeritics) chemisorption analyzer. A thermal conductivity detector (TCD) was used to determine the H_2_ concentration. The sample (ca. 0.1 g) was placed in a quartz reactor, which was then placed in the isothermal zone of a heating furnace. Physisorbed water, if any, was removed by heat-treating the samples at 200 °C for 60 min under an argon flow of 50 mL min^–1^. Afterward, the gas was switched to a mixture of 10 vol % H_2_ in Ar with a flow rate of 50 mL min^–1^ and kept under this flow until a stable baseline for the TCD signal was obtained. Meanwhile, the temperature of the furnace was brought back to room temperature. Once the baseline had been stabilized under the same continuous flow (50 mL min^–1^) of the gas mixture, the temperature of the furnace was increased from room temperature to 800 °C at a rate of 10 °C min^–1^ and subsequently cooled in the Ar flow. The TPR profiles obtained were deconvoluted using a skewed log-normal distribution and the peak areas.

Ex situ Raman spectroscopy was performed with the same instrument as the in situ Raman experiments; see below.

### Electrochemical Characterization

The inks of samples synthesized by chemical reduction were prepared by dispersing 10 mg of the catalyst in 2 mL of an isopropyl alcohol/water mixture (3:1 vol.). A Nafion suspension (10 wt % in H_2_O, density ρ = 1.05 g mL^–1^, Sigma-Aldrich) was added to the catalyst ink to obtain the Nafion:catalyst weight ratio of 1:4. Although Nafion may not be the ideal ionomeric material for measuring the highest catalytic activity of these catalysts,^35^ for the purposes of comparison between the catalysts and to reach the goals of this study, it is indeed acceptable. The suspension was drop-casted on a GC RDE to form a catalyst layer with a loading of 250 μg cm^–2^. The electrode was dried in air for about 1 h and mounted on the rotating shaft of the RDE rotator. To prepare the ink of catalysts prepared by solvothermal reduction, 5 mg of the catalyst powder was suspended in a mixture of H_2_O (250 μL), isopropyl alcohol (250 μL), and 30 μL of the Nafion suspension (5 wt % in aliphatic alcohol–water solution, equivalent weight of 1100, Sigma-Aldrich). After sonication for 30 min, an aliquot of 21 μL (for Ni/C-STR) and 28 μL (for the Ni-M/C-STR catalysts) of the suspension was dropped onto the GC electrode to obtain the catalyst loading of 1.01 mg cm_geom_^–2^ and 1.35 mg cm^–2^ for the monometallic and the bimetallic catalysts, respectively.

Electrochemical measurements were carried out at room temperature in 0.1 mol dm^–3^ KOH using either an Ivium-n-Stat Potentiostat or a WaveDriver 20 Bipotentiostat/Galvanostat (Pine Research) in a three-electrode electrochemical cell with separated compartments. The measurements were performed in a Teflon cell to avoid the Si and other contamination from the glass components. A glassy carbon (GC) electrode (0.196 cm^2^ geometrical surface area, Pine) embedded in a Teflon tip was used in this study as the working electrode. The GC electrode was polished prior to use with Gamma Micro Polish Alumina (0.05 μm), after which the electrode was rinsed with Milli-Q water and finally dried in air. Hg/HgO/4.2 mol dm^–3^ KOH was used as the reference electrode, and a Pt foil or wire served as the counter electrode. All the potentials in this work are presented versus reversible hydrogen electrode potential (RHE). Hydrogen, 99.999% purity was used for the HOR experiments, and argon, 99.9999% purity was used as the inert gas.

The following protocol was elaborated for the samples synthesized by chemical reduction.^19,20^ Before the RDE working electrode was immersed into the electrolyte, the electrolyte was purged (saturated) with H_2_ (flow 0–250 mL min^–1^) until the open circuit potential had stabilized. The potential was then scanned repeatedly (up to five cycles) in the potential range between 0 and 0.4 V at a sweep rate of 1 mV s^–1^ while the electrode was rotated at an angular velocity of 1600 rpm, from which the HOR kinetics were inferred. Afterward, the gas flow was changed to Ar until the open circuit potential was stabilized, and then the potential was swept in the potential range between 0 and 0.4 V at a rate of 1 mV s^–1^. Electrochemical preactivation via potential cycling within −0.2 and 0.4 V did not result in a catalytic activity improvement and was therefore not applied to the CR catalysts. The reproducibility of the collected experimental data was ensured by three to five repetitive measurements for every single batch of each catalyst.

For the catalysts made by solvothermal reduction, the working electrodes were preconditioned by sweeping the potential between −0.2 and 0.4 V vs RHE in an Ar-purged electrolyte kept under an Ar atmosphere at a sweep rate of 20 mV s^–1^ in order to minimize the effects of surface passivation on the catalysts (see the Supporting Information). CVs collected under similar conditions (argon purging) and after the preconditioning at 1 mV s^–1^ are reported here. Later, the electrolyte was purged (saturated) with H_2_, and the HOR polarization curves were measured at a scan rate of 1 mV s^–1^ and with the electrode rotating at 1600 rpm.

The CVs were integrated in the range of the Ni(OH)_2_ formation and used as an in situ method to determine the electrochemically active surface area (ECSA) of Ni with the specific charge density of 514 μC cm^–2^.^36^ Details of the determination of the ECSA are given in the Supporting Information. The exchange current density (*i*_0_) values were calculated in the micropolarization potential range (−10 through 50 mV for the CR samples, -10 through 10 mV for the STR samples) applying the following equation
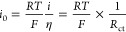
1where *i* is the current density (in A cm_Ni_^–2^) averaged for the range of overpotential values (η) between −10 and 50 mV (10 mV), *T* is the temperature of the electrolyte (K), *R* = 8.314 J mol^–1^ K^–1^ is the gas constant, *F* = 96 485 C mol^–1^ is Faraday’s constant, and *R*_ct_ is the charge transfer resistance (Ω cm^2^). (This equation derives from the Butler–Volmer equation for outer-sphere reactions with one electron transferred and assumes that the charge transfer coefficients add up to one. The equation is not generally applicable for multielectron, electrocatalytic reactions such as the hydrogen-evolution and -oxidation reactions. However, in order to enable a comparison with previous works that do use it, it is also adopted here as a parametrization of catalyst activity. The conditions under which the Butler–Volmer equation applies for the hydrogen-evolution/-oxidation reactions are outlined by Shinagawa et al.^37^).

### In Situ Raman Spectroscopy

In-situ Raman spectroscopy was performed in a specially designed (in-house) cell of Teflon fitted with a quartz window through which the laser beam from a Witec alpha300 R Confocal Raman imaging system (equipped with Zeiss EC Epiplan 10× objective lens) was admitted toward the sample.^38^ The laser power was 20 mW, and the laser wavelength 532 nm. The measurements were made in Ar-saturated 0.1 mol dm^–3^ KOH (semiconductor grade, 99.99% purity, Sigma-Aldrich). Catalyst deposited on glassy carbon, a graphite rod (Pine Research), and a Hg/HgO (Pine Research) electrode were used as working, counter, and reference electrodes, respectively. The catalysts were activated by cycling at a potential range of −0.2 to 0.4 V vs RHE for 50 cycles at 100 mV s^–1^. The Raman spectra were collected in situ at 0, 0.4, and 0.5 V vs RHE for 1000 s (100 accumulations) after the collection ex situ.

## Results and Discussion

### Catalyst Structure and Morphology

**Figure 1:**
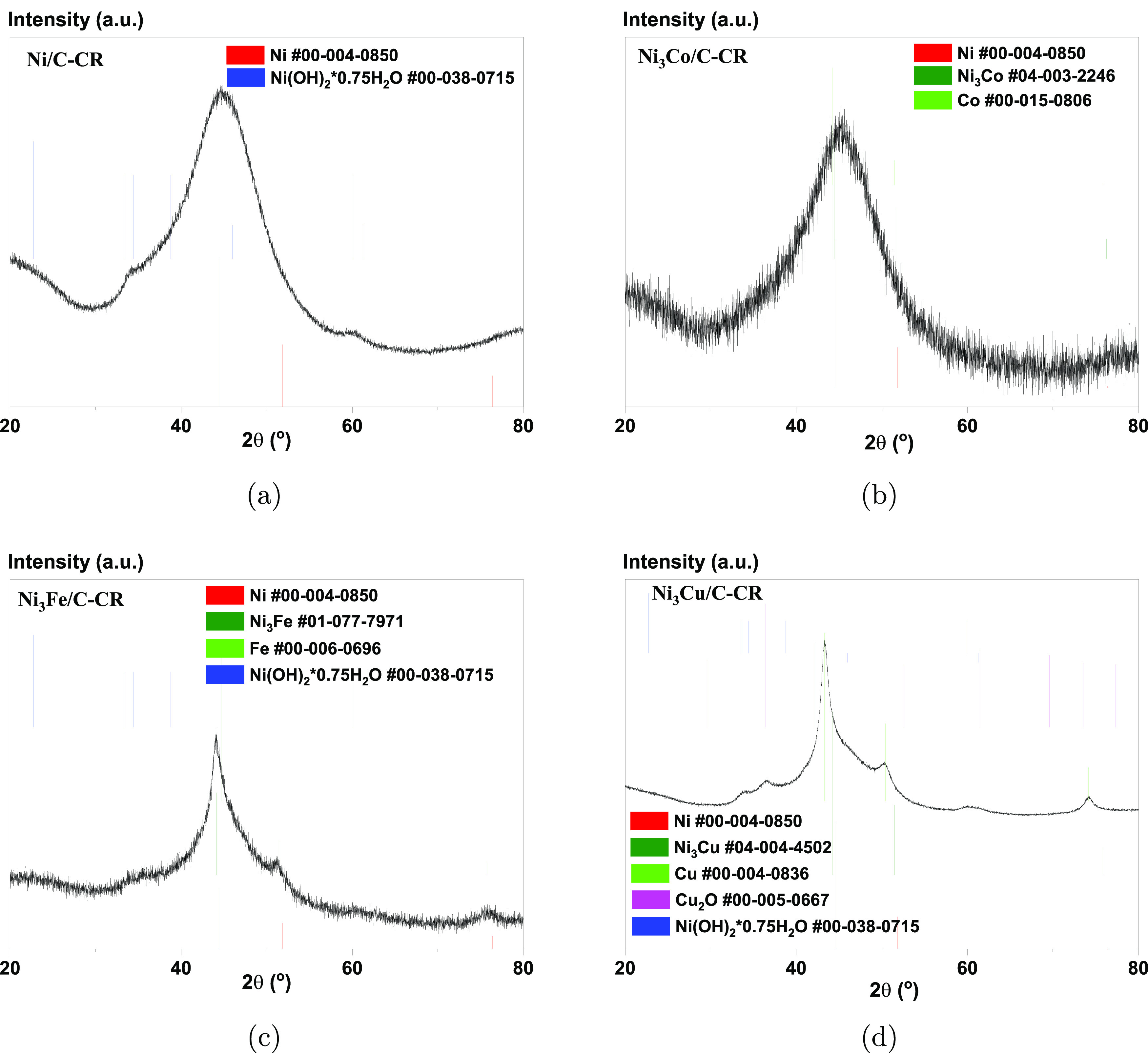
XRD patterns for the catalysts prepared by chemical reduction: (a) Ni/C-CR, (b) Ni_3_Co/C-CR, (c) Ni_3_Fe/C-CR, and (d)Ni_3_Cu/C-CR.

Figure 1 shows the characteristic XRD patterns of the catalysts synthesized by chemical reduction and features a broad main peak at ca. 45°. This peak can be assigned to the (111) diffraction peak for metallic face-centered cubic (fcc) Ni (PDF #00-004-0850). The (111) diffraction peak may overlap with and thus conceal the corresponding diffraction peaks of Ni–M alloys (#01-077-7971 for Ni_3_Fe—Figure 1c; #04-003-2246 for Ni_3_Co—Figure 1b) or of the dopant by itself (#00-006-0696 for Fe—Figure 1c; #00-015-0806 for Co—Figure 1b; #00-004-0836 for Cu—Figure 1d). The wide broadening of the (111) line is due to the small crystallite sizes, approximately 1.1–1.5 nm (Table 1). The characteristic (200) and (220) lines of fcc Ni do not appear in the diffraction patterns of Ni/C-CR, Ni_3_Co/C-CR, and Ni_3_Cu/C-CR for the samples prepared by chemical reduction. However, from the diffractogram for Ni_3_Fe/C-CR, we can see some indications of the (200) and (220) reflections of either fcc Ni, an fcc Ni–Fe alloy, or their mixture. The sharp peaks observed in Ni_3_Cu/C-CR (Figure 1d) correspond either to metallic Cu or Cu_2_O (#00-005-0667).

**Table 1:** Statistical Parameters of the Catalysts Derived via Chemical and Solvothermal Reduction

synthesis	chemical reduction				solvothermal reduction			
parameter	Ni/C	Ni_3_Fe/C	Ni_3_Co/C	Ni_3_Cu/C	Ni/C	Ni_3_Fe/C	Ni_3_Co/C	Ni_3_Cu/C
*L* (nm)^a^	1.5	1.4	1.1	1.3	5.1	10.0	10.8	9.2
Δ*d* (nm)^b^	3–19	3–33	3–61	3–26	4–12	10–18	7–43	9–25
*d*_av_ (nm)^c^	11 ± 3	15 ± 3	23 ± 3	14 ± 3	8 ± 3	14 ± 3	21 ± 3	17 ± 3
*S*_TEM_ (m^2^ g_Ni_^–1^)^d^	63	45	29	50	84	50	32	39

a Crystallite size calculated from the Scherrer equation for the XRD diffraction peak for Ni(111): *L* = *Kλ*/β cos θ, where *K* ∼ 0.9 is a shape factor, λ is the wavelength for Cu *Kα* radiation, β is the full width at half-maximum (fwhm, in radians), and θ is the Bragg angle (in radians).

b Δ*d*: range of particle diameters based on the TEM image analysis.

c *d*_av_: average diameter calculated using the equation .

d *S*_TEM_: total surface area calculated based on the TEM images using the equation ; ; *N*: number of particles; *S*_*i*_: surface of all the particles with the diameter *d*_*i*_; ρ = 8.9 × 10^6^ g m^–3^ (density of Ni); *w*_*i*_: mass fraction of the particles with the diameter *d*_*i*_; *N*_*i*_: number of particles with the diameter *d*_*i*_; *m*_*i*_: mass of one particle with the diameter *d*_*i.*_.

**Figure 2:**
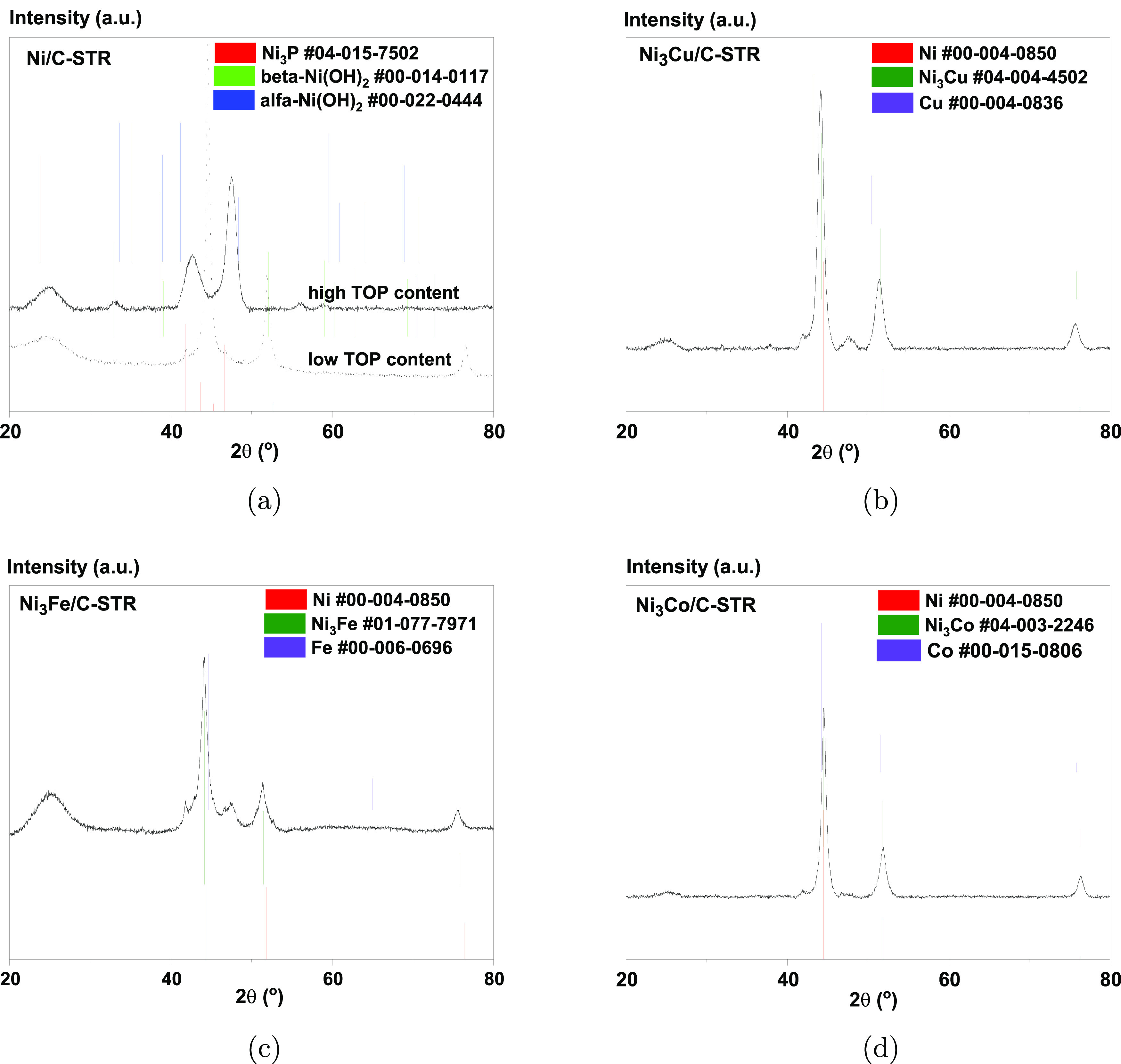
XRD patterns for the catalysts made by solvothermal reduction: (a) Ni/C-STR, (b) Ni_3_Cu/C-STR, (c) Ni_3_Fe/C-STR, and (d) Ni_3_Co/C-STR.

Figure 2 shows the XRD pattern for Ni/C-STR, Ni_3_Co/C-STR, Ni_3_Cu/C-STR, and Ni_3_Fe/C-STR. The (111) peak for Ni at 2θ = 44.5° is visible for Ni_3_Cu, Ni_3_Fe, Ni_3_Co, and the Ni/C sample prepared with 6.8 mmol TOP (labeled “low TOP content” in Figure 2A). For the monometallic Ni/C-STR sample prepared with 20.17 mmol TOP (labeled “high TOP content” in Figure 2A) the Ni(111) peak is absent. Instead, two peaks appear at approximately 43° and 47°. Both these peaks are discernible in all samples after the thermal treatment but only to an almost negligible extent in the Ni_3_Fe, Ni_3_Cu, and Ni_3_Co samples and the Ni/C sample prepared with the lower amount of TOP. In the sample with the higher content of TOP, lattice contraction is not a likely explanation for the shift of the (111) peak to ca. 47°, since this would correspond to a very large lattice contraction (on the order of Å/deg). (By comparison, Sheng et al.^39^ found that Ni particles contract by approximately 0.01% down to 50 nm and then dilate back to approximately the bulk lattice constant at around 25 nm.) The peak at 2θ ≈ 47° in Figure 2 is, however, consistent with the formation of Ni_3_P from Wang et al.^40^ (PDF #04-015-7502) and other phosphides^41^ (see also Figures S10 and S12 in the Supporting Information). Comparison of the XRD patterns for the catalysts with those of the corresponding nickel–metal alloys allows us to assume that the solvothermal reduction results in alloying of Ni with the secondary metal M (M = Fe, Cu, Co).

**Figure 3:**
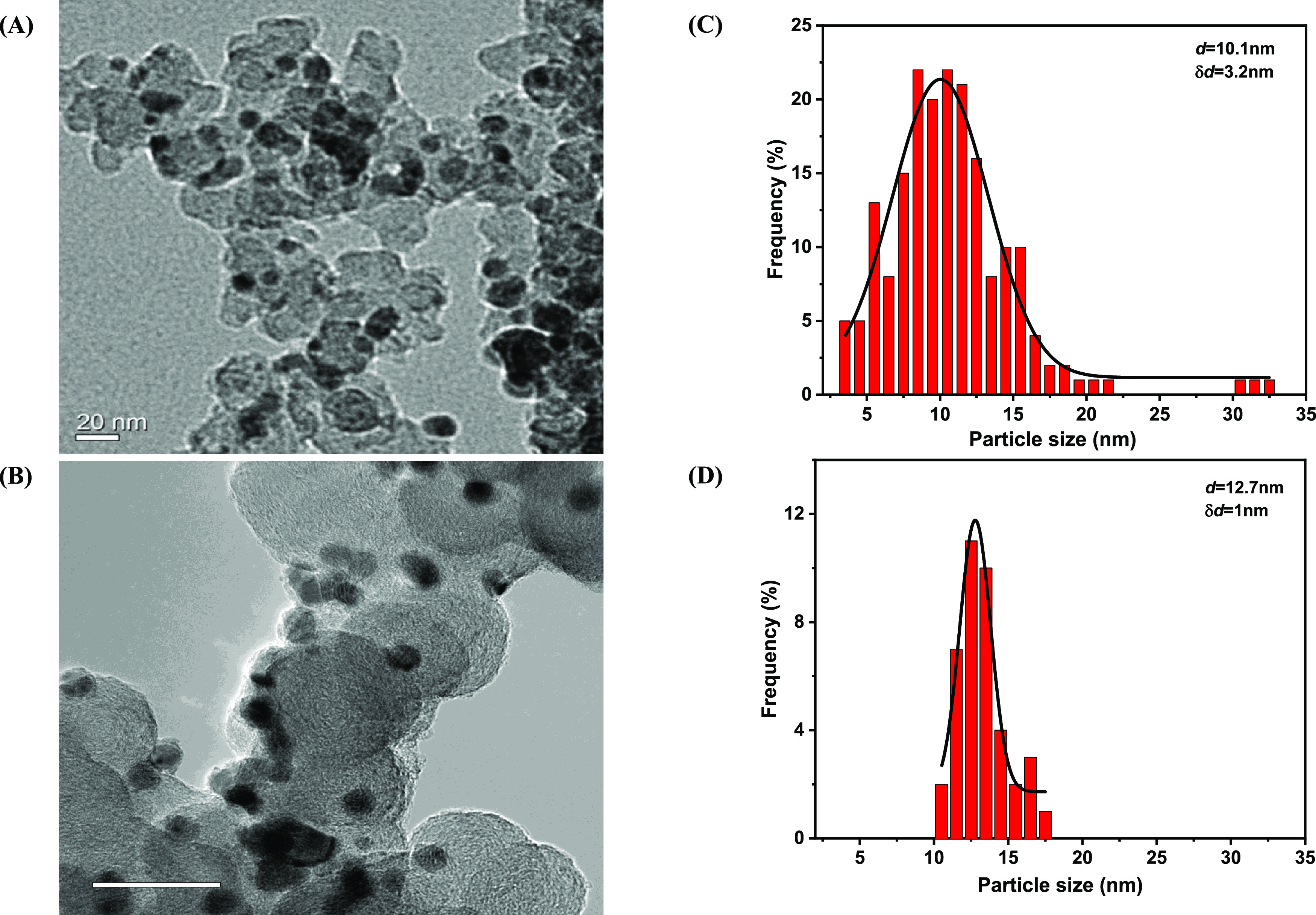
TEM images (A, B) and the corresponding particle diameter distribution histograms (C, D) for Ni_3_Fe/C-CR (A and C) and Ni_3_Fe/C-STR (B and D) catalysts. *δd* denotes the standard deviation.

Figure 3 shows representative TEM images and particle size distributions for Ni_3_Fe/C catalysts prepared by chemical and solvothermal reduction. The TEM images and particle size distributions for the other catalysts, i.e., Ni/C, Ni_3_Cu/C, and Ni_3_Co/C, both for the CR and STR syntheses, are provided in Figures S1–S4 in the Supporting Information. The samples synthesized by chemical reduction generally exhibited a wider particle size distribution compared to those made by solvothermal reduction (Table 1).

Table 1 reports the crystallite and particle size-related parameters for reduced samples made by both chemical and solvothermal reduction. For both series, the Ni/C samples have the highest surface areas as estimated by TEM, 62.6 and 83.9 m^2^ g_Ni_^–1^, respectively. The lowest surface area values are displayed by the cobalt-containing samples, Ni_3_Co/C-CR (28.8 m^2^ g_Ni_^–1^) and Ni_3_Co/C-STR (31.8 m^2^ g_Ni_^–1^). Thus, both syntheses, chemical reduction of simple inorganic precursors and reduction of the acetylacetonate precursors with subsequent annealing in H_2_/Ar, result in similar catalyst morphology—near-spherical nanoparticles with similar values for the particle diameters.

The trends in particle size and surface area with composition for the two series of electrocatalysts are the same, and monometallic Ni catalysts tend to form particles that are smaller than those of the Ni_3_Fe and Ni_3_Cu catalysts, which in turn are smaller than the Ni_3_Co catalyst. The STR synthesis results in a slightly narrower particle size distribution compared to CR, as expressed by a smaller standard deviation for the particle-size distribution, Δ*d*, in Figure 3, Table 1, and Figures S1–S4 in the Supporting Information.

**Figure 4:**
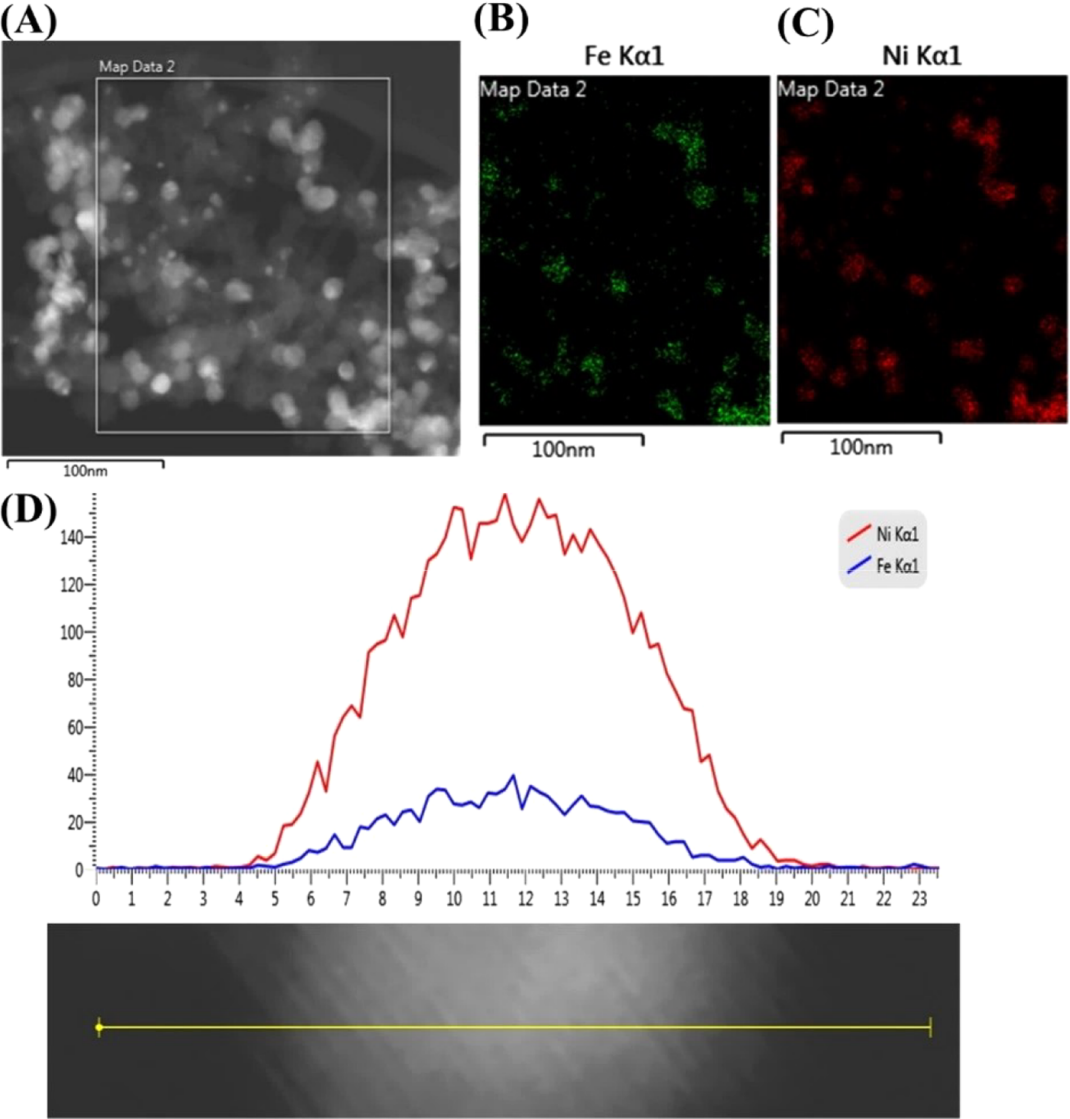
STEM image of an annealed sample of Ni_3_Fe/C-STR nanoparticles (A), EDX elemental maps of Fe (B) and Ni (C), EDX line profiles (D) for Ni (red line) and Fe (blue line). Scale bars in (A)–(C) are 100 nm. The scale bar in (D) is 23 nm.

Figure 4A provides a high-angle annular dark-field scanning transmission electron microscopy (HAAD-STEM) image of the Ni_3_Fe/C-STR catalysts made by solvothermal reduction. The corresponding STEM-EDX element mapping images, Figure 4B–C, show that the elemental distributions of Fe (green) and Ni (red) are superposed. A line-scanning analysis (Figure 4D) indicates that the metallic components form individual Ni–Fe nanoparticles, and the concentration ratio between Ni (red curve) and Fe (blue curve) corresponds approximately to the expected ratio of 3:1. Element mapping obtained by means of high-resolution SEM imaging (Figures S5–S8 in the Supporting Information) of the chemically reduced catalysts shows a fair homogeneity in the codistribution of Ni and the alloying metal.

Our observations of the samples prepared by the two different synthesis procedures may be summarized as

1. Both the STR and CR result in polycrystalline nanoparticles of spherical shape.
2. The STR results in narrower particle size distribution and smaller particle sizes compared to CR.
3. The STR results in a homogeneous Ni-metal codistribution observed at the scale of one nanoparticle.
4. The CR results in a homogeneous codistribution of Ni and the second metal, though at a significantly lower magnification. Some phase aggregation and surface segregation of the metallic components are observed.
5. The CR results in poorly crystalline materials with the crystallite sizes of 1.1–1.5 nm, whereas STR results in crystallites approximately an order of magnitude larger.
6. Thermal annealing of the pristine samples prepared by solvothermal reduction with TOP as a stabilizing agent may form a nickel-phosphide phase such as Ni_3_P. The presence of the second transition metal suppresses the phosphide formation.
7. The STR results in the formation of Ni-metal alloys, whereas CR results in a mixture of phases, most likely comprised of metallic Ni, metallic Cu/Co/Fe and the corresponding oxides (Cu_2_O), and Ni-metal alloys.

### Surface Composition and Surface State

Table 2 shows the mass-averaged composition of the two groups of the electrocatalysts as assessed by EDS. The samples prepared by chemical reduction were intended to have a fixed mass ratio Ni/(Ni + C) of 0.5 (column 2). However, due to a partial passivation in ambient air, the actual averaged Ni/(Ni + C) ratio spans the range 0.34 (for Ni_3_Cu/C-CR) through 0.42 (for Ni_3_Co/C-CR), depending on the degree of oxidation. As seen in Table 2, columns 4 and 8, the highest degree of bulk oxidation is observed for Ni_3_Fe/C-CR and Ni_3_Cu/C-CR, while Ni/C-CR and Ni_3_Co/C-CR appear to be less prone to the atmospheric oxidation. The opposite tendency is observed for the STR samples: Ni_3_Fe/C-STR and Ni_3_Cu/C-STR are the less oxidized catalysts, and monometallic Ni/C-STR is the most sensitive to oxidation.

**Table 2:** Mass Composition of the Catalysts Characterized by the EDS Method

				element concentration/wt %^a^				
catalyst	theoretical Ni/(Ni + C)	actual Ni/(Ni + C)	actual O/(Ni + M)	Ni	M	C	O	P (for STR)
Ni/C-CR	0.5	0.37	0.088	34.56	-	57.85	3.07	–
Ni_3_Fe/C-CR	0.5	0.39	0.25	31.57	9.13	48.64	10.27	–
Ni_3_Co/C-CR	0.5	0.42	0.092	32.27	13.14	44.08	4.20	–
Ni_3_Cu/C-CR	0.5	0.34	0.32	26.13	6.88	49.98	10.40	–
Ni/C-STR	0.5	0.43	0.16	37.11	-	49.02	6.96	4.79
Ni_3_Fe/C-STR	0.38	0.26	0.10	23.38	5.39	65.94	2.85	2.45
Ni_3_Co/C-STR	0.38	0.43	0.11	35.37	8.97	47.62	5.01	3.03
Ni_3_Cu/C-STR	0.38	0.38	0.03	31.84	12.11	51.06	1.17	3.82

a The Kα line of B (0.185 eV) in the EDS spectra overlaps with the Kα line of C (0.277 eV).

**Figure 5:**
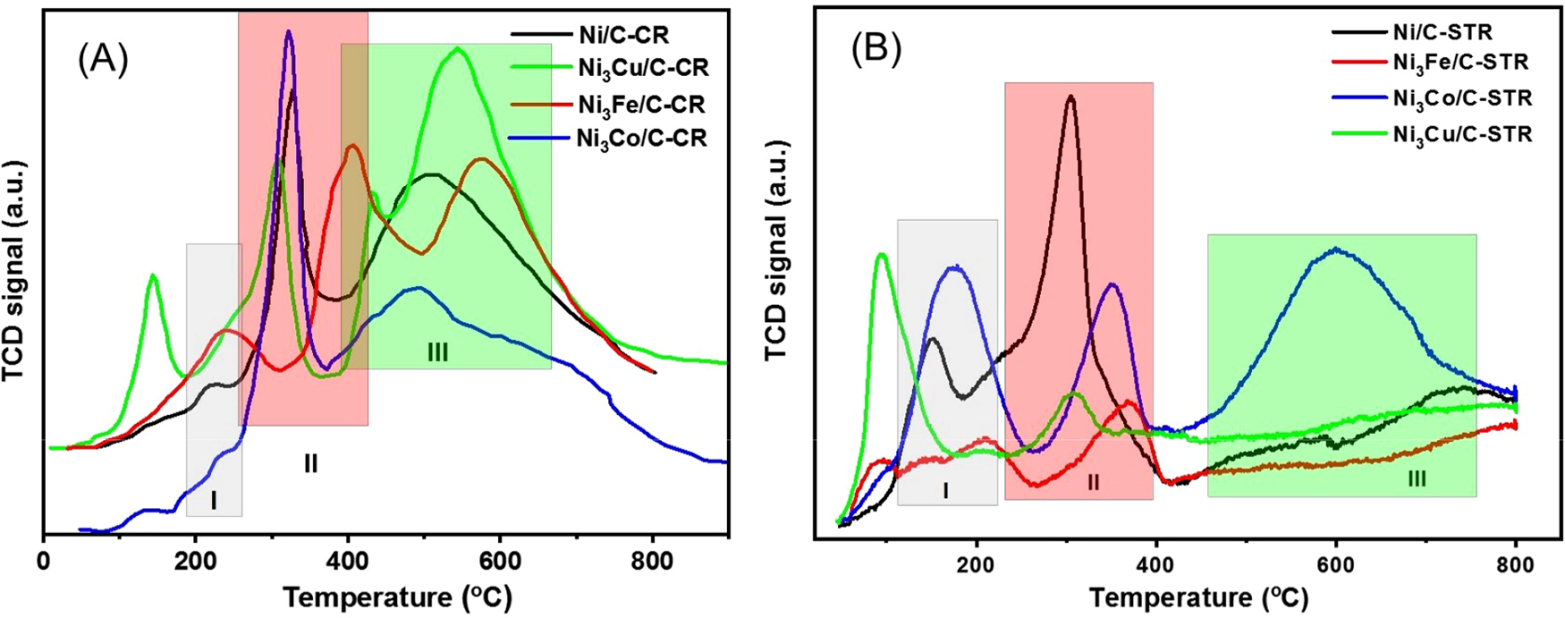
H_2_-TPR profile (the signal from the thermal conductivity detector (TCD) vs temperature) for the (A) catalysts prepared by chemical reduction and (B) catalysts synthesized by solvothermal reduction.

Figure 5 presents H_2_-TPR results for samples of Ni/C, Ni_3_Co/C, Ni_3_Cu/C, and Ni_3_Fe/C made by chemical reduction (Figure 5A) and by solvothermal reduction (Figure 5B). Three main temperature zones (I, II, and III) can be distinguished from the H_2_-TPR spectra in Figure 5. Zone I corresponds to the reduction of Ni(OH)_2_ as reported in our previous work.^20^ Peaks in zone II can be assigned to the reduction of NiO.^42,43^ Wide and intensive peaks in zone III, observed for all the CR samples in Figure 5A and for Ni_3_Co/C-STR in Figure 5B, can be ascribed to the reduction of NiO closely interacting with the supporting material.^44^ The TPR profiles of the copper-containing samples, both those synthesized by chemical reduction and those made by solvothermal synthesis (Ni_3_Cu/C-CR and Ni_3_Cu/C-STR), contain some additional low-temperature peaks, which we assign to the reduction of oxidized copper.^45^

The data in Figure 5 indicate better alloy formation for the samples synthesized by solvothermal reduction than for the samples synthesized by chemical reduction. For the Ni/C-CR catalysts synthesized by chemical reduction, the amount of H_2_ consumed for Ni(OH)_2_ reduction (0.296 mmol g_cat_^–1^, Table S1 in the Supporting Information) is lower than the amount of H_2_ for the Ni/C catalysts synthesized by solvothermal reduction (0.560 mmol g_cat_^–1^, Table S2). Thus, H_2_-TPR measurements indicate that the solvothermal synthesis results in a higher concentration of Ni(OH)_2_ at the catalyst surface. Zone II (Figure 5A) for the Ni_3_Cu/C samples prepared by chemical reduction is slightly shifted to lower temperatures compared to bare Ni/C synthesized by chemical reduction, which might indicate some interaction between the oxides of Ni and Cu. The peaks II and III (Figure 5A) for Ni_3_Fe/C prepared by chemical reduction are shifted to a higher reduction temperature, which indicates Ni–Fe alloy formation. In the case of the catalysts synthesized by solvothermal reduction, all the peaks I and II are shifted depending on the ligand (alloying) metals Cu, Co, or Fe. This is a clear indication of alloying. The most prominent shift appears for Ni_3_Fe/C (Figure 5B), similarly to Ni_3_Fe/C prepared by chemical reduction (Figure 5A). Thus, the H_2_-TPR results agree with the XRD patterns and confirm the solvothermal synthesis results in alloying of Ni with the ligand metals.

Another important conclusion is that doping with Fe results in the formation of more thermally stable Ni(OH)_2_ and NiO. However, the degree of oxidation is less than with the other ligand metals or in bare Ni, as follows from the overall amount of H_2_ needed for the full reduction of the catalysts. The H_2_ consumption increased in the order Ni_3_Fe/C by STR < Ni_3_Fe/C by CR < Ni/C by STR < Ni/C by CR; see Tables S1 and S2 in the Supporting Information for details.

The wide and intensive peaks in the high-temperature zone III (Figure 5A) characteristic for the samples prepared by chemical reduction reveal significant chemical interaction between the catalyst nanoparticles and the carbon support; the peaks in the H_2_-TPR profiles can only stem from oxidized parts of the sample. For unsupported nickel oxide, only one single peak at 400 °C is usually observed in the profile.^46^ A peak at higher temperatures for supported nickel oxide can only mean that this oxide is more difficult to reduce than the unsupported oxide and, in turn, that this comes about by an interaction between the oxide and the support. The detailed nature of this interaction is not possible to infer from our H_2_-TPR measurements. It is, however, clear that the interaction is “chemical” in the sense that the significant temperature shift is consistent with chemical bonding. In contrast, the reduction at high temperatures is negligible for the other solvothermally synthesized catalysts, with an exception for Ni_3_Co/C-STR (Figure 5B).

The total amount of hydrogen consumed during the reduction process was in the range 1–4.5 mmol g^–1^ catalyst (c.f. Supporting Information, Tables S1 and S2). Assuming an atom radius of 125 pm and that one surface oxygen atom per metal atom is removed per H_2_, this corresponds to a surface area on the order of 100 m^2^ g^–1^ catalyst, c.f. Table 1.

XPS survey scans confirmed that the catalyst surface contains carbon, nickel, the transition metal, oxygen, and phosphorus for the solvothermally synthesized samples (see Table S7 in the Supporting Information). Boron was not detected in the CR samples by EDS (Table 2) due to the low intensity of the B K_α_ line (0.185 eV) overlapping with that of carbon (0.277 eV). It is known that B is not easily detected by the EDS, which may lead to errors in the identification; although the XPS data do indicate a small amount of boron in the sample, the amount is presumably too low for detection in EDS. The Ni/M ratios were significantly higher in the surface than in bulk, indicating surface segregation of Ni, especially for Ni–Co, which is in a good agreement with the element maps (Figures S5–S8 in the Supporting Information). The STR samples display a very high tendency of Ni atoms to segregate to the surface (Ni/M ratio in Table S7 in the Supporting Information), likely due to diffusion of the atoms during the thermal annealing. Nguyen et al.^47^ have recently suggested that Cu precursors reduce at a lower temperature (180 °C) than Ni precursors in a solvothermal synthesis, similar to the temperature that is used in this study. (Nguyen et al.^47^ used 220 °C for their synthesis.) However, for the data presented here it is not possible to determine to which extent the segregation occurs during the synthesis or during the subsequent annealing.

A small residual in the XPS spectra for the Ni/C sample synthesized by solvothermal reduction is presumably due to a small amount of NiO not accounted for in the analysis. This is corroborated by the Raman spectroscopy to be reported below. However, as will be shown below, this NiO is reduced to metallic Ni in the activation procedure.

**Figure 6:**
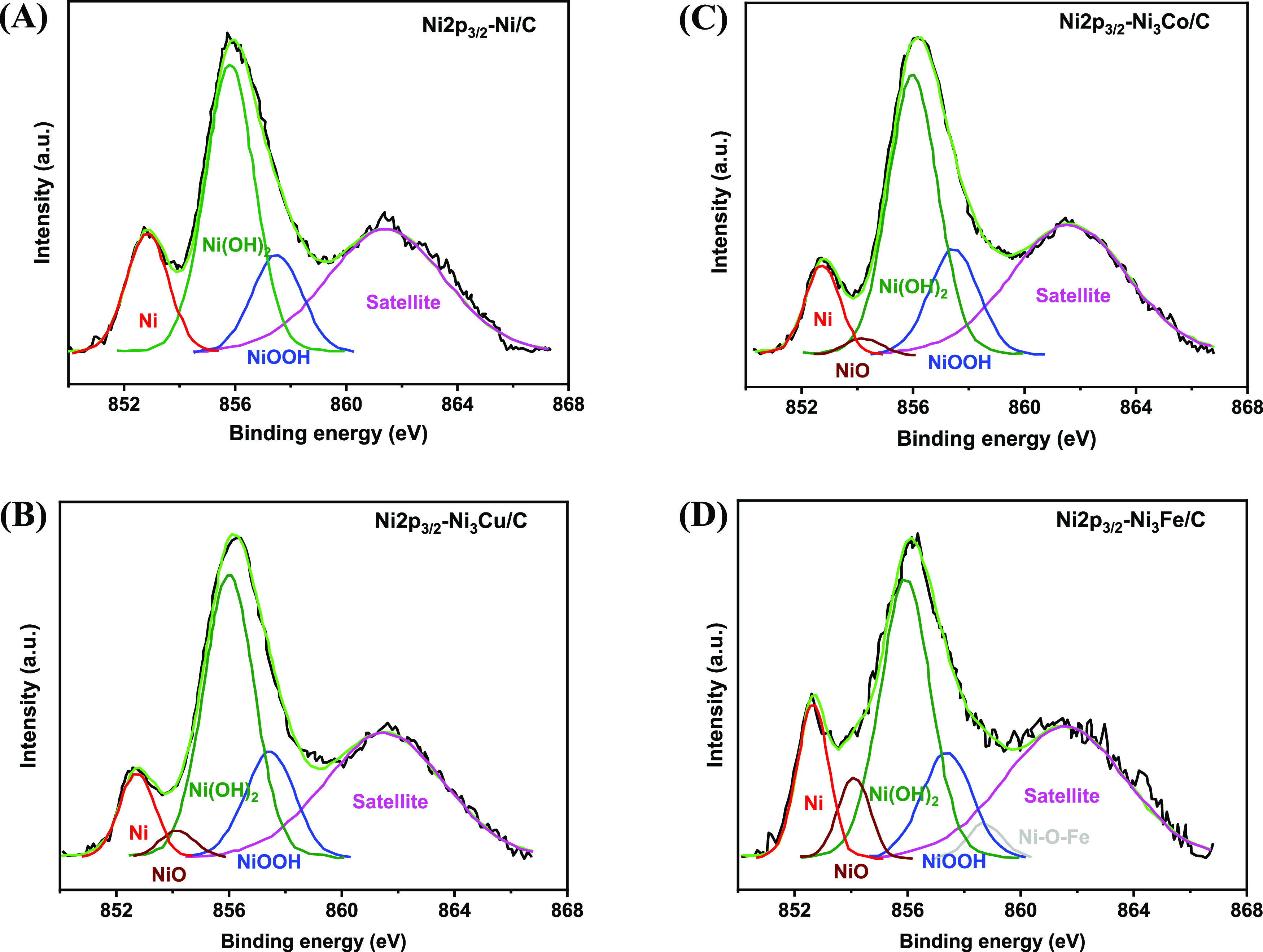
XPS spectra of Ni 2p regions of samples prepared by chemical reduction: (A) Ni/C-CR, (B) Ni_3_Cu/C-CR, (C) Ni_3_Co/C-CR, and (D) Ni_3_Fe/C-CR.

**Figure 7:**
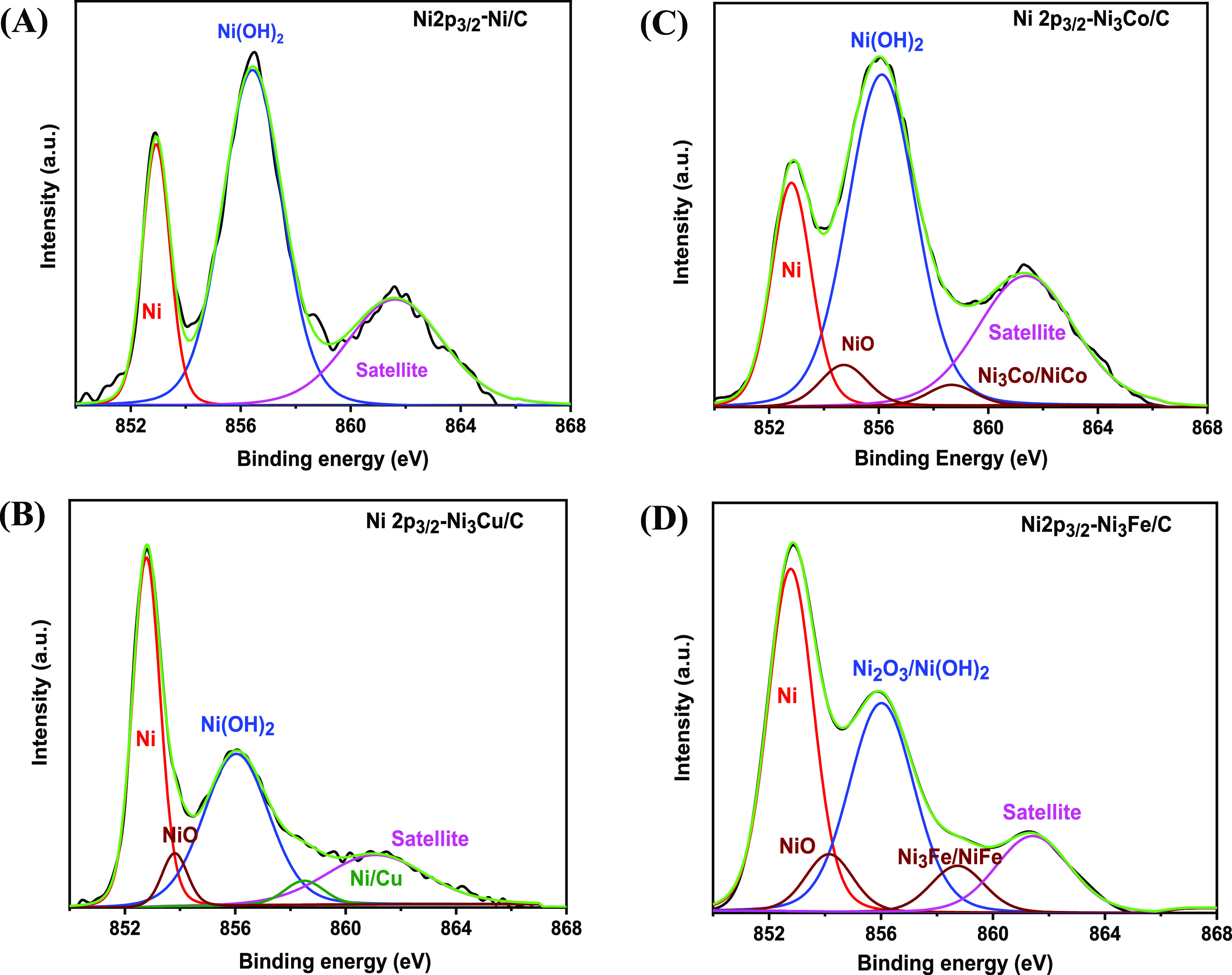
XPS spectra of Ni 2p regions of catalysts synthesized by solvothermal reduction: (A) Ni/C-STR, (B) Ni_3_Cu/C-STR, (C) Ni_3_Co/C-STR, and (D) Ni_3_Fe/C-STR.

Figures 6 and 7 show the deconvolution of Ni 2p XPS spectra for the CR and STR catalysts, respectively. Independent of the synthesis method, the XPS spectra display similar features for the Ni 2p_3/2_ peak. For both samples the XPS data indicate the presence of Ni in oxidation states +2 and +3 in addition to zerovalent, elemental Ni. In agreement with literature data,^48^ the main Ni 2p_3/2_ line for the metallic nickel appears at a binding energy of 852.7 eV. This line was observed at slightly higher binding energies for the monometallic Ni/C-CR (Table S5) and Ni/C-STR (Table S6), which are 852.85 and 852.93 eV, respectively. The upshift of the lines might be related to the presence of boron^49^ in the catalysts manufactured by chemical reduction and phosphorus^50^ in the catalysts manufactured by solvothermal reduction. The presence of boron and phoshorous was confirmed by the XPS and EDS; see details in the Supporting Information. However, a slight downshift of the binding energies for the Ni(0) is observed for all the bimetallic Ni–M catalysts (Tables S5 and S6 in the Supporting Information), which may be related to the fact that the presence of the doping transition metals decreases the yield of boron and phosphorus during the synthesis.

The content of the elemental metallic nickel, Ni(0), is higher in the catalyst manufactured by solvothermal reduction than in those synthesized by chemical reduction (Tables S5 and S6 in the Supporting Information), which is consistent with the EDS and XPS data (Table S7 in the Supporting Information), showing that the STR catalysts are less oxidized. Thus, one might expect higher ECSA values for the STR catalysts compared to those for CR. Peaks corresponding to NiO appear exclusively in the bimetallic catalysts (854 eV), Figures 6 and 7. This indicates that one role of the doping element is to increase the degree of oxidation of Ni. A major constituent of the catalyst surface is Ni(OH)_2_, ranging from 32 to 37 atom % for the CR samples and from 35 to 54 atom % for the STR samples (see the Supporting Information).

**Figure 8:**
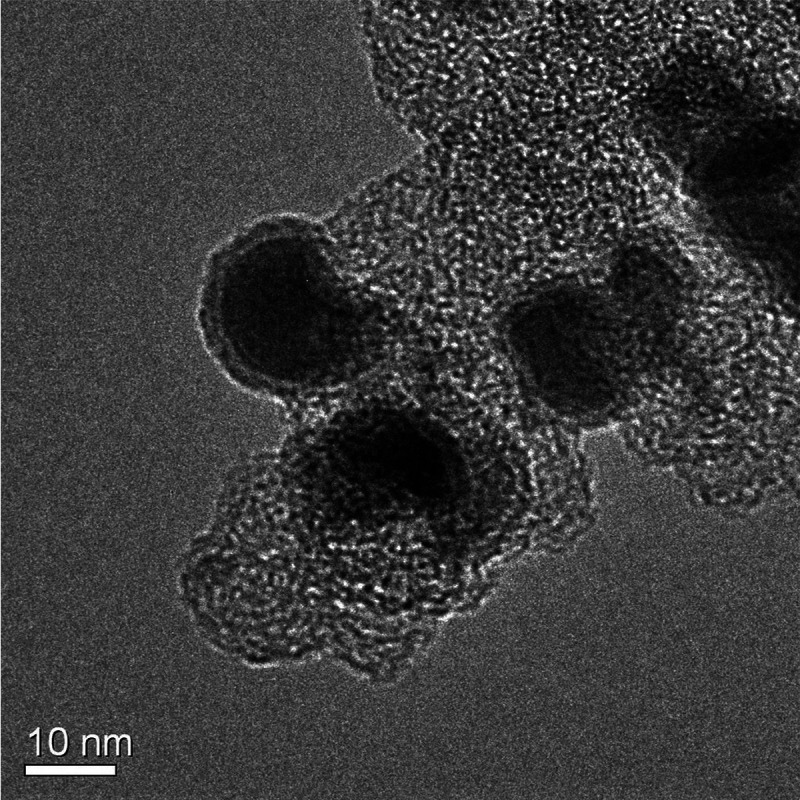
HR-TEM image for the Ni_3_Fe/C-CR catalyst, clearly illustrating passivation layers on the surface of the metallic particles.

The analysis of the oxidation states by XPS (Tables S3–S6 in the Supporting Information) is thus in line with the H_2_-TPR results; the XPS analysis indicates that the samples contain a number of oxidation states of both Ni and the dopant element. However, the surface of the catalysts is more oxidized than the bulk since the EDS data indicate a higher atom ratio of oxygen to total metal (O/(Ni + M)) than do the XPS data (Table S7 in the Supporting Information). The O/(Ni + M) ratios, as evaluated by XPS, exceed 2 for all the CR samples, which indicates a significant degree of surface passivation. This is clearly seen from TEM images, of which an example is given in Figure 8. We thus interpret the bright rim in the images as corresponding to the oxide detected by H_2_-TPR and XPS.

Ex situ Raman results also showed distinctive features associated with NiO. To facilitate comparison, these are presented below together with the in situ data.

The lower O/(Ni + M) ratios observed for the solvothermally synthesized samples than for the CR samples, from both XPS and EDS data (Table S7), are also consistent with the analysis of the Ni p peaks. For the samples prepared by chemical reduction, the atom % of Ni(0) relative to nickel in higher oxidation states ranged from approximately 8 in Ni_3_Cu through 13% in Ni/C, whereas for the catalysts synthesized by solvothermal reduction samples the relative number of reduced Ni was substantially higher, viz. 19% in Ni_3_Co/C through 39% in Ni_3_Fe/C. In conclusion, therefore, the catalyst surfaces are highly oxidized, more in the samples synthesized by chemical reduction than in the samples prepared by solvothermal reduction.

The observations, therefore, indicate that similarities and differences between the CR and STR catalysts are the following:

1. The bulk and surface contents of oxygen are lower in the STR catalysts than in samples prepared by chemical reduction.
2. Both catalyst series have surfaces rich in Ni oxidation products, such as NiO_*x*_ and Ni(OH)_2_. The samples made by chemical reduction also contain a Ni^3+^ component, presumably NiOOH.
3. The Ni atoms in both the CR and STR samples tend to segregate on the surface, which is especially pronounced in the STR samples. In some of the binary catalysts, the Ni/M ratio is so high that one may expect the electrochemical parameters of these binary catalysts to be totally dominated by nickel and therefore to perform similarly to the pure Ni/C catalysts.
4. The synthesis by chemical reduction results in the formation of a mixture of metallic Ni and Ni possibly with a small amount of borides. The STR method results in the formation of the mixture of metallic Ni and some Ni phosphides.
5. Doping by the secondary metal in the CR and STR catalysts results in an increase of the overall oxygen content compared to the Ni/C catalyst.
6. Doping by the secondary metal both in CR and STR catalysts results in the appearance of a NiO phase.
7. In the CR catalysts, unlike the STR samples, there is presumably a significant chemical interaction between the metal-containing phases and the carbon support.

### Electrochemical Characteristics

The XPS analysis above indicates that substantial amounts of Ni oxidation products are present at the sample surfaces. These have a significant impact on the electrocatalytic activity, but some of them, for instance α-Ni(OH)_2_, can be easily reduced electrochemically.^50^ The catalysts were, therefore, activated as described in the Experimental Section prior to the measurements of HOR activity. While the activation had a substantial effect on the STR samples, which were in practice not active for the HOR in the absence of such preconditioning, preconditioning of the CR samples had the opposite effect. Details are given in the Supporting Information, Figures S18 and S19.

**Figure 9:**
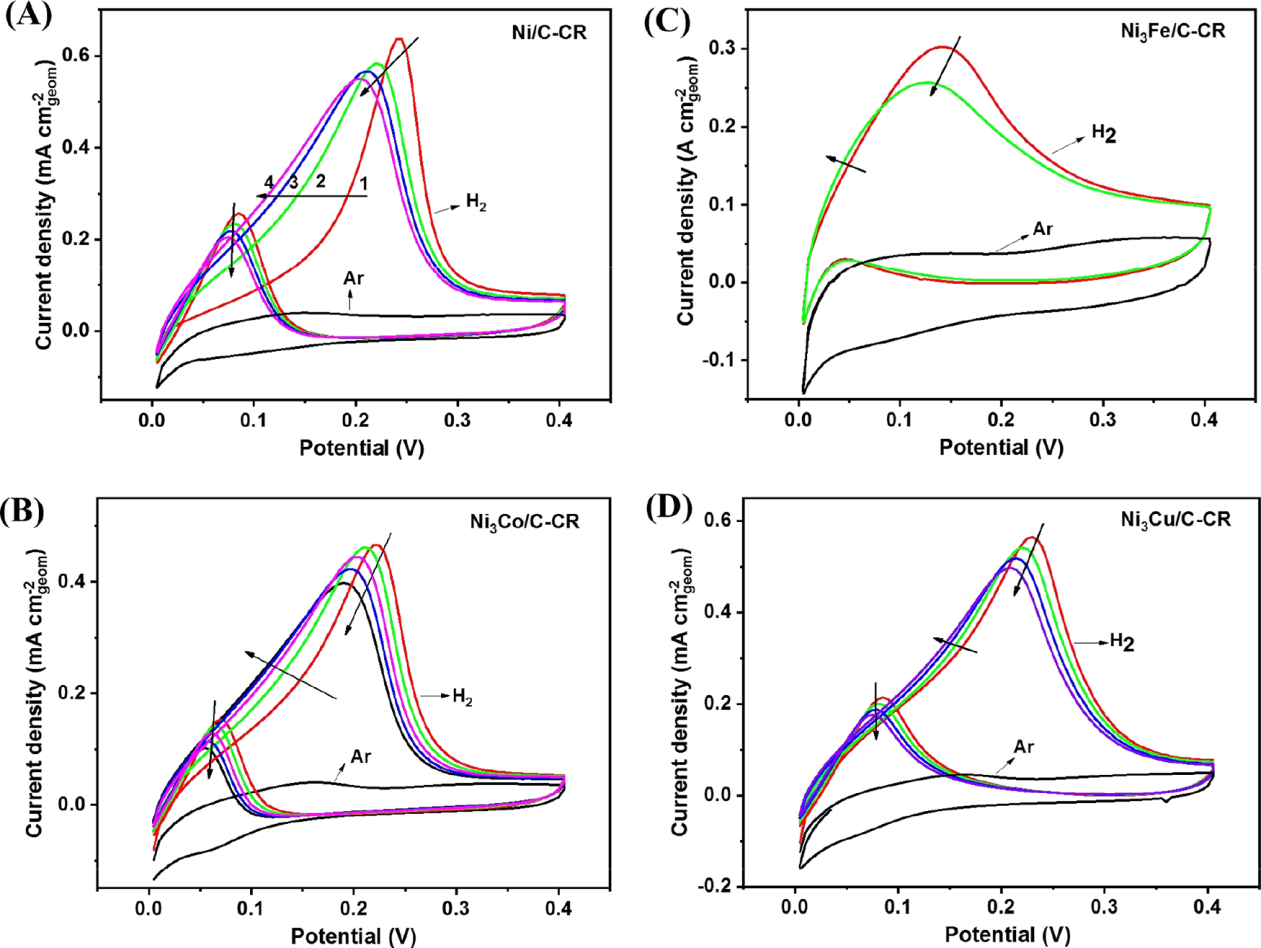
HOR polarization curves and cyclic voltammograms of the catalysts synthesized by chemical reduction. Ar- or H_2_-saturated (as indicated) in 0.1 mol dm^–3^ KOH, 25 °C, rotation rate 1600 rpm, sweep rate 1 mV s^–1^. (A) Ni/C, (B) Ni_3_Co/C, (C) Ni_3_Fe/C, and (C) Ni_3_Cu/C.

**Figure 10:**
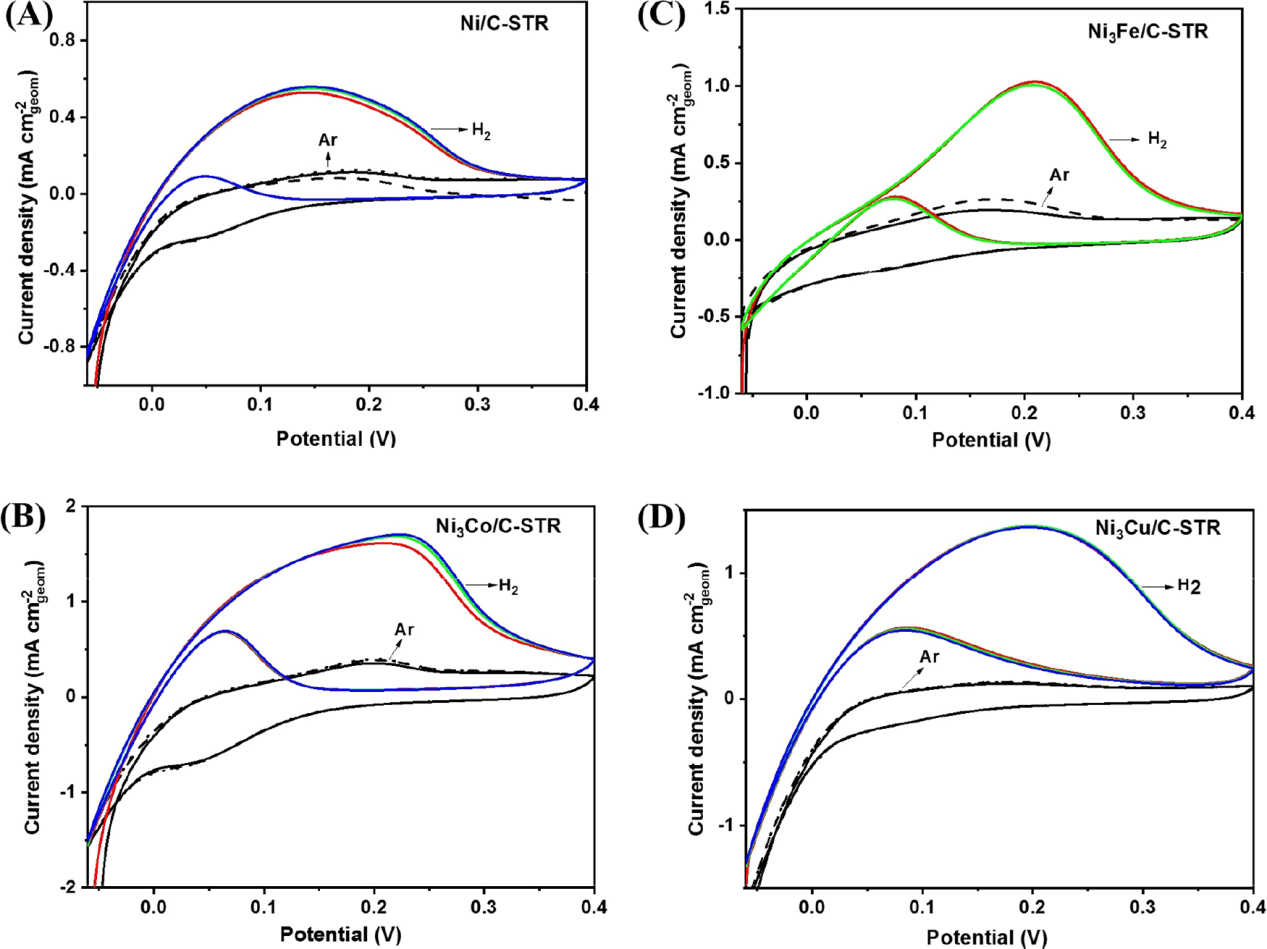
HOR polarization curves and cyclic voltammograms of the catalysts synthesized by solvothermal reduction. Ar- or H_2_-saturated (as indicated) 0.1 mol dm^–3^ KOH, 25 °C, rotation rate 1600 rpm, sweep rate 1 mV s^–1^. (A) Ni/C, (B) Ni_3_Co/C, (C) Ni_3_Fe/C, and (C) Ni_3_Cu/C.

HOR polarization curves with the background CVs in Ar are shown in Figure 9 for the samples synthesized by chemical reduction and in Figure 10 for the samples synthesized by solvothermal reduction. For the latter samples only the CVs after activation are shown. For the samples synthesized by chemical reduction, the CVs in the Ar-purged electrolytes (Figure 9) show two distinguishable peaks at approximately 0.15 V and a little above 0.3 V in the positive-going sweep. For the samples synthesized by solvothermal reduction, in the CVs in the Ar-purged electrolytes (Figure 10) only one peak at approximately 0.2 V is clearly visible; otherwise, the voltammogram is broad and featureless. In the negative-going sweep a peak at 0.05 V, with some variation in the exact potential from sample to sample, is apparent, corresponding to the reduction of Ni(OH)_2_ back to Ni for all samples.

**Figure 11:**
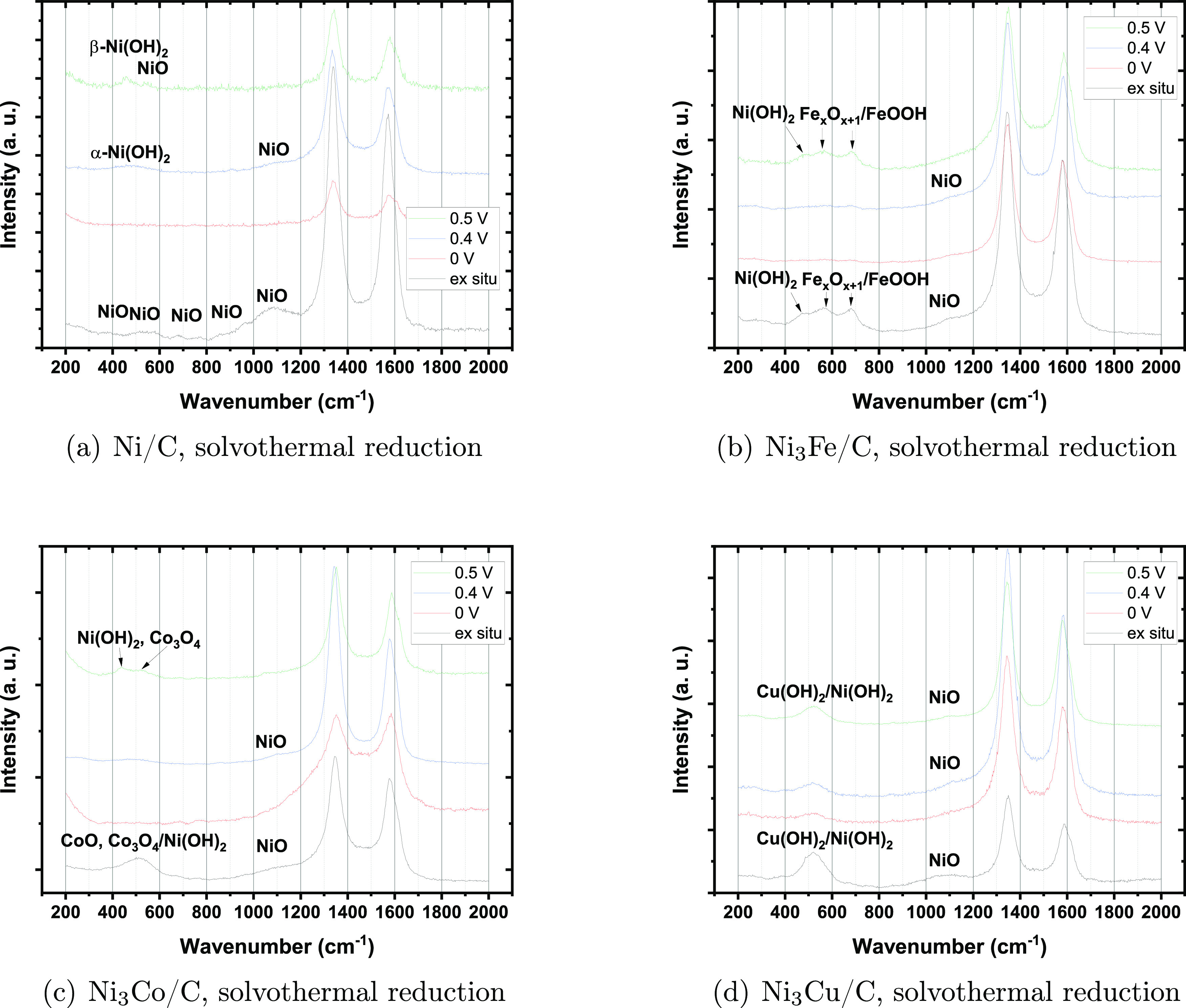
Ex situ and in situ Raman spectra for (a) Ni/C, (b) Ni_3_Fe/C, (c) Ni_3_Co/C, and (d) Ni_3_Cu/C. All samples synthesized by solvothermal reduction. The in situ spectra were collected at 0, 0.4, and 0.5 V in 0.1 mol dm^–3^. The approximate peak positions for NiO, α-Ni(OH)_2_, and β-Ni(OH)_2_ and oxides and hydroxides of the alloying element are indicated by the corresponding labels in the figure. See text for wavenumber values.

Figure 11 shows ex situ Raman spectra for mono- and bimetallic samples synthesized by solvothermal reduction and in situ spectra collected for the same sample after activation. For all catalysts, the D- and G-band peaks of carbon are clearly visible at approximately 1325 and 1580 cm^–1^.^51^

For Ni/C we assign the peaks at lower wavenumbers to NiO, α-Ni(OH)_2_, or β-Ni(OH)_2_. The ex situ spectrum shows clear indications of the presence of NiO, for which peaks are expected at 400, 530, 730, 900, and 1090 cm^–1^. At 0.4 V the spectrum shows features compatible with α-Ni(OH)_2_, for which peaks are expected at 460 and 1637 cm^–1^. While the latter would be masked by the carbon G-peak, the former is clearly visible. A shoulder is discernible at a little less than 1100 cm^–1^ and may be related to NiO. For β-Ni(OH)_2_, peaks at 445 and 518 cm^–1^ are expected. The former is clearly visible in the spectrum collected at 0.5 V, whereas a small feature at approximately 530 cm^–1^ may also be associated with NiO.^52^

For the Ni_3_Fe catalyst the ex situ spectrum shows peaks at 480, 562, and 684 cm^–1^. The peak at 480 cm^–1^ may correspond to Ni(OH)_2_^53^ while the peaks at 562 and 684 cm^–1^ may correspond to Fe_3_O_4_ or FeOOH.^54^ The shoulder at 1100 cm^–1^ corresponds to NiO.^55^ The in situ spectrum at 0.5 V vs RHE is similar to the ex situ spectrum, giving clear indications of oxide/hydroxide species. At 0.4 V vs RHE, the intensity of the peaks corresponding to Ni hydroxide and iron oxide/hydroxide is reduced. A peak corresponding to NiO is clearly visible. At 0 V vs RHE there are still traces of peaks related to oxide/hydroxide species of nickel and iron in the spectrum. For a more detailed interpretation, see ref (38). In the figure we have indicated the approximate peak positions for the various species by the corresponding chemical formulas.

For Ni_3_Co, the broad peak from 400 to 600 cm^–1^ in the spectrum recorded ex situ may correspond to CoO, Co_3_O_4_ , and Ni(OH)_2_,^53,56,57^ while the shoulder at 1100 cm^–1^ corresponds to NiO.^55^ At 0.5 V, the peak at 440 cm^–1^ corresponds to Ni(OH)_2_,^53^ while peaks at 525, 680, and 760 cm^–1^ are associated with Co_3_O_4_.^56,57^ At 0.4 V, the broad band from 400 through 600 cm^–1^ appears with much lower intensity and is associated with CoO, Co_3_O_4_, andNi(OH)_2_.^57^ Again, we associate the peak at 1100 cm^–1^ with NiO.^55^ At 0 V, two small peaks appear at 680 and 760 cm^–1^ and are related to Co_3_O_4_.^56,57^

For Ni_3_Cu, both the Raman spectrum recorded ex situ and those recorded in situ at 0, 0.4, and 0.5 V vs RHE display a broad peak covering the range 430–620 cm^–1^ and may correspond to Cu(OH)_2_^58^ and Ni(OH)_2_.^53^ The broad peak at 270 cm^–1^ corresponds to CuO,^58^ while the peak at 1090 cm^–1^ corresponds to NiO.^55^

In the figure we have indicated the approximate peak positions for the various species by the corresponding chemical formulas. At 0 V, the Raman spectrum for the Ni/C sample is completely featureless apart from the two carbon peaks, and neither the NiO nor any of the Ni(OH)_2_ peaks apparent ex situ are present. This is similar for the Ni_3_Co/C and to some extent also for the Ni_3_Fe/C sample. However, for the Ni_3_Cu/C catalyst, peaks corresponding to oxide-containing species are still present in the Raman spectra at 0 V, although the peak height is substantially reduced. This indicates that the three former catalysts are more or less completely reduced by the activation procedure, whereas the Ni_3_Cu/C catalyst is not.

**Figure 12:**
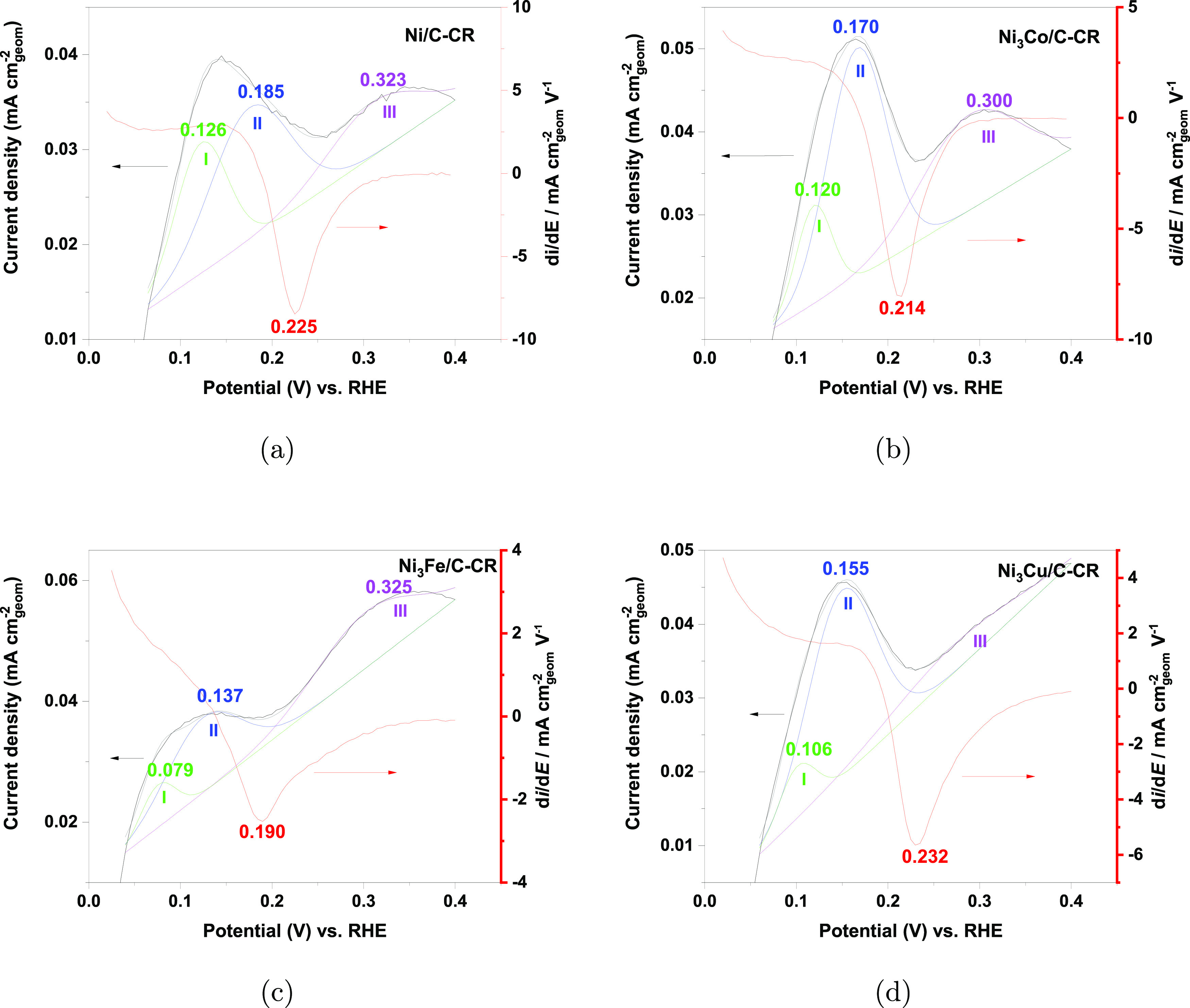
Voltammograms (black curves) in argon-purged solutions (left axis) and a possible set of peaks from which the voltammograms are composed. These include a low-potential peak (I, green curve), an intermediate-potential peak (II, blue curve), and a high-potential peak (III, purple curve). The derivatives of the currents recorded in positive-going sweeps with respect to potential (d*i*/d*E*) in hydrogen-purged (H_2_-saturated) solutions are included (red curve, right axis). The numbers show the peak potentials for the curve in the corresponding color. (a) Ni/C, (b) Ni_3_Co/C, (c) Ni_3_Fe/C, and (d) Ni_3_Cu/C. All samples were synthesized by chemical reduction.

A more detailed analysis of the cyclic voltammograms in the potential range of 0–0.4 V is offered in Figure 12. The figure shows a possible deconvolution of the anodic part of the cyclic voltammograms recorded in argon-purged solutions for samples of Ni/C, Ni_3_Co/C, Ni_3_Cu/C, and Ni_3_Fe/C prepared by chemical reduction. (A similar deconvolution carried out for the STR samples is presented in the Supporting Information.) As illustrated, the CVs can be deconvoluted into three distinctive peaks I, II, and III, listed in the order of increasing peak potential. Figure 12 also includes the derivatives of the HOR current density *i* with respect to the potential *E*, d*i*/d*E*, for the anodic part of the HOR polarization curves in Figure 9 recorded after a stable response had been obtained. The derivatives are positive below approximately 0.15 V. As the potential enters the range 0.15 –0.2 V the derivative becomes negative, displays a sharp minimum, and then increases again and remains approximately constant in the remainder of the potential range.

The potentials at which the minima in d*i*/d*E* appear are shown in Table 3. These potentials vary by less than approximately 40 mV for each series of samples. The potentials are approximately 70 mV lower for samples synthesized by chemical reduction than for those synthesized by solvothermal reduction. However, all the values in Table 3 are in the range expected for passivation of Ni as inferred from its Pourbaix diagram (see the Supporting Information).

**Table 3:** Potential at which d*i*/d*E* is Minimal^a^

	potential/V			
synthesis	Ni	Ni_3_Fe	Ni_3_Co	Ni_3_Cu
CR	0.225	0.190	0.214	0.232
STR	0.276	0.267	0.287	0.300

a Data were taken from Figure 12 and a corresponding figure for the samples synthesized by solvothermal reduction (see the Supporting Information).

**Figure 13:**
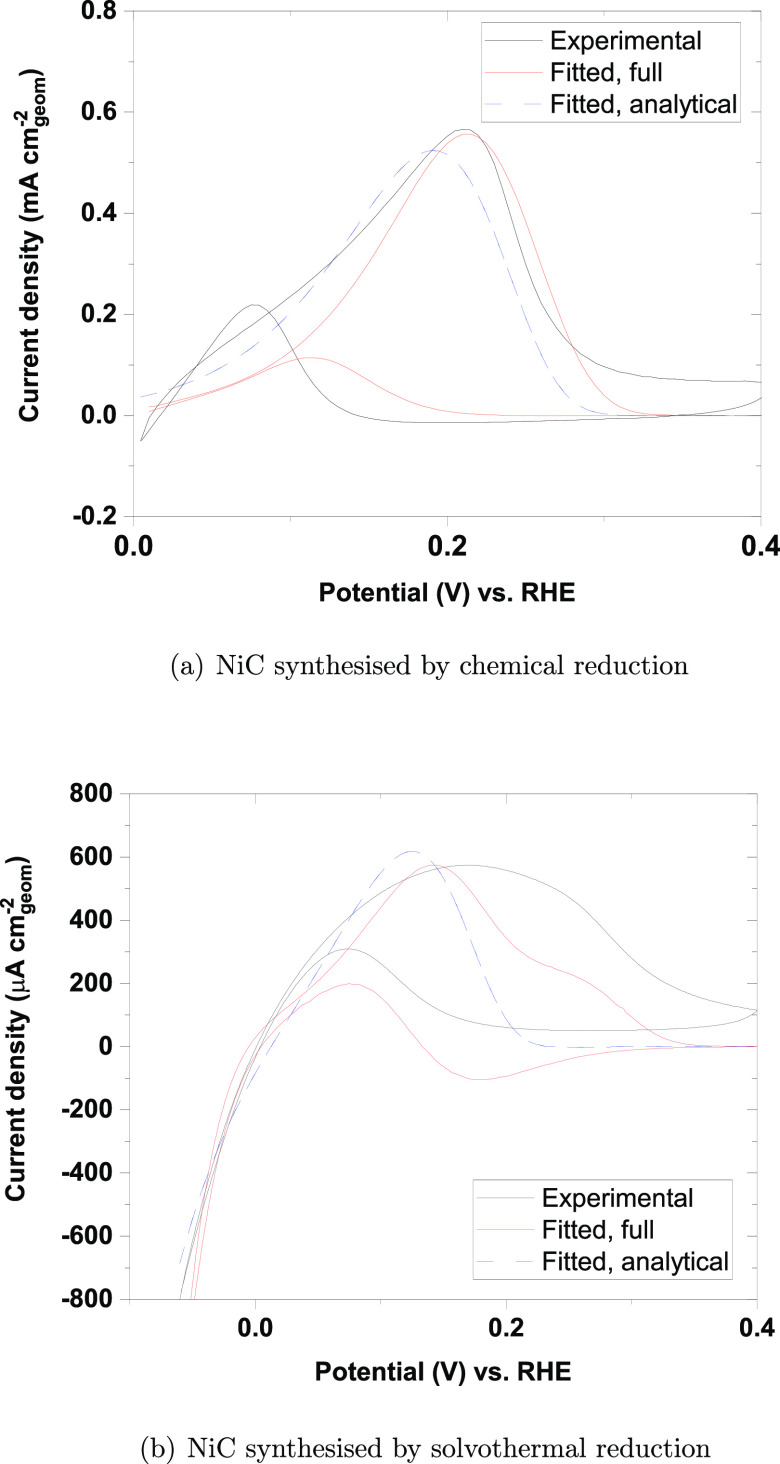
Experimental and simulated results for (a) sample 2 of Ni/C synthesized by chemical reduction and (b) sample 5 of Ni/C synthesized by solvothermal reduction. The simulated results were calculated from the microkinetic model for the reactions in eqs 2–6 (labeled “fitted, full” in the legend); see the Supporting Information for details. The figure also includes simulated results (labeled “fitted, analytical”) calculated from eq 7.

The experimental data in Figures 9 and 10 are compared to the predictions of the microkinetic model proposed in Kabir et al.^15^ in Figure 13. The model assumes the following sequence of steps for the hydrogen-oxidation reaction
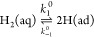
2
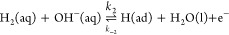
3
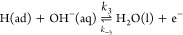
4and also that the reactions
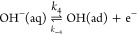
5
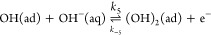
6take place at the Ni surface. The (numerical) solution to the nondimensional rate equations for these steps was fitted to experimental data as explained in the Supporting Information. The model is in reasonable accord with the experimental results and reproduces the forward peak between 0.1 and 0.2 V. The simulated curve in the positive-going scan is narrower than the experimental voltammogram and also displays a hump at approximately 0.25 V. The simulated negative-going scan has an anodic peak centered at approximately 0.08 V.

The voltammograms in hydrogen-containing solutions were also fitted to the simplified equation
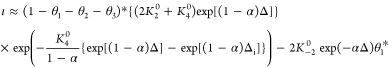
7in which we used α = 0.5 for the symmetry factor, Δ = *FE*/*RT* is a dimensionless potential,  are dimensionless rate constants,  is the fraction of free Ni surface at the lower vertex potential of the voltammogram, and ι = *RTI*/*AF*^2^Γν is the dimensionless current. (Γ is the maximum number of adsorbates per surface area, *R* is the gas constant, *T* is the temperature, *F* is the Faraday constant, *A* is the electrode area, *I* is the current, and ν is the sweep rate.) The main assumption behind eq 7 is that the Tafel reaction in eq 2 proceeds at a negligible rate, that the reactions 3 and 4 proceed at the same rate, that reaction 5 is irreversible, and neglecting reaction 6. The advantage of eq 8 is the substantially reduced number of free parameters (effectively four) compared to the full model resulting in a substantially more efficient fitting. The range of validity of eq 7 and other details are given in the Supporting Information. In spite of its simplicity, this model appears to capture some essential characteristics of the experimental data in the forward scan, Figure 13.

We also derived an equation for the current assuming that the reaction (see the Supporting Information for references)
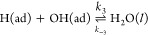
8limits the reaction rate. However, this model did not give good fits to the experimental data. The reader is referred to the Supporting Information for details.

The model results also indicate that the procedure pursued in generating the data in Figure 12 is essentially valid; see the Supporting Information for details.

The small differences in the potentials at which the minima in d*i*/d*E* appear (Table 3) imply that the potential range through which the catalyst surface is in the reduced state and active for the HOR will not be dramatically influenced by doping the Ni catalyst. Since this passivation places a cap on the maximum admissible overpotential, any beneficial effect of the doping of Ni with these elements will have to be primarily through the exchange current density. From the analysis in the Supporting Information it appears that this is best based on the total current in the vicinity of 0 V, since the contribution from the oxidation of the Ni surface will be negligible there.

Tables 4 and 5 summarize and compare the electrochemical parameters such as the exchange current density, ECSA, and mass-normalized activity of the CR and STR catalysts in HOR. For mass normalization of the data with respect to Ni we used the values in column 5 in Table 2.

**Table 4:** Electrochemical Parameters for the Catalysts Prepared by Chemical Reduction

						
sample	ECSA_1_^a^ (cm_Ni_^2^ cm_geom_^–2^)	ECSA_2_^b^ (m_Ni_^2^ g_Ni_^–1^) (% of STEM)	*i*^c^ at 0.05 V (mA )	*i*^d^ at 0.05 V (A g_cat_^–1^)	*i*^e^ at 0.05 V (Ag_Ni_^–1^)	*i*_0_^f^ (μA cm_Ni_^–2^)
Ni/C	2.67	3.09 (4.9)	0.098 ± 0.04	0.4 ± 0.1	1.1 ± 0.5	40 ± 1
Ni_3_Co/C	1.52	1.93 (6.7)	0.09 ± 0.02	0.4 ± 0.1	1.1 ± 0.3	47 ± 7
Ni_3_Cu/C	2.88	4.40 (8.8)	0.10 ± 0.01	0.41 ± 0.03	1.6 ± 0.2	37 ± 7
Ni_3_Fe/C	1.49	1.89 (4.2)	0.16 ± 0.01	0.64 ± 0.04	2.0 ± 0.1	70 ± 20

a ECSA_1_ (): The electrochemical surface area of Ni per unit of the geometric surface area of the RDE.

b ECSA_2_ (): The electrochemical surface area of the catalyst computed from
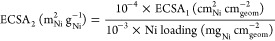
The Ni loading in the above equation was computed by multiplying the catalyst loading of (250 μg cm^–2^) with the weight percents from Table 2. % of TEM: The ratio of the ECSA_2_ from the *S*_TEM_ (Table 1).

c *i* at 0.05 V in mA : The raw electrochemical activity nomalized with respect to the geometric surface area of the RDE at an electrode overpotential of 0.05 V.

d *i* at 0.05 V in A : Catalytic activity normalized with respect to the catalyst loading at an electrode overpotential of 0.05 V computed from
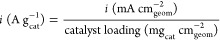

e *i* at 0.05 V in A : Catalytic activity, normalized with respect to the Ni loading at an electrode overpotential of 0.05 V computed from
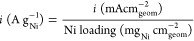
The Ni loading in the above equation was computed by multiplying the catalyst loading (250 μg cm^–2^ for the CR samples) with the weight percents from Table 2.

f *i*_0_ in μA : Exchange current density normalized with respect to the surface area of nickel evaluated from the charge in oxidation peaks for Ni in Ar-purged solutions, assuming a charge density of 514 μC cm^–2^. See the Supporting Information for details.

**Table 5:** Electrochemical Parameters for the Catalysts Prepared by Solvothermal Reduction^a^

						
sample	ECSA_1_ (cm_Ni_^2^ cm_geom_^–2^)	ECSA_2_ (m_Ni_^2^ g_Ni_^–1^) (% of STEM)	*i* at 0.05 V (mA )	*i* at 0.05 V (A)	*i* at 0.05 V (A )	*i*_0_ (μA )
Ni/C	10.38	2.77 (3.3)	0.4 ± 0.1	0.4 ± 0.1	0.8 ± 0.2	40 ± 2
Ni_3_Co/C	24.48	5.13 (16.1)	0.6 ± 0.1	0.5 ± 0.1	1.3 ± 0.3	19.0 ± 0.6
Ni_3_Cu/C	10.62	2.47 (6.4)	0.5 ± 0.1	0.3 ± 0.1	0.9 ± 0.3	42 ± 3
Ni_3_Fe/C	5.07	1.61 (3.2)	0.2 ± 0.01	0.154 ± 0.005	0.4 ± 0.1	7 ± 2

a For an explanation of the terms in the column headings, see the footnotes in Table 4. Due to an effect of electrode rotation, the current densities at 0.05 V are given for one sample only, i.e., samples 5, 4, 3, and 2 for Ni/C, Ni_3_Co/C, Ni_3_Cu/C, and Ni_3_Fe/C, respectively, rather than for the other samples. See the Supporting Information for details.

Comparison of the surface areas from TEM (Table 1) to those evaluated electrochemically, c.f., the ECSA_2_ values in Tables 4 and 5, shows that in both the CR and STR catalysts a mere 4–16% of the expected (based on TEM surface area estimations, Table 1) surface is accessible for the HOR. The Raman spectra show that the cause for this low utilization cannot to a large degree be associated with the formation of oxides at the surface.

The two series of catalysts display quite similar activity for the HOR, ranging from 0.15 to 0.6 A g_cat_^–1^ at 0.05 V. The Ni mass specific activity for the CR catalysts (1.1–2 A g_Ni_^–1^ at 0.05 V, Table 4) is somewhat higher than for the STR samples (0.4–1.3 A g_Ni_^–1^, Table 5). For the CR samples the HOR activity of the Ni_3_Fe samples displays a significantly higher catalytic activity than the other compositions. Ni–Fe electrocatalysts have also been reported to be more active than Ni, Ni–Co, and Ni–Cu catalysts for the HOR.^19,20^ However, for the samples synthesized by the solvothermal method such a trend is not apparent.

The area-normalized activities are all in the range of 40 μA cm^–2^, except for the iron-containing samples that are either somewhat higher (for the CR samples) or lower (for the STR) samples. The area normalization for the latter samples is probably the least reliable of the set, however.

**Figure 14:**
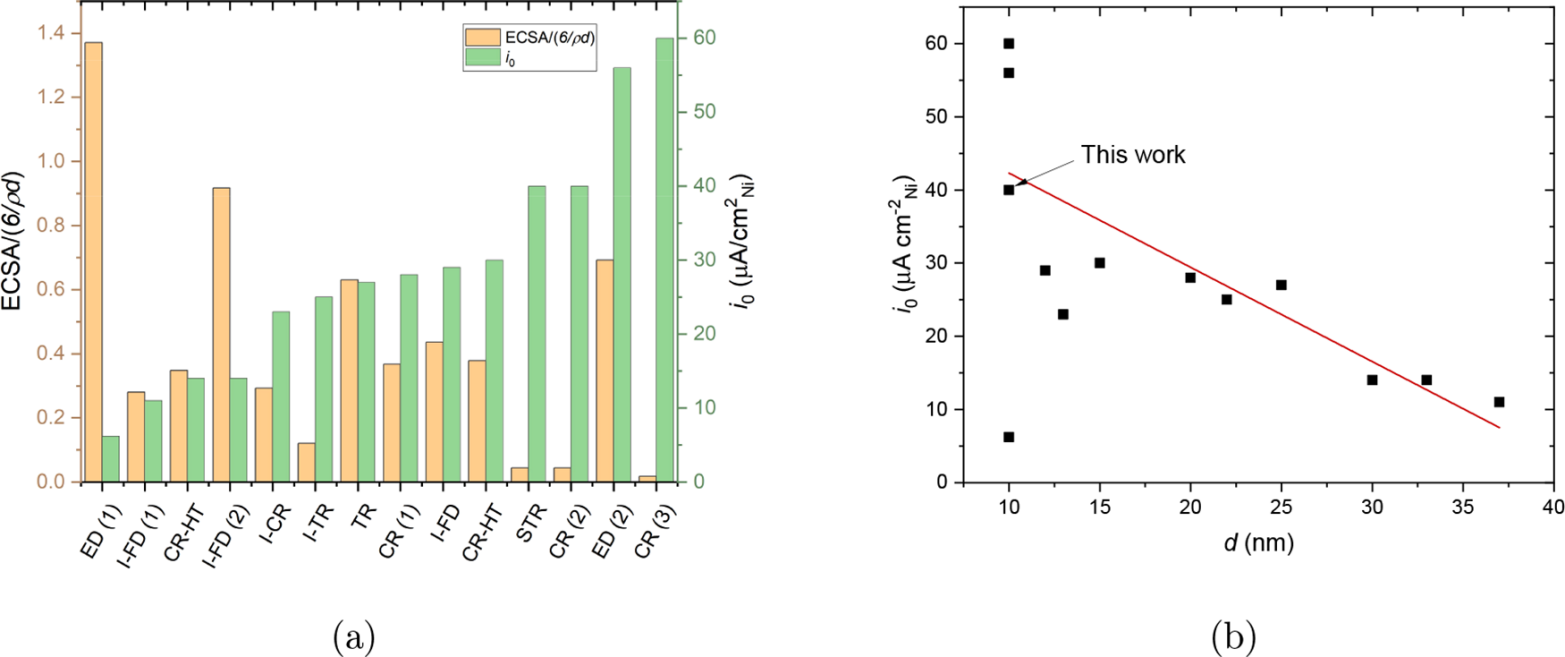
(a) Ratio of the measured ECSA to the single-particle ECSA (ECSA/(6/*ρd*)) and ECSA-normalized exchange current density *i*_0_ for Ni catalysts^60^ synthesized by different methods. Abbreviations: ED (1) = electrodeposited Ni/XC-72 catalysts ;^21^ I-FD (1) = Ni/XC-72 catalysts by impregnation + freeze-drying ;^23^ CR-HT = Ni/XC/72 by chemical reduction + hydrothermal treatment ;^61^ I-FD (2) = Ni_0.95_Cu_0.05_/XC-72, impregnation + freeze-drying ;^23^ I-CR = Ni/KB, impregnation and chemical reduction,^62^ I-TR = Ni_0.95_Cu_0.05_/XC-72, impregnation + thermal reduction ;^2^ TR = Ni_9_Mo_1_/KB, thermal reduction ;^15^ CR (1) = Ni/N-CNT, chemical reduction + hydrothermal treatment ;^29^ I-FD = Ni/KB, impregnation + freeze-drying ;^63^ CR-HT = Ni/BC, Ni/NC, Ni/SC, chemical reduction + hydrothermal treatment ;^61^ STR = Ni/XC-72, solvothermal synthesis (this work); CR(2) = Ni/VX-CMAX22, chemical reduction (this work); ED (2) = Ni-NiO/XC-72, electrodeposition ;^21^ CR (3) = Ni_3_Fe/VX-CMAX22, chemical reduction.^19^ (b) ECSA-normalized exchange current density *i*_0_ vs particle size *d* for the same catalysts as in (a).

Figure 14a compares the ECSA-normalized exchange current densities to literature data. The figure also includes a comparison of the ECSA per Ni mass (this corresponds to ECSA_2_ in Tables 4 and 5). The latter comparison is presented as the ECSA divided by the surface area per gram Ni for a single particle computed from the formula 6/*ρd*. We will refer to the ECSA divided by 6/*ρd* as catalyst utilization below. This formula assumes spherical particles of diameter *d* (see references given) and Ni density ρ.

In an attempt to rationalize the substantial differences in the ECSA-normalized exchange-current densities (*i*_0_), we have plotted *i*_0_ vs particle diameter *d* in Figure 14b for the same catalysts as those in Figure 14a; a dependence of catalytic activity on size is well-known for other catalysts and reactions and has also recently been demonstrated for Ni.^59^ For the particles with *d* > 10 nm, the catalytic activity tends to decrease with increasing particle diameter. For catalysts with *d* ∼ 10 nm, the scatter is substantial. However, for two of the data points at 10 nm (from Oshchepkov et al.^21^), the difference is a consequence of a deliberate and controlled change in the oxidation state. For the catalysts synthesized otherwise and either passivated or reduced in situ, the ECSA-normalized exchange-current density decreases with increasing particle diameter. The ESCA-normalized exchange-current density for the catalysts reported in this work is consistent with this trend, Figure 14b.

As Figure 14a shows, there is substantial variation in the ratio of the measured ECSA to the single-particle ECSA. For the majority of results given in Figure 14, the ESCA was evaluated from the α-Ni(OH)_2_ peak at passivated or electrodeposited samples. While the Raman results presented here (Figure 11) provide a basis for the normalization for our Ni/C catalysts, this basis is less directly accessible in the balance of results in Figure 14. If the catalyst surface contains oxides or hydroxides that do not contribute to the charge from which the ESCA is evaluated but do participate actively in the HOR, the ESCA would be substantially underestimated if evaluated from the α-Ni(OH)_2_ peak. Also, for our Ni_3_Co, Ni_3_Fe, and Ni_3_Cu, the Raman results show that other oxides will contribute substantially to the charge compared to that which for a pure Ni sample would be associated with the α-Ni(OH)_2_ peak. How to normalize such data with respect to the ECSA is less clear-cut than for the Ni/C. Finally, other factors not directly related to the catalysts may compound the assessment, such as the influence of procedures for preparing inks for measurements at rotating-disk electrodes, ionomer–catalyst interaction, and loading.

For the samples investigated in this work, the results from the in situ Raman spectroscopy instill some confidence in the ECSA-normalized values. The low values for the ratio ECSA/(6/*ρd*), on the other hand, are difficult to rationalize unless related to aspects of electrode preparation.

To summarize the electrochemical characteristics of the CR and STR catalysts:

1. Electrochemical preactivation of the CR catalysts via potential cycling within 0–0.4 V has negative a effect on the catalytic activity. On the other hand, a similar preconditioning for the STR catalysts in the potential range −0.2 to 0.4 V was necessary to make the nanoparticles catalytically active.
2. In situ Raman spectroscopy for the samples synthesized by solvothermal reduction indicates that after the activation the surface is fully reduced at 0 V, with an exception for Ni_3_Cu/C. As expected, α-Ni(OH)_2_ and β-Ni(OH)_2_ appear at 0.4 and 0.5 V vs RHE, respectively.
3. The absence of any oxides or hydroxides in the Ni/C samples at 0 V investigated by in situ Raman spectroscopy provides a basis for employing the α-Ni(OH)_2_ peak for evaluation of the ESCA.
4. The ECSA-normalized exchange currents and the ECSAs (for the same loading) are quite similar for catalysts made by the two synthesis methods in this work and are in line with literature data indicating a decreasing catalytic activity with increasing particle size.
5. The ECSA is less than 10% of that expected from the particle size, indicating a low catalyst utilization at the rotating disc electrode.
6. The voltammograms for all the catalysts are consistent with a model assuming that the HOR ceases upon the formation of adsorbed OH. The latter is also directly observed through in situ Raman data. The HOR is most likely limited by the Heyrovsky step, eq 3.

Since it appears from the results presented here that the metal composition of Ni-based nanocatalysts is not critical whereas literature results^64−66^ indicate that a way forward would be to stabilize an optimum oxidation state. Several recent contributions have reported encapsulation of Ni, Fe, and Co with carbon shells.^67−69^ The carbon encapsulation has been reported to prevent or limit the oxidation of the catalysts when exposed to air, although in some cases partial oxidation has still been observed.^68,69^

## Conclusions

Supported Ni-based catalysts prepared by the synthesis methods of chemical reduction (CR) and solvothermal reduction (STR) both result in polycrystalline nanoparticles (average 10–15 nm diameter) of near-spherical shape, with narrower particle size distribution for STR. Chemical reduction forms poorly crystalline materials, whereas solvothermal reduction results in an order of magnitude larger crystallites. Solvothermal reduction results in the formation of Ni–M alloys, while chemical reduction gives a mechanical mixture. The catalysts consist of metallic Ni and/or Ni–M alloy cores covered by Ni oxides/hydroxides/oxyhydroxides. The binary catalysts also contain oxides of Cu, Co, or Fe.

In the binary catalysts, Ni atoms tend to segregate on the surface, especially in the catalysts synthesized by solvothermal reduction. Therefore, for some of the catalysts one could a priori expect the electrochemical parameters to be similar to those of monometallic Ni/C, which was a posteriori proven experimentally. In other respects the surfaces emerging from the synthesis differ significantly for the two synthesis methods. Pristine solvothermally synthesized samples are practically inactive, and extensive potential cycling in the range −0.2  through 0.4 V is necessary in order to activate them for the HOR. Similar activation procedures had a negative effect on the samples synthesized by chemical reduction.

As shown by Raman spectroscopy the activation procedure for the STR samples removes the oxides and hydroxides, leaving the catalyst surface completely reduced at 0 V vs RHE; α-Ni(OH)_2_ has formed at 0.4 V. Since the β-Ni(OH)_2_ hydroxide was not observed in the potential region for the activation procedure, this provides a basis for the normalization of the Ni/C samples from the charge associated with the α-Ni(OH)_2_ peak in voltammograms. The Ni_3_Fe/C and Ni_3_Co/C samples were also fully reduced at 0 V, but the NiCu/C was not. However, for the binary catalysts other oxidation processes than the reaction Ni + 2OH^–^(aq) → α-Ni(OH)_2_ + 2e^–^ will contribute to the charge, rendering the determination of the ECSA somewhat less certain for these compositions.

The monometallic Ni/C-CR and Ni/C-STR catalysts display similar values for exchange current density (40 μA ) and similar mass activities, in the order of 1 A  at 0.05 V vs RHE. Similar values were obtained for the binary catalysts as evaluated by the same procedures. Thus, the HOR catalytic activity is not strongly dependent on the degree of alloying or phase crystallinity. The ECSA-normalized exchange current densities for the samples synthesized in this work are relatively high, which we associate with the small particle size.

Another important parameter determining the catalytic activity for the HOR for these Ni-based catalysts is the potential at which electrochemically reversible hydroxides such as α-Ni(OH)_2_ form at the surface. When fully covered by α-Ni(OH)_2_ the surface becomes inactive for the HOR. Doping the catalysts may result in small variations in this potential with composition, which was observed to range from 190 mV for Ni_3_Fe to 232 mV for Ni_3_Cu/C for the samples prepared by chemical reduction.

## Supporting Information Available

TEM images of Ni/C, Ni_3_Co/C, and Ni_3_Cu/C catalysts; energy-dispersive X-ray spectroscopy; XRD for solvothermally synthesized samples as a function of annealing and ultrasonication; hydrogen temperature-programmed desorption; X-ray photoelectron spectroscopy; activation of electrodes; deconvolution of voltammograms; evaluation of surface area and exchange current density; microkinetic modeling (PDF)
