# Supplementary material for: Effect of the Synthetic Method on the Properties of Ni-Based Hydrogen Oxidation Catalysts
Source: ACS Appl Energy Mater. 2021 Apr 1;4(4):3404–23. doi: 10.1021/acsaem.0c03157 (PMC8153405; doi:10.1021/acsaem.0c03157)
Supplement: Supplementary file 1 — ae0c03157_si_001.pdf [file ae0c03157_si_001.pdf]

Supporting Information for "Effect of the synthetic method on the properties of Ni-based hydrogen oxidation catalysts"[†]

Elena S. Davydova,[‡] Maidhily Manikandan,[¶] Dario R. Dekel,[*,§] and Svein Sunde[*,¶]

‡*The Wolfson Department of Chemical Engineering, Technion — Israel Institute of Technology Haifa 3200003, Israel*

¶*Department of Materials Science and Engineering, Norwegian University of Science and Technology (NTNU), NO-7491 Trondheim, Norway*

§*The Wolfson Department of Chemical Engineering, Technion — Israel Institute of Technology Haifa 3200003, Israel and The Nancy & Stephen Grand Technion Energy Program (GTEP), Technion — Israel Institute of Technology, Haifa 3200003, Israel*

E-mail: dario@technion.ac.il; svein.sunde@ntnu.no

Phone: +47 73 59 40 51. Fax: +47 73 59 11 05

List of Figures

S1 (a,b) Bright field TEM images of the Ni/C made by chemical reduction with two different magnifications; (c) Particle size distribution for the Ni/C made by chemical reduction; (d,e) Bright field TEM images of the Ni/C catalyst made by solvothermal reduction with two different magnifications(f) Particle size distribution for Ni/C catalyst made by solvothermal reduction. S9

[†]Both authors Davydova and Manikandan contributed equally to this work.

S2 (a,b) Bright field TEM images of the Ni_3Co/C made by chemical reduction with two different magnifications; (c) Particle size distribution for the Ni_3Co/C made by chemical reduction; (d,e) Bright field TEM images of the Ni_3Co/C catalyst made by solvothermal reduction with two different magnifications(f) Particle size distribution for Ni_3Co/C catalyst made by solvothermal reduction. S10

S3 (a,b) Bright field TEM images of the Ni_3Cu/C made by chemical reduction with two different magnifications; (c) Particle size distribution for the Ni_3Cu/C made by chemical reduction; (d,e) Bright field TEM images of the Ni_3Cu/C catalyst made by solvothermal reduction with two different magnifications(f) Particle size distribution for Ni_3Cu/C catalyst made by solvothermal reduction. S11

S4 TEM particle size histogram for the as prepared samples of catalyst made by solvothermal reduction. (a) Ni/C (b) Ni_3Co/C (c) Ni_3Cu/C (d)Ni_3Fe/C . . . S12

S5 STEM-EDS (Ultra Zeiss). Ni mapping. Scale bar 200 nm. Identical points are marked in red. The sample was synthesised by chemical reduction. . . . S13

S6 STEM-EDS (Ultra Zeiss). Element mapping for the Ni_3Cu/C-CR sample. Scale bar 100 nm. Identical points are marked in red. Green — Ni, red — Cu, yellow — overlapping Ni and Cu. S14

S7 STEM-EDS (Ultra Zeiss). Element mapping for the Ni_3Co/C-CR sample. Scale bar 100 nm. Identical points are marked in red. Green — Ni, red — Fe, yellow — overlapping Ni and Co. S15

S8 STEM-EDS (Ultra Zeiss). Element mapping for the Ni_3Fe/C-CR sample. Scale bar 100 nm. Identical points are marked in red. Green — Ni, red — Co, yellow — overlapping Ni and Co. S16

S9 X-ray diffractograms for pristine samples of Ni/C, Ni_3Co/C, Ni_3Cu/C, Ni_3Fe/C synthesised by solvothermal reduction, and carbon (Vulcan XC-72). S17

S10 X-ray diffractograms for a sample of Ni/C synthesised by solvothermal reduction with a reduced amount of TOP after annealing at 500 °C. The Ni:TOP ratio was 1:2 for this synthesis. S18
S11 Mass change upon heating the samples of Ni/C, Ni_3Co/C, Ni_3Cu/C, Ni_3Fe/C in air at a rate of 10 °C $^{-1}$min (TGA). The samples were synthesised by solvothermal reduction. S19
S12 X-ray diffractograms for samples of Ni/C, Ni_3Co/C, Ni_3Cu/C, and Ni_3Fe/C after annealing at 500 °C and removal of the carbon support in TGA. The peak labelled (200) here corresponds to (111) in the previous figure. The samples were synthesised by solvothermal reduction. S19
S13 Deconvolution of the H_2-TPR spectra for the catalysts made by chemical reduction. S20
S14 Deconvolution of the H_2-TPR spectra for the catalyst made by solvothermal reduction. S21
S15 Deconvolution of HR-XPS spectra for the Fe 2$p_{3/2}$ line of Ni_3Fe/C (a) and (b) for the Cu 2p3/2 line of Ni_3Cu/C both made by chemical reduction. S25
S16 X-ray photoelectron spectra (XPS) of the solvothermally synthesised samples; 2p region from (a) Ni_3Fe/C, (b) Ni_3Co/C and (c) Ni_3Cu/C. S27
S17 X-ray photoelectron spectra (XPS) for samples of Ni/C, Ni_3Co/C, Ni_3Cu/C, and Ni_3Fe/C synthesised by solvothermal reduction. S29
S18 HOR polarisation curves on the as-prepared Ni_3Fe/C-CR catalyst (black) and on the same electrode after the electrochemical pre-activation procedure. H_2-purged 0.1 mol dm^{-3} KOH, 25 °C, sweep rate 1 mV $^{-1}$s, rotation rate 1600 rpm, catalyst loading 400 μg cm$^{-2}{}_{\text{geom}}$. The arrows show the direction of the potential sweeps. S32

S19 HOR polarisation curves on the as-prepared Ni_3Fe/C-STR catalyst (black) and on the same electrode after the electrochemical pre-activation procedure. H_2-purged 0.1 mol dm^{-3} KOH, 25 °C, sweep rate 1 mV $^{-1}$s, rotation rate 1600 rpm, catalyst loading 505 µg cm$^{-2}{}_{geom}$. S33
S20 (a) Current vs. potential for a polycrystalline Pt electrode in a hydrogen-saturated solution of 01. mol dm^{-3} at the angular velocities ω given in the legend. The sweep rate was 5 mV $^{-1}$s. (b) Plot of currents in (a) at 0.385 V vs. $\omega^{1/2}$. S35
S21 Cyclic voltammogram for Ni_3Fe/C (activated electrode, catalyst prepared by solvothermal reduction, data set from Figure S19) with current vs. potential for a Pt electrode included (data set for 400 rpm from Figure S20). S36
S22 Deconvolution of the forward (anodic) sweep of the CVs (black curves) for (a) Ni/C, (b) Ni_3Fe/C, (c) Ni_3Co/C, and (d) Ni_3Cu/C synthesised by chemical reduction (left axis). In the deconvolution presented the CV has been attributed to a contribution from a low-potential process (I, green curve), an intermediate-potential process (II, blue curve), and a a high-potential process (III, purple curve). The derivative di/dE (red curve, right axis) of the HOR polarisation curve obtained from the forward sweeps of the stable-response HOR polarisation. A similar set of deconvolution of the forward (anodic) sweep of the CVs in argon and the derivative di/dE in hydrogen is shown in (e) for Ni/C, (f) for Ni_3Fe/C, (g) for Ni_3Co/C, and (h) for Ni_3Cu/C synthesised by solvothermal reduction. S38
S23 Comparison of cyclic voltammograms with rotation (1600 rpm) (a) and without rotation (b) in argon- and hydrogen-purged solutions as indicated. The sweep rate was 1 mV $^{-1}$s, and the sample was a Ni catalyst prepared by solvothermal reduction and supported on carbon. (First (solid lines) and second cycles (dashed lines) are shown.) S39

S24 Comparison of cyclic voltammograms for Pt on carbon supported in argon-purged solutions with rotation (1600 rpm) and without with a lower vertex potential of −0.06 V (a) and with a lower vertex potential equal to 0 V (b). The sweep rate was 1 mV $^{-1}$s. The solid lines represent the first cycle and the dashed the second. (For the argon-purged solution the first and second cycles more or less overlap.) S41

S25 (a) The positive part of cyclic voltammograms (in A) for five different samples of Ni on carbon (Ni/C) in argon. Samples 1 (black curve), 2 (red curve), and 3 (blue curve) were recorded without rotation. Samples 4 (green curve) and 5 (black dashed curve) were recorded with rotation. The sweep rate was 1 mV $^{-1}$s. For samples 1 through 4 the lower and upper inflection points were selected as 0.14 V and 0.27 V, respectively. For sample 5 they were 0.17 V and 0.28 V. (b) The voltammetic peak after subtraction of a straight baseline for the same samples. (c) The corresponding micropolarisation curves for the same samples. (d) The full cyclic voltammograms (current density) for the same samples in A cm^{-2} based on the geometric electrode area. S42

S26 Approximate solution Eq. (S55) for the HOR if its rate is controlled by the Heyrovsky step reaction (S3) and the Tafel step proceeds at a negligible rate. The various contributions to the overall reaction rate are given as indicated in the figure. The red curve corresponds to the anodic part of the hydrogen reaction and the green curve to its cathodic term as they would appear on an electrode with no formation of surface hydroxides. The term $\exp\left(-\frac{K_4^0}{1-\alpha}\left\{\exp\left[(1-\alpha)\Delta\right]-\exp\left[(1-\alpha)\Delta_\mathrm{i}\right]\right\}\right)$ represents the diminishing available area for the HOR due to reaction (S5). S50

S27 (a). Experimental and simulated results for sample 2 of Ni/C synthesised by chemical reduction. The simulated results were calculated from Eqs. (S17) through (S48). (b). Experimental and simulated results for sample 2 of Ni/C synthesised by chemical reduction. The simulated results were calculated from Eq. (S55). S59
S28 Dimensionless reaction rates with the parameters for the fit in Figure S27. . S60
S29 Pourbaix diagram for Ni. Solid lines represent the diagram for $a_{\mathrm{Ni}} = 1$, and dashed line the diagram for a molality of Ni equal to $m_{\mathrm{Ni}} = 10^{-6}$ mol k^{-1}g H_2O. (a) Complete diagram and (b) expanded view of the hydrogen region at high pH. The blue lines show the electrode potentials for the hydrogen and oxygen reactions. The diagrams were calculated by the EpH package in the HSC Chemistry 9 program, Ver. 9.2.3 by Outotec (`http://www.outotec.com/`).S61
S30 (a). Experimental and simulated results for sample 5 of Ni/C synthesised by the solvothermal reduction. The simulated results were calculated from Eqs. (S17) through (S48). (b). Experimental and simulated results for sample 5 of Ni/C synthesised by solvothremal reduction. The simulated results were calculated from Eq. (S55). S64
S31 Simulated dimensionless reaction rates for reactions (S3) through (S6) for the voltammogram in Figure S30. . . . . S65
S32 Experimental and simulated results for sample 5 of Ni/C synthesised by the solvothermal reduction but with a different set of initial parameters than in Figure S30. The simulated results were calculated from Eqs. (S17) through (S48)..S66
S33 Dimensionless reaction rates with the parameters for the fit in Figure S32. . S67

S34 Experimental and simulated results for Ni/C synthesised by solvothermal reduction. The simulated results were calculated from the analytical, approximate solutions to the microkinetic model for the reactions in Eqs. (S2) through (S6). (a) Heyrovsky-Volmer mechanism, Eq. (S3) *rds.* (b) Heyrovsky-Volmer mechanism, Eq. (S4) *rds.* (c) Bifunctional mechanism, Eq. (S7) *rds.* . S68
S35 Experimental and simulated res ults for Ni_3Co/C synthesised by solvothermal reduction. The simulated results were calculated from the analytical, approximate solution to the microkinetic model for the Heyrovsky-Volmer mechanism, Eq. (S3) *rds*, Eqs. (S2) through (S6) assuming Eq. (S3) to represent the slow step. S69
S36 Simulated dimensionless reaction rates for reactions (S5) (V_4) and (S6) (V_5) in Ar- and H_2-purged solutions vs. dimensionless potential simulated with parameters corresponding to samples synthesised by chemical reduction. The red and blue solid lines are the rates in hydrogen-containing solutions, and the dashed lines are the rates in argon-purged solutions. The solid black line is the simulated curve for the derivative of the dimensionless current with respect to the dimensionless potential, $d\iota/d\Delta$, corresponding to di/dE. . . . S70

List of Tables

S1 H_2 consumption during the H_2-TPR measurements for the catalysts made by chemical reduction. S22
S2 H_2 consumption of the catalyst made by solvothermal reduction during the H_2-TPR measurements. S23
S3 XPS spectral fitting parameters of the Cu $2p_{3/2}$ line for the Ni_3Cu/C catalysts made by chemical reduction. S24

S4 XPS spectral fitting parameters of the Fe $2p_{3/2}$ line for the Ni_3Fe/C catalysts made by chemical reduction. S24
S5 XPS spectral fitting parameters of Ni/C catalysts made by chemical reduction, Ni_3Fe/C,Ni_3Co/C and Ni_3Cu/C: binding energy(eV), % of the metallic Ni, atomic ratio and chemical state. S26
S6 XPS spectral fitting parameters of Ni/C catalyst made by solvothermal reduction, Ni_3Co/C, Ni_3Cu/C and Ni_3Fe/C: binding energy (in eV),% atomic ratios, FWHM (in eV) values and chemical states. S28
S7 Atomic composition of the catalysts characterised by the XPS and EDS methods. The last rightmost column gives the boron content for the CR samples and phosphorous for the STR samples. S31

TEM images of Ni/C, Ni_3Co/C, and Ni_3Cu/C catalysts

Figure S1, S2, and S3 compares TEM images and size distributions of Ni/C, Ni_3Co/C, and Ni_3Cu/C catalysts made by chemical reduction and annealed samples prepared by solvothermal reduction. For Ni/C the average particle size is comparable for the two catalysts and centers around 8 nm. All the samples synthesised by chemical reduction show rather wide particle size distributions as commented in the main text. The distributions for the catalyst made by solvothermal reduction appear a little narrower than for the chemically manufactured catalyst. A slightly smaller mean diameter is observed for the Ni/C catalysts (Figure S1) than for Ni_3Fe/C, Ni_3Co/C (Figure S2) Ni_3Cu/C, and Ni_3Fe/C (Figure S3). However, all in all the series of catalyst appear to be similar in terms of dispersion on the carbon and particle size.

Figure S4 shows particle size distributions for pristine samples of Ni/C, Ni_3Co/C, and Ni_3Cu/C catalysts made by solvothermal reduction, i.e prior to thermal treatment at 500 °C. The thermal annealing thus appears to induce a slight increase in the mean particle size for

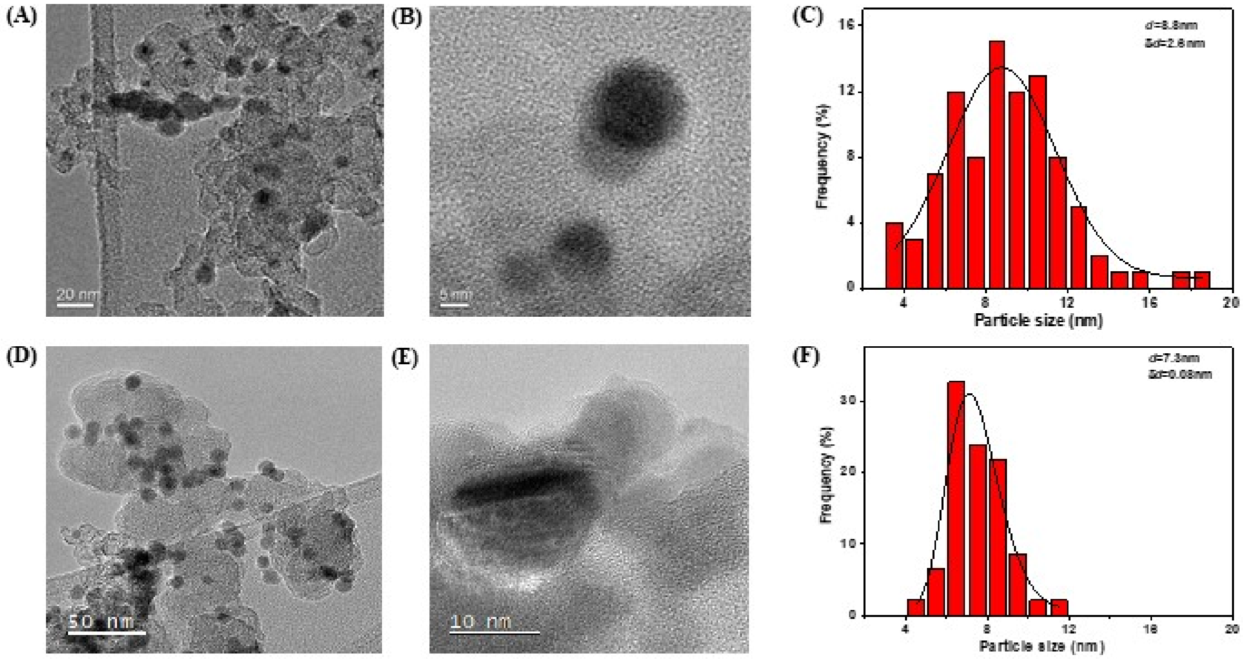

Figure S1: (a,b) Bright field TEM images of the Ni/C made by chemical reduction with two different magnifications; (c) Particle size distribution for the Ni/C made by chemical reduction; (d,e) Bright field TEM images of the Ni/C catalyst made by solvothermal reduction with two different magnifications(f) Particle size distribution for Ni/C catalyst made by solvothermal reduction.

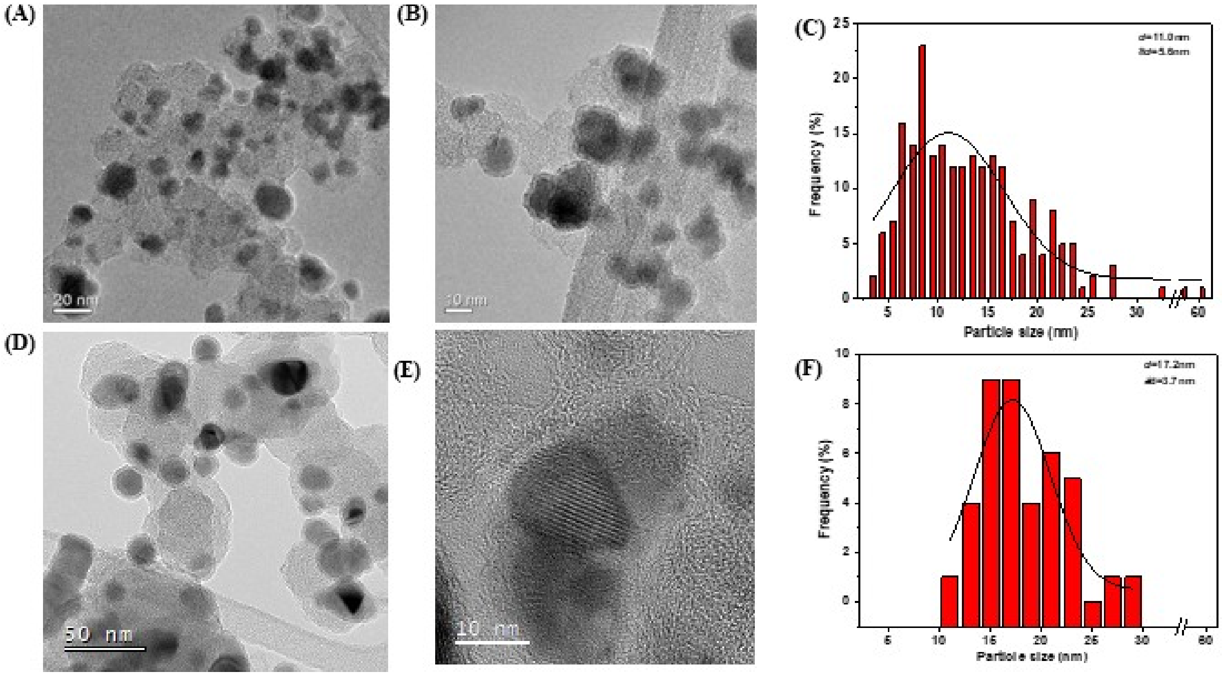

Figure S2: (a,b) Bright field TEM images of the Ni_3Co/C made by chemical reduction with two different magnifications; (c) Particle size distribution for the Ni_3Co/C made by chemical reduction; (d,e) Bright field TEM images of the Ni_3Co/C catalyst made by solvothermal reduction with two different magnifications(f) Particle size distribution for Ni_3Co/C catalyst made by solvothermal reduction.

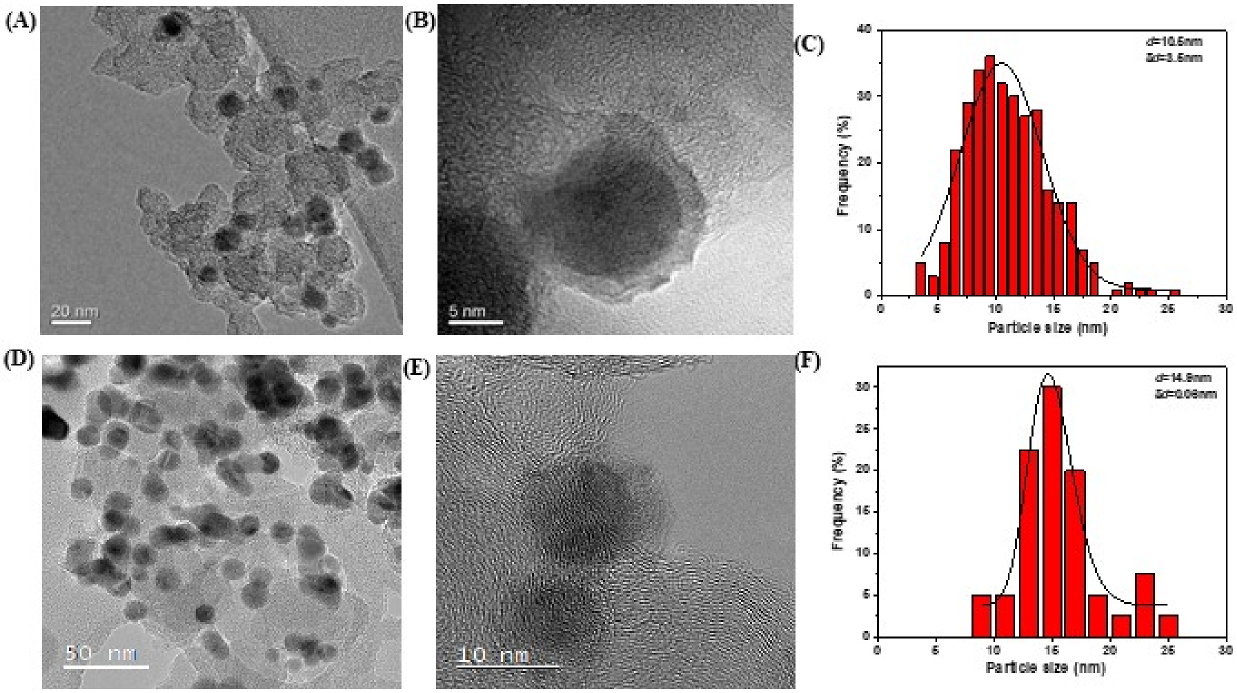

Figure S3: (a,b) Bright field TEM images of the Ni_3Cu/C made by chemical reduction with two different magnifications; (c) Particle size distribution for the Ni_3Cu/C made by chemical reduction; (d,e) Bright field TEM images of the Ni_3Cu/C catalyst made by solvothermal reduction with two different magnifications(f) Particle size distribution for Ni_3Cu/C catalyst made by solvothermal reduction.

Ni/C. The increase is somewhat larger for Ni_3Co/C, Ni_3Cu/C, and Ni_3Fe/C than for the Ni/C sample, compare Figure S4 with Figures S1, S2, and S3 and Figure 3 in the main article.

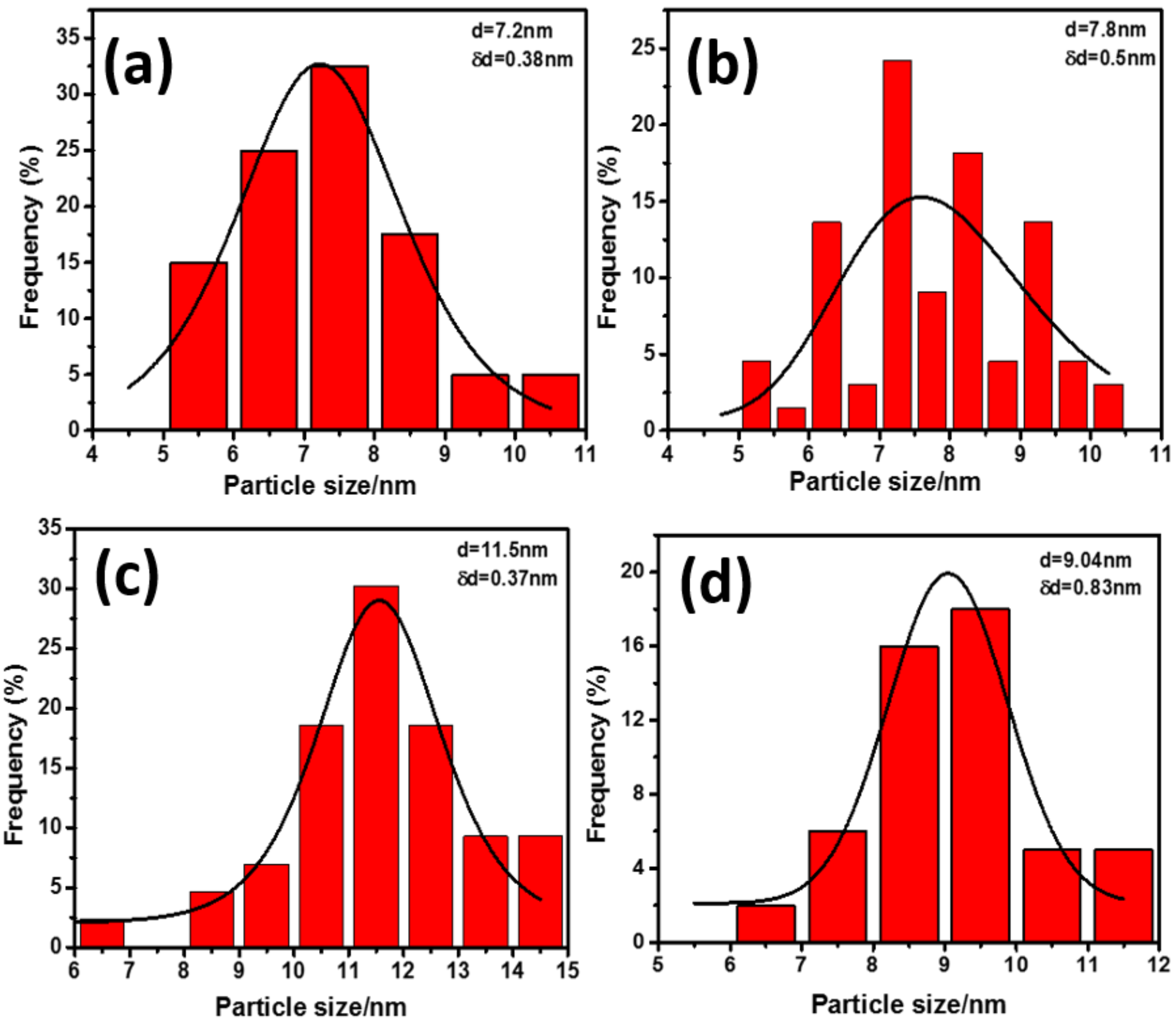

Figure S4: TEM particle size histogram for the as prepared samples of catalyst made by solvothermal reduction. (a) Ni/C (b) Ni_3Co/C (c) Ni_3Cu/C (d)Ni_3Fe/C

Energy dispersive x-ray spectroscopy

Figures S5 through S8 show elemental maps obtained by energy dispersive S(T)EM x-ray spectroscopy (EDS) for samples produced by chemical reduction. Some degree of phase separation is apparent for the Ni_3Co/C, Ni_3Cu/C, and Ni_3Fe/C samples.

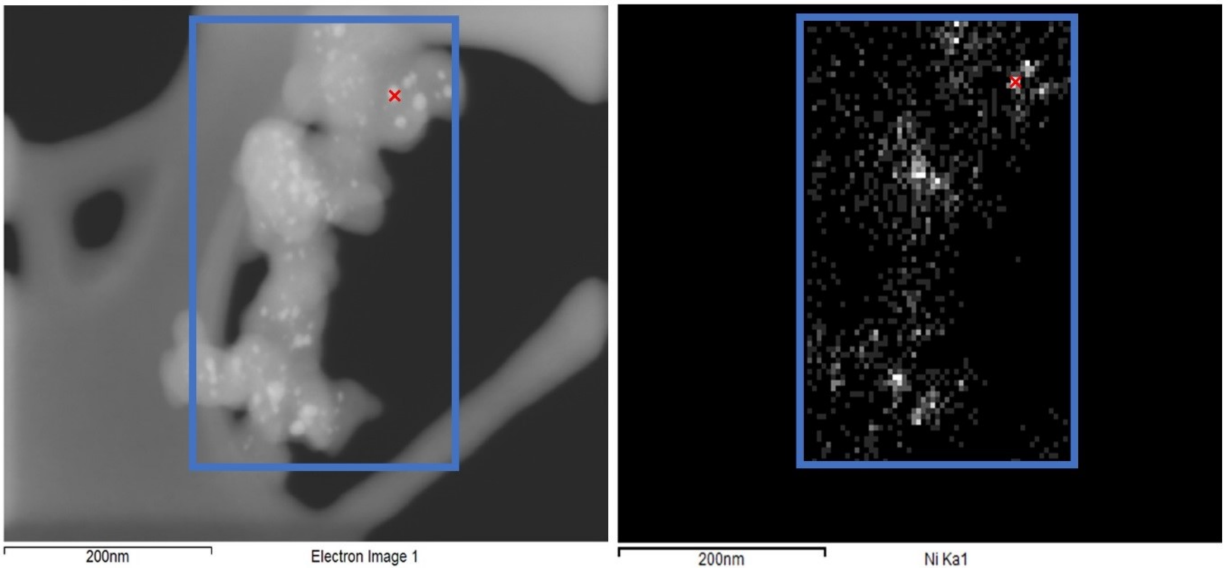

Figure S5: STEM-EDS (Ultra Zeiss). Ni mapping. Scale bar 200 nm. Identical points are marked in red. The sample was synthesised by chemical reduction.

XRD for solvothermally synthesised samples as a function of annealing and ultrasonication

Figure S9 shows the XRD reflections for Ni/C, Ni_3Co/C, Ni_3Cu/C, Ni_3Fe/C, and the carbon support (Vulcan XC-72). The (111) peak for Ni at $2\theta = 44.5°$ is clearly visible for all samples. After annealing, this peak was retained in all samples but the pure nickel sample, Ni/C. After annealing the Ni/C (111) peak had split into two peaks, one at a lower value of 2θ ($2\theta \approx 42°$) and one at a higher 2θ ($2\theta \approx 47°$). Both these peaks are discernible, although almost to a negligible extent in Ni_3Co/C, Ni_3Cu/C, and Ni_3Fe/C, in all samples after annealing.

Reducing the amount of TOP in the synthesis restored the peaks to those expected for

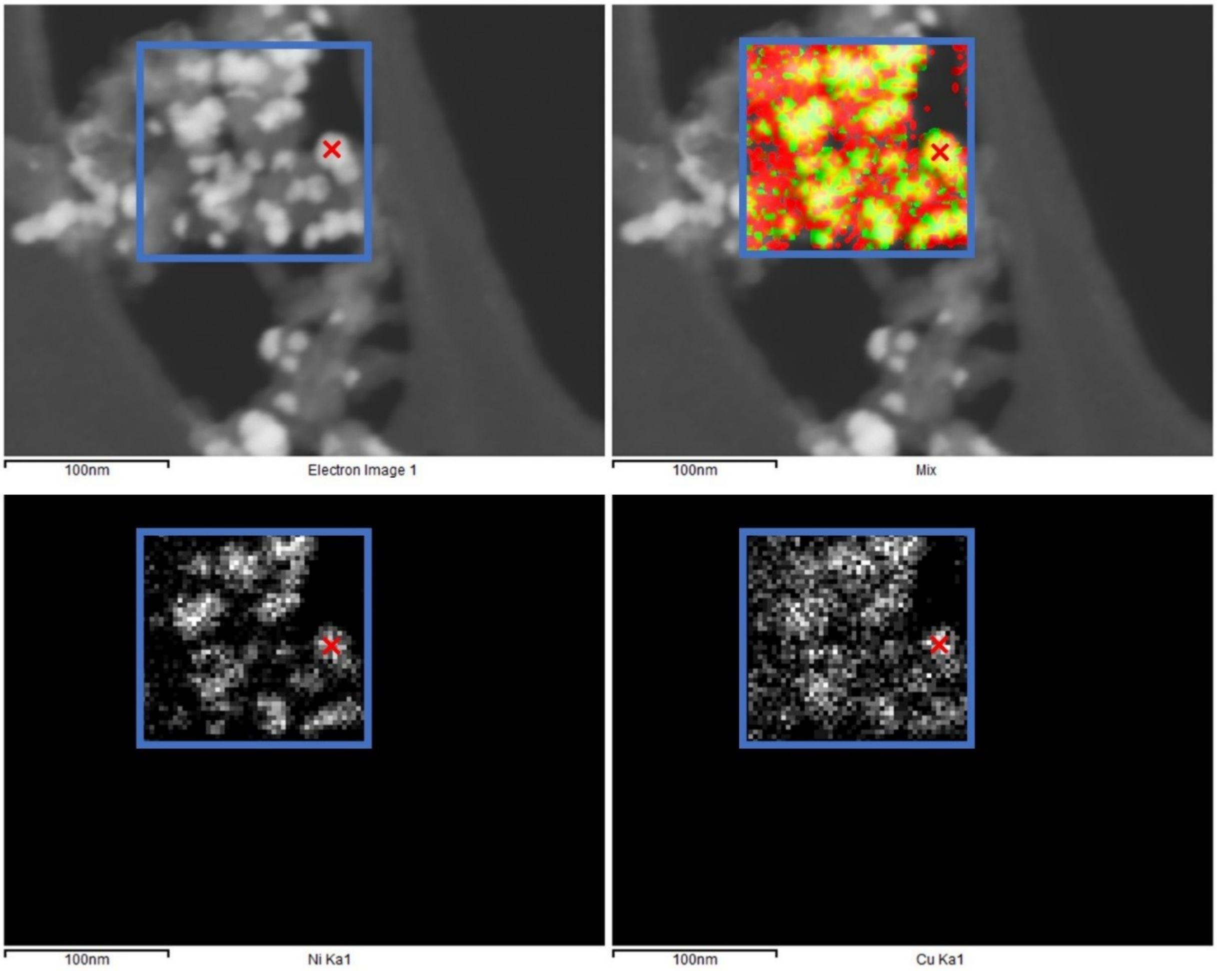

Figure S6: STEM-EDS (Ultra Zeiss). Element mapping for the Ni_3Cu/C-CR sample. Scale bar 100 nm. Identical points are marked in red. Green — Ni, red — Cu, yellow — overlapping Ni and Cu.

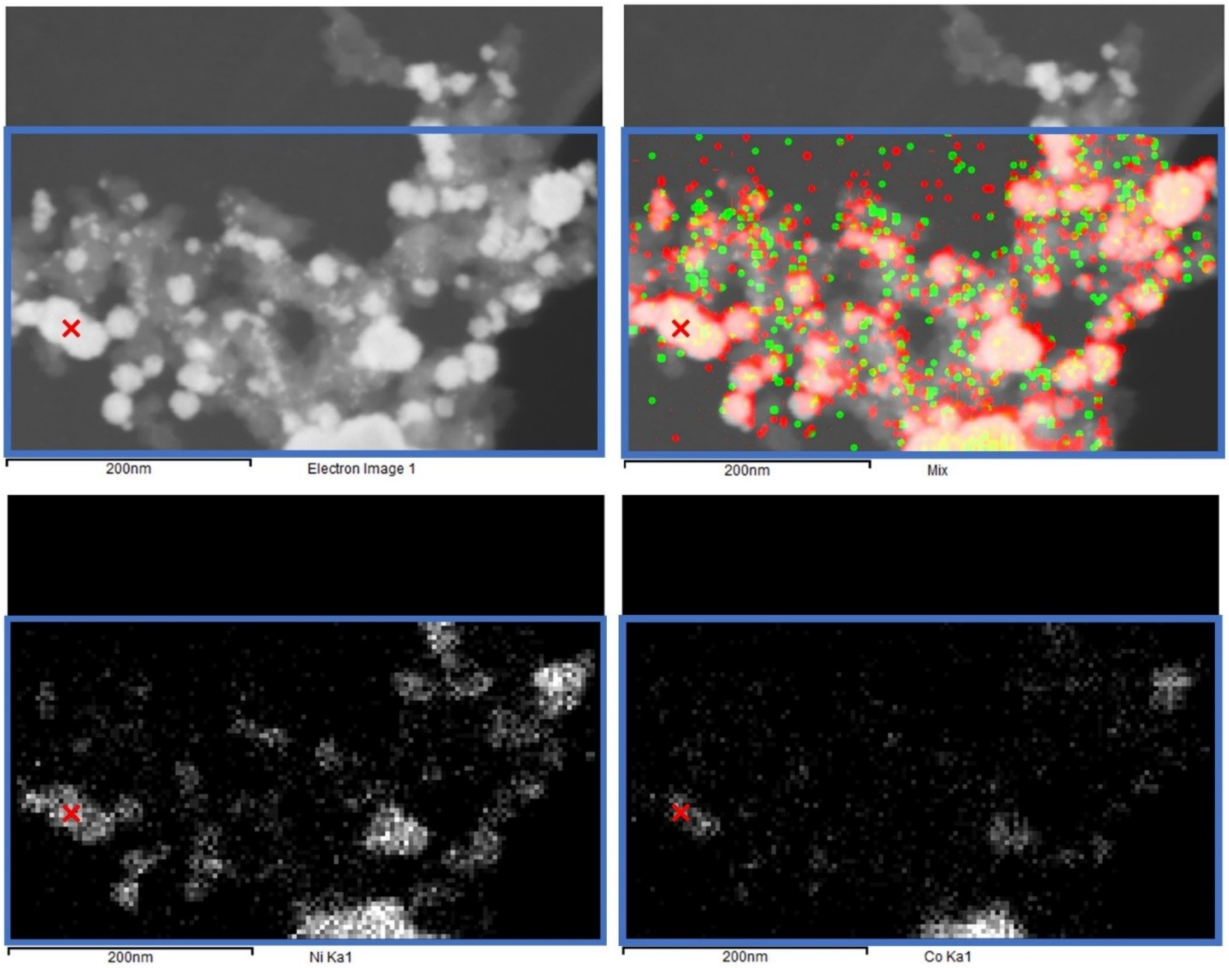

Figure S7: STEM-EDS (Ultra Zeiss). Element mapping for the Ni_3Co/C-CR sample. Scale bar 100 nm. Identical points are marked in red. Green — Ni, red — Fe, yellow — overlapping Ni and Co.

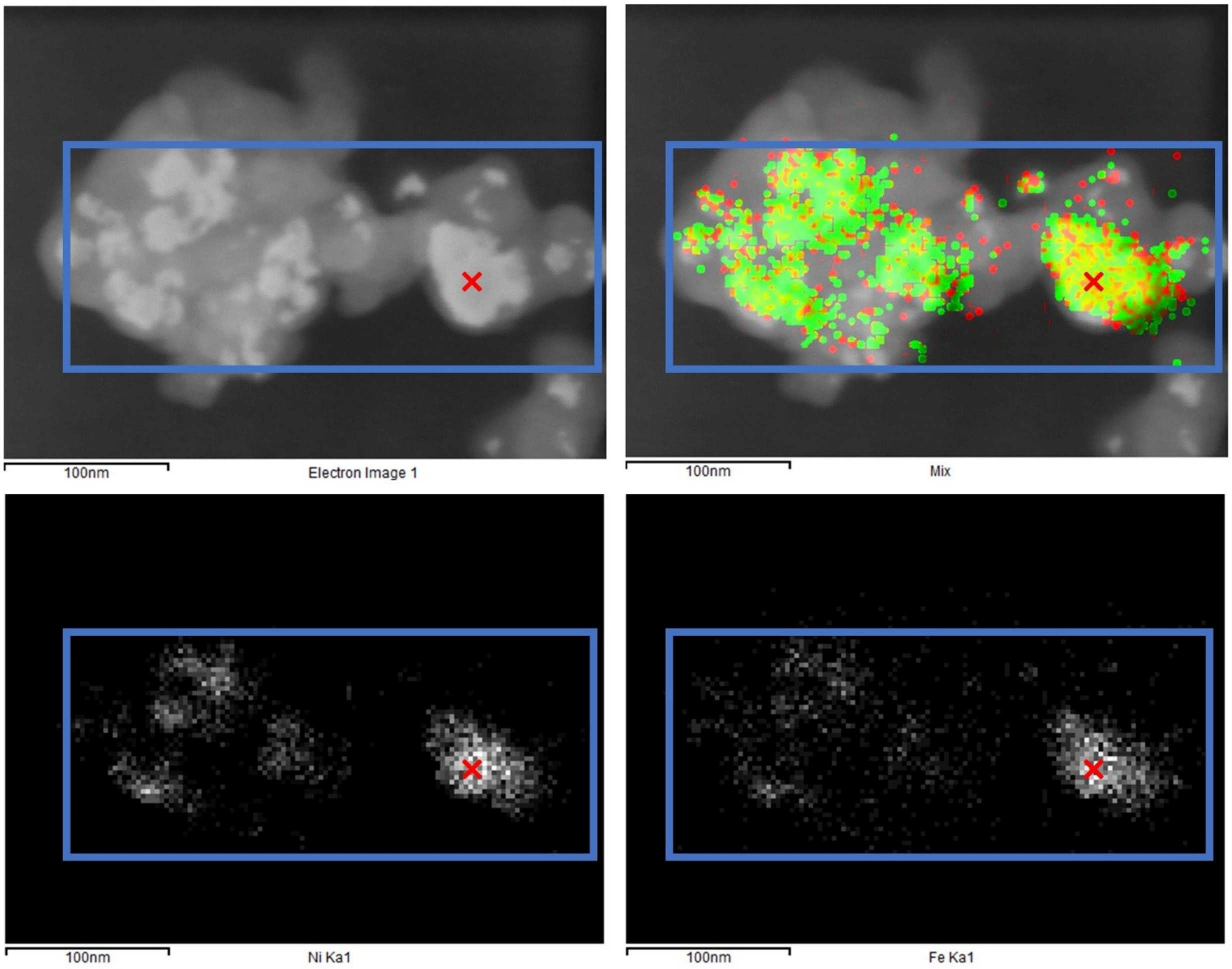

Figure S8: STEM-EDS (Ultra Zeiss). Element mapping for the Ni_3Fe/C-CR sample. Scale bar 100 nm. Identical points are marked in red. Green — Ni, red — Co, yellow — overlapping Ni and Co.

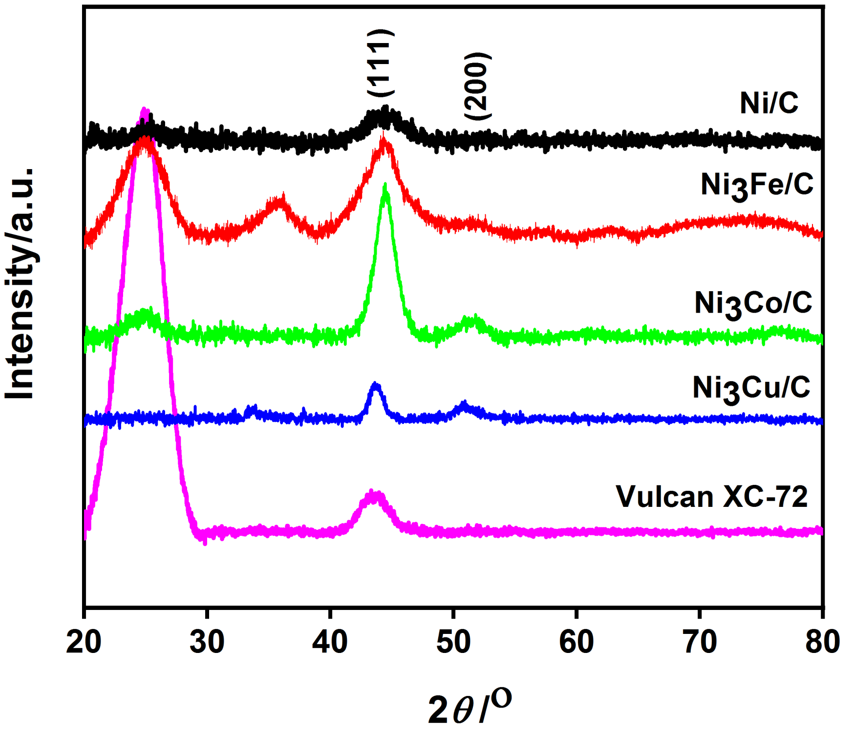

Figure S9: X-ray diffractograms for pristine samples of Ni/C, Ni_3Co/C, Ni_3Cu/C, Ni_3Fe/C synthesised by solvothermal reduction, and carbon (Vulcan XC-72).

Ni, Figure S10 also after annealing.

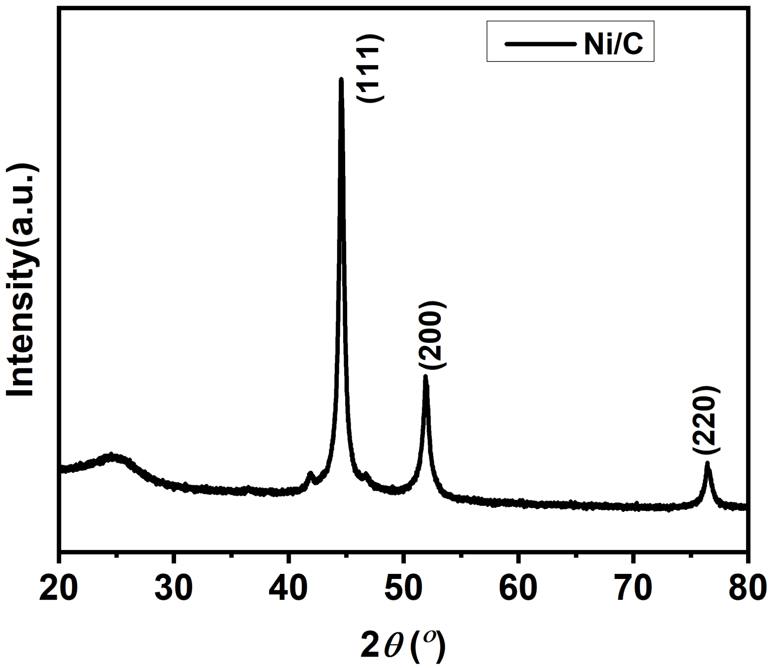

Figure S10: X-ray diffractograms for a sample of Ni/C synthesised by solvothermal reduction with a reduced amount of TOP after annealing at 500 °C. The Ni:TOP ratio was 1:2 for this synthesis.

Figure S11 shows the mass change upon heating the sample in air. A significant reduction in mass is observed in the temperature interval 400 °C through 600 °C. This mass change is the net change corresponding to the oxidation of the carbon support and the uptake of oxygen to form oxides. Figure S12 shows the X-ray diffractograms for samples of Ni/C, Ni_3Co/C, Ni_3Cu/C, and Ni_3Fe/C after having been subjected to TGA. The diffractogram for Ni/C shows a multitude of peaks, some of which may correspond to hydroxide and oxide, but the peaks at $2\theta \approx 42°$ and $2\theta \approx 47°$ present in the sample prior to the TGA (Fig. S10) have now disappeared, and the (111) peak for Ni at 2θ ($2\theta = 44.5°$ has reappeared.

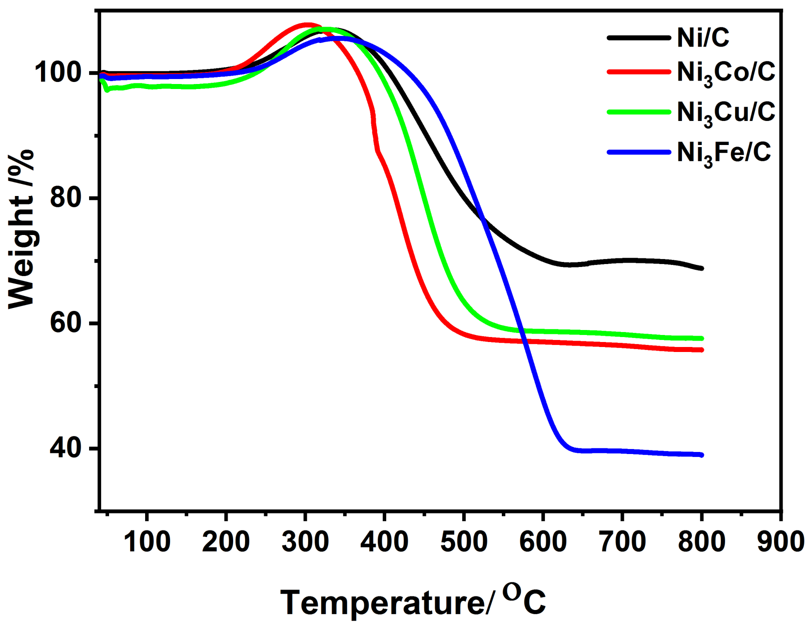

Figure S11: Mass change upon heating the samples of Ni/C, Ni_3Co/C, Ni_3Cu/C, Ni_3Fe/C in air at a rate of 10 °C min^{-1} (TGA). The samples were synthesised by solvothermal reduction.

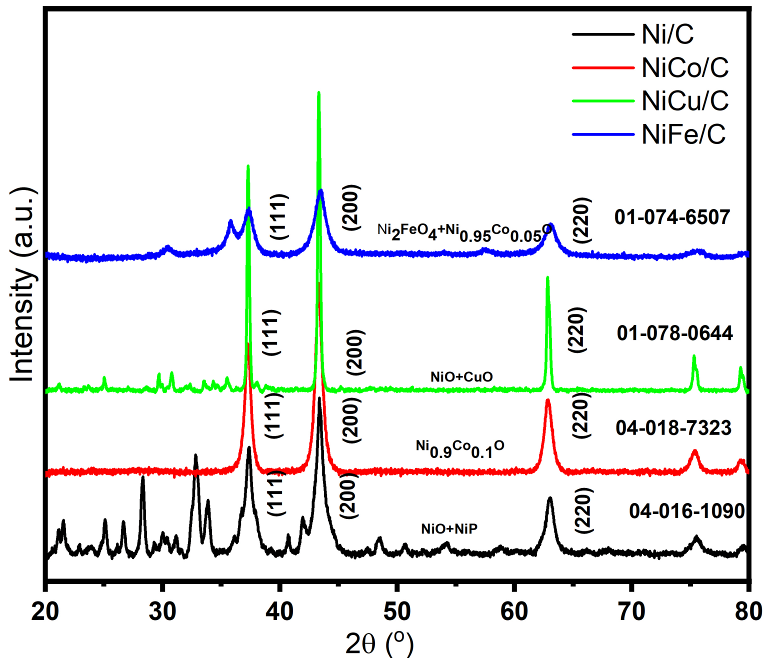

Figure S12: X-ray diffractograms for samples of Ni/C, Ni_3Co/C, Ni_3Cu/C, and Ni_3Fe/C after annealing at 500 °C and removal of the carbon support in TGA. The peak labelled (200) here corresponds to (111) in the previous figure. The samples were synthesised by solvothermal reduction.

Hydrogen temperature-programmed desorption

H_2-TPR spectra are given in Figures S13 and S14 for samples made by chemical reduction and solvothermal reduction, respectively. The results of the deconvolution are given in Tables S1 and S2, respectively.

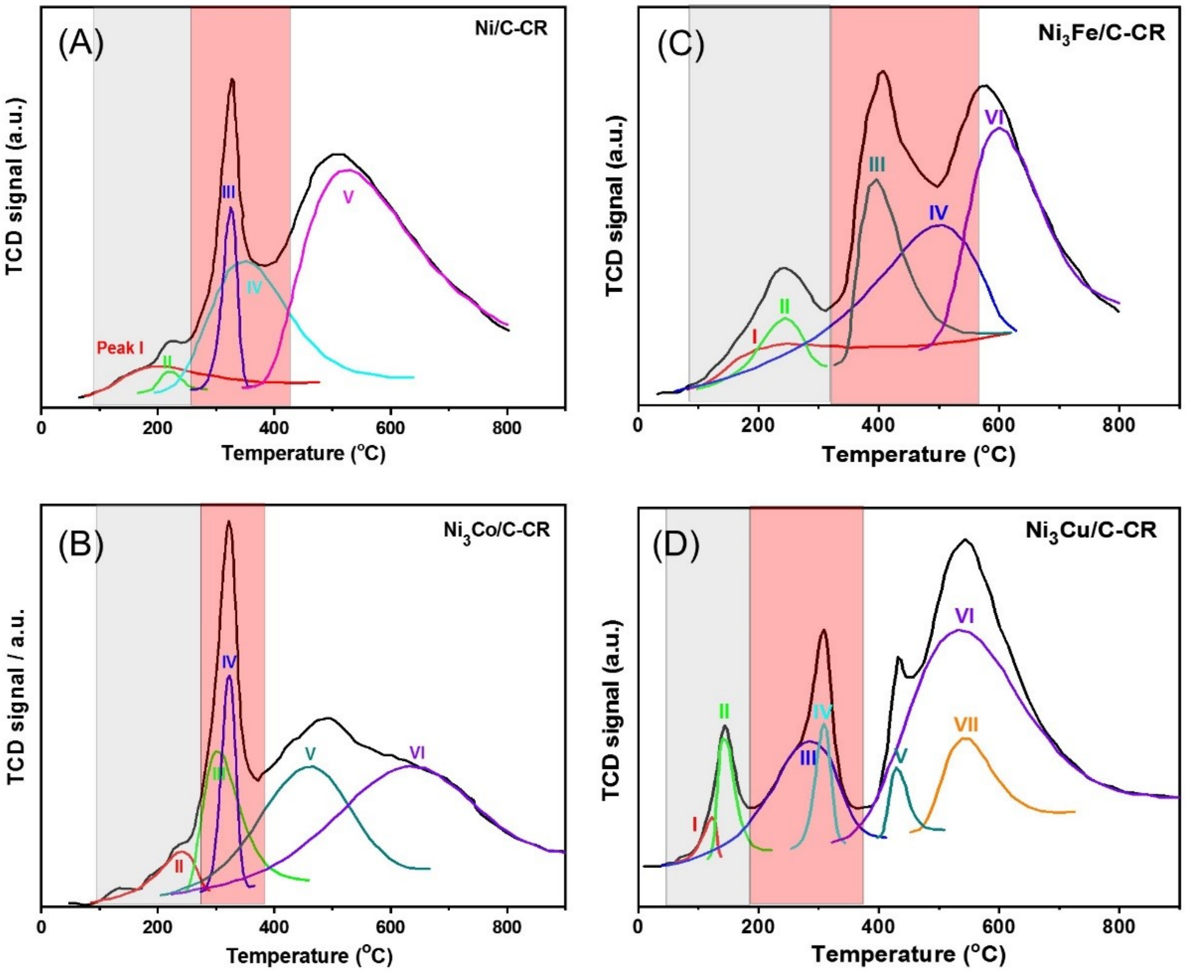

Figure S13: Deconvolution of the H_2-TPR spectra for the catalysts made by chemical reduction.

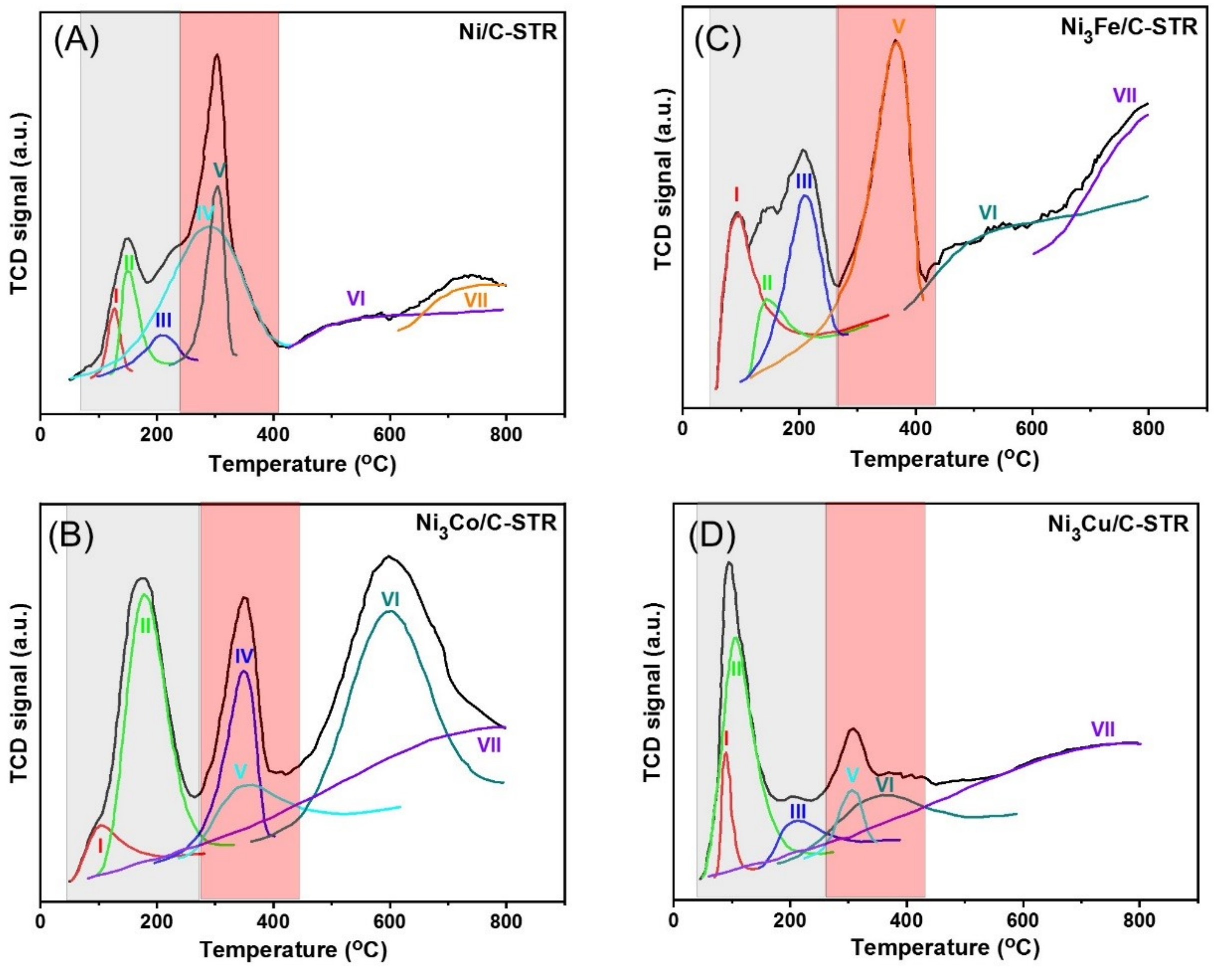

Figure S14: Deconvolution of the H_2-TPR spectra for the catalyst made by solvothermal reduction.

Peak	Ni/C-CR		Ni_3Fe/C-CR		Ni_3Co/C-CR		Ni_3Cu/C-CR	
	T / °C	mmol H_2 per g cat. (ratio)	T / °C	mmol H_2 per g cat. (ratio)	T / °C	mmol H_2 per g cat. (ratio)	T / °C	mmol H_2 per g cat. (ratio)
I	194	0.247 (0.05)	220	0.209 (0.08)	-	-	143	0.145 (0.03)
II	221	0.049 (0.01)	243	0.142 (0.06)	242	0.160 (0.05)	168	0.312 (0.07)
Σ_{I+II}	-	**0.296 (0.06)**						
III	327	0.289 (0.06)	394	0.489 (0.19)	307	0.640 (0.20)	278	0.570 (0.13)
IV	349	1.059 (0.23)	410	0.023 (0.01)	324	0.261 (0.08)	308	0.220 (0.05)
Σ_{III+IV}	-	**1.358 (0.29)**						
V	524	2.953 (0.64)	498	0.802 (0.31)	488	1.734 (0.55)	432	0.068 (0.02)
VI	-	-	599	0.907 (0.35)	690	0.350 (0.11)	494	2.063 (0.48)
VII	-	-	-	-	-	-	551	0.924 (0.21)
Σ_{I-VII}	-	**4.597**	-	**2.572**	-	**3.154**	-	**4.302**

Table S1: H_2 consumption during the H_2-TPR measurements for the catalysts made by chemical reduction.

Peak	Ni/C-CR		Ni_3Fe/C-CR		Ni_3Co/C-CR		Ni_3Cu/C-CR	
	T / °C	mmol H_2 per g cat. (ratio)	T / °C	mmol H_2 per g cat. (ratio)	T / °C	mmol H_2 per g cat. (ratio)	T / °C	mmol H_2 per g cat. (ratio)
I	128	0.127 (0.04)	92	0.225 (0.15)	105	0.184 (0.04)	89	0.108 (0.04)
II	151	0.270 (0.08)	145	0.086 (0.06)	179	1.060 (0.21)	105	0.689 (0.27)
III	210	0.163 (0.05)	211	0.197 (0.13)	-	-	209	0.167 (0.07)
$\Sigma_{I+II+III}$	-	**0.560 (0.17)**						
IV	291	1.472 (0.43)	-	-	349	0.384 (0.08)	-	-
V	305	0.443 (0.13)	368	0.387 (0.26)	350	0.497 (0.10)	306	0.122 (0.05)
Σ_{IV+V}	-	**1.915 (0.56)**						
VI	545	0.374 (0.11)	519	0.195 (0.13)	596	1.507 (0.30)	349	0.358 (0.14)
VII	743	0.608 (0.18)	798	0.406 (0.27)	719	1.352 (0.27)	700	1.117 (0.44)
Σ	-	**3.457**	-	**1.496**	-	**4.984**	-	**2.561**

Table S2: H_2 consumption of the catalyst made by solvothermal reduction during the H_2-TPR measurements.

X-ray photoelectron spectroscopy

Examples of data treatment of X-ray photoelectron spectroscopy (XPS) data are given in Figure S15 for the Fe $2p_{3/2}$ region for a Ni_3Fe/C sample and for the Cu $2p_{3/2}$ for a Ni_3Cu/C sample. Both samples were made by chemical reduction. The deconvolution into peaks corresponding to the various oxidation states of Fe and Cu are shown along with the experimental data. Spectral fitting parameters for Ni_3Cu/C and Ni_3Fe/C are given in Tables S3 and S4. The quantitative contribution from the peaks in Figure S15 are summarised in Table S5 along with those for samples of the other compositions, made by chemical reduction.

Table S3: XPS spectral fitting parameters of the Cu $2p_{3/2}$ line for the Ni_3Cu/C catalysts made by chemical reduction.

BE / eV	FWHM / eV	% of area	Chemical state
932.83	0.75	5.76	Cu(0)
932.08	0.80	3.19	Cu_2O
933.31	1.87	17.53	CuO
934.68	3.07	37.86	CuO
941.46	3.70	26.78	CuO
943.84	1.56	8.89	$Cu(OH)_2$

Table S4: XPS spectral fitting parameters of the Fe $2p_{3/2}$ line for the Ni_3Fe/C catalysts made by chemical reduction.

BE / eV	FWHM / eV	% of area	Chemical state
706.9	1.2	5.49	Fe(0)
713.8	1.2	13.42	Fe_2O_3
712.6	1.6	29.95	Fe_3O_4 (Fe^{3+})
710.0	1.5	10.30	FeOOH
711.0	1.4	22.84	FeOOH
711.9	1.6	17.99	FeOOH

Examples of deconvolution of X-ray photoelectron spectroscopy (XPS) data for the solvothermally synthesised samples are given in Figure S16 for Ni_3Fe/C, Ni_3Co/C and Ni_3Cu/C, which shows the dconvolution of the Fe, Co, and Cu states, respectively. The contribution from all the states included in the deconvolution are given in Table S6.

Survey spectra for catalyst made by solvothermal reduction are given in Figure S17. The

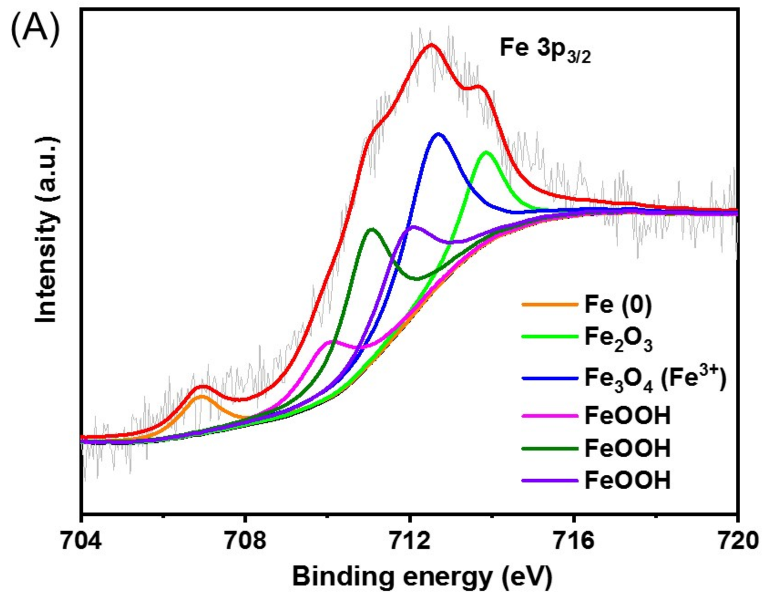

(a)

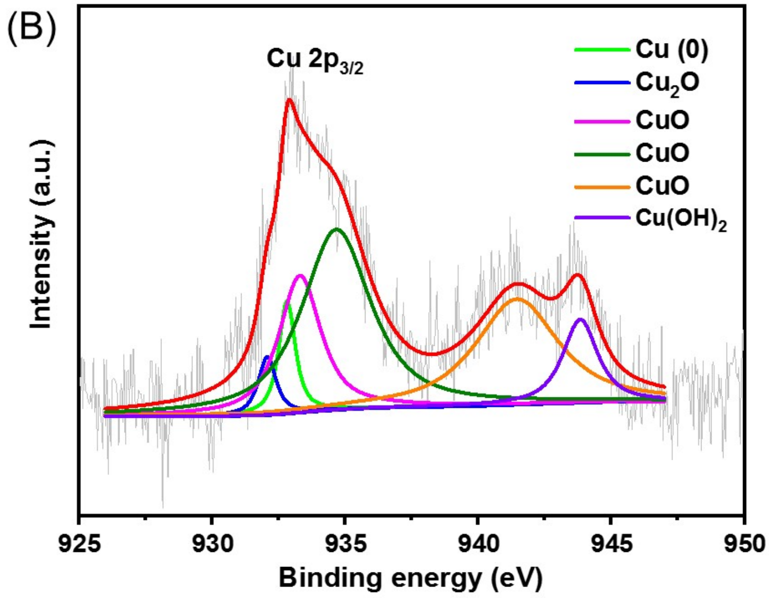

(b)

Figure S15: Deconvolution of HR-XPS spectra for the Fe $2p_{3/2}$ line of Ni_3Fe/C (a) and (b) for the Cu 2p3/2 line of Ni_3Cu/C both made by chemical reduction.

Table S5: XPS spectral fitting parameters of Ni/C catalysts made by chemical reduction, Ni_3Fe/C,Ni_3Co/C and Ni_3Cu/C: binding energy(eV), % of the metallic Ni, atomic ratio and chemical state.

Catalyst	BE / eV	Ni(0)/$\sum$ Ni	Atomic ratio	Chemical state
Ni/C (CR)	852.85	0.13	13.05	Ni
	855.82		36.85	$Ni(OH)_2$
	857.51		12.58	Ni^{3+} (NiOOH)
	861.42		37.51	Satellite
Ni_3Fe/C (CR)	852.75	0.11	11.00	Ni
	854.17		5.81	NiO
	856.00		32.02	$Ni(OH)_2$
	857.45		12.25	Ni^{3+} (NiOOH)
	861.60		36.05	Satellite
Ni_3Co/C (CR)	852.75	0.08	8.15	Ni
	854.17		1.67	NiO
	856.00		35.69	$Ni(OH)_2$
	857.45		13.49	Ni^{3+} (NiOOH)
	861.60		39.69	Satellite
Ni_3Cu/C (CR)	852.75	0.18	7.77	Ni
	854.17		2.73	NiO
	856.00		36.79	$Ni(OH)_2$
	857.45		13.90	Ni^{3+} (NiOOH)
	861.50		38.80	Satellite

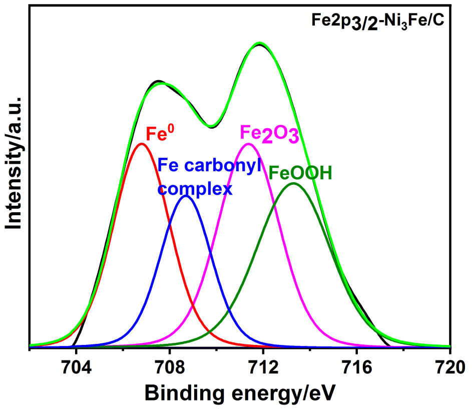

(a)

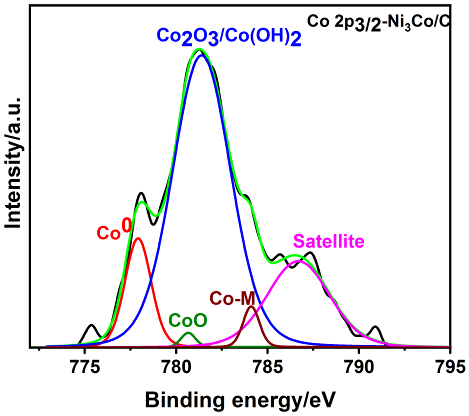

(b)

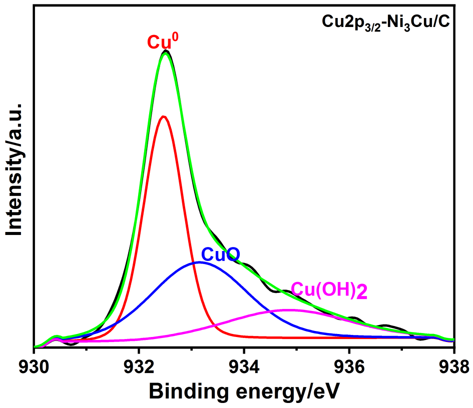

(c)

Figure S16: X-ray photoelectron spectra (XPS) of the solvothermally synthesised samples; 2p region from (a) Ni_3Fe/C, (b) Ni_3Co/C and (c) Ni_3Cu/C.

Catalyst	BE (eV)	atomic ratio %	FWHM (eV)	Chemical State		BE (eV)	% atomic ratio	FWHM (eV)	Chemical State
Ni/C	852.93	19.98	1.22	Ni(0)					
	856.50	54.90	2.62	$Ni_2O_3/Ni(OH)_2$					
	861.67	25.13	3.73	Satellite					
Ni_3Co/C	852.81	19.05	1.70	Ni(0)	Co	781.35	67.36	3.68	$Co_2O_3/$ $Co(OH)_2$
	854.72	4.17	2.02	NiO		786.75	18.95	3.67	Satellite
	856.05	48.09	2.91	$Ni_2O_3/Ni(OH)_2$		777.92	10.03	1.52	Co(0)
	861.37	26.61	4.14	Satellite		784.15	3.67	1.27	Co(0)
	858.66	2.08	2.02	Ni_3Co/ NiCo					
Ni_3Cu/C	852.76	30.42	1.16	Ni(0)	Cu	932.47	45.37	0.90	Cu(0)
	853.66	13.93	2.11	NiO		933.14	37.38	2.15	CuO
	856.05	27.71	2.22	$Ni_2O_3/Ni(OH)_2$		934.83	15.25	2.75	$Cu(OH)_2$
	858.07	6.95	2.11	NiCu					
	861.06	20.99	4.74	Satellite					
Ni_3Fe/C	852.78	38.85	1.91	Ni(0)	Fe	706.90	24.55	2.54	Fe(0)
	856.02	34.00	2.74	$Ni_2O_3/Ni(OH)_2$		708.77	15.89	2.97	FeO
	858.74	6.24	2.25	Ni_3Fe/ NiFe		711.84	38.68	2.97	Fe_2O_3
	854.15	6.78	1.98	NiO		713.89	13.64	2.49	FeOx
	861.42	14.14	3.10	Satellite					

Table S6: XPS spectral fitting parameters of Ni/C catalyst made by solvothermal reduction, Ni_3Co/C, Ni_3Cu/C and Ni_3Fe/C: binding energy (in eV),% atomic ratios, FWHM (in eV) values and chemical states.

composition as evaluated from these spectra are given in Table S6, which also includes data for the samples made by chemical reduction as well as a comparison with EDS results for the same samples.

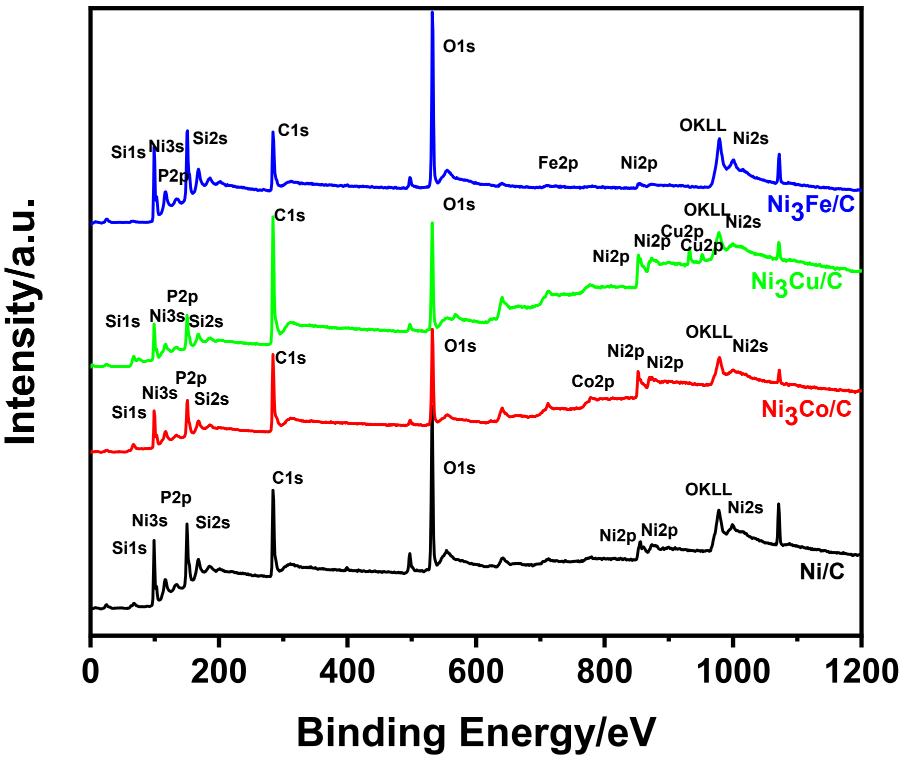

Figure S17: X-ray photoelectron spectra (XPS) for samples of Ni/C, Ni_3Co/C, Ni_3Cu/C, and Ni_3Fe/C synthesised by solvothermal reduction.

Table S7 summarizes the relative element concentrations (in at%) obtained via the XPS (from survey spectra such as those in Figure S17) and EDS methods, comparing the bulk and surface compositions of the catalysts. The surface of the CR catalysts comprises carbon, nickel, secondary transition metal, oxygen and boron. Boron was not detected in the CR samples by EDS (Table S7) due to the low intensity of the B K_α line (185 eV) overlapping with that of carbon (277 eV). The surface Ni/M ratios are significantly higher than the bulk ones, showing the surface segregation of Ni, especially for Ni-Co, which is in a good agreement with the element maps (Figure S7). The surface of the catalysts is more oxidised than the

bulk, which is seen from the comparison of the EDS and XPS data. The high O/(Ni+M) stoichiometry, exceeding 2 for all CR catalysts, shows that the surface of the catalysts suffers from an extensive passivation, clearly seen from Figure 8 in the main article. A fully oxidised is to be expected. However, below we show by electrochemistry that at least a part of the metallic nickel surface stays reduced after synthesis or becomes so during potential cycling. (For the STR samples data from Raman spectroscopy, presented in the main article, indicate that the activation procedure for the STR samples leads to a more or less fully reduced surface for Ni/C and a little less so for the other compositions.) The STR samples show very high tendency of Ni atoms to segregate of the surface (Ni/M ratio in Table S7), probably due to the diffusion of the atoms during the thermal annealing. At the same time, the surface of the STR samples is much less oxidised compared to the CR samples (O/(Ni+M) ratio, Table S7). This observation might be related to the higher P content on the surface, which prevents Ni from oxidation. The degree of surface oxidation for Ni/C-STR is close to that of the bulk, whereas the degree of the surface oxidation is slightly higher for the bimetallic STR catalysts in comparison to the bulk oxygen content. This additionally indicates that Ni doping by Co, Fe and Cu increases the affinity of the catalysts to oxygen.

Activation of electrodes

Figures S18 and S19 show cyclic voltammograms recorded during activation for Ni_3Fe/C synthesised by the chemical reduction method and the solvothermal synthesis, respectively.

In studies of the hydrogen evolution reaction in acid solution, any prolonged cathodic polarisation of the working electrode the associated anodic polarisation of a Pt counter electrode may result in some Pt dissolution. This dissolved Pt may deposit at the working electrode[1–3] and lead to an apparent activation of non-precious catalysts there. We do not expect any significant Pt dissolution in our case since the Pt counter electrode was polarised primarily to negative potentials, since the measurements were performed in alkaline solutions,

Catalyst	Method	Ni/M at. ratio	O/(Ni+M) at. ratio	Element concentration / at. %				
				C	O	Ni	M	B or P
Ni/C-CR	XPS	-	3.36	85.40	10.45	3.10	-	1.04
	EDS	-	0.30	80.26	3.09	10.23	-	N/A*
Ni_3Fe/C-CR	XPS	6.00	7.33	82.33	12.85	1.50	0.25	3.07
	EDS	3.27	0.91	74.78	11.88	10.03	3.07	N/A
Ni_3Co/C-CR	XPS	66.43	2.33	83.14	11.00	4.65	0.07	1.13
	EDS	2.46	0.34	69.85	5.02	10.58	4.30	N/A
Ni_3Cu/C-CR	XPS	17.87	2.42	80.65	13.7	5.36	0.30	1.32
	EDS	4.08	1.32	70.33	11.25	6.89	1.66	N/A
Ni/C-STR	XPS	-	0.53	35.96	20.14	37.95	-	5.95
	EDS	-	0.59	76.41	6.96	11.85	-	4.79
Ni_3Fe/C-STR	XPS	334.8	0.79	34.2	26.62	33.66	0.10	5.41
	EDS	4.12	0.36	87.88	2.67	6.45	1.57	1.28
Ni_3Co/C-STR	XPS	42.9	0.81	51.48	18.62	22.55	0.53	6.82
	EDS	3.96	0.41	77.27	6.10	11.75	2.97	1.91
Ni_3Cu/C-STR	XPS	40.6	1.15	62.02	16.44	14.00	0.34	7.19
	EDS	2.86	0.10	81.96	1.40	10.55	3.69	2.39

Table S7: Atomic composition of the catalysts characterised by the XPS and EDS methods. The last rightmost column gives the boron content for the CR samples and phosphorous for the STR samples.

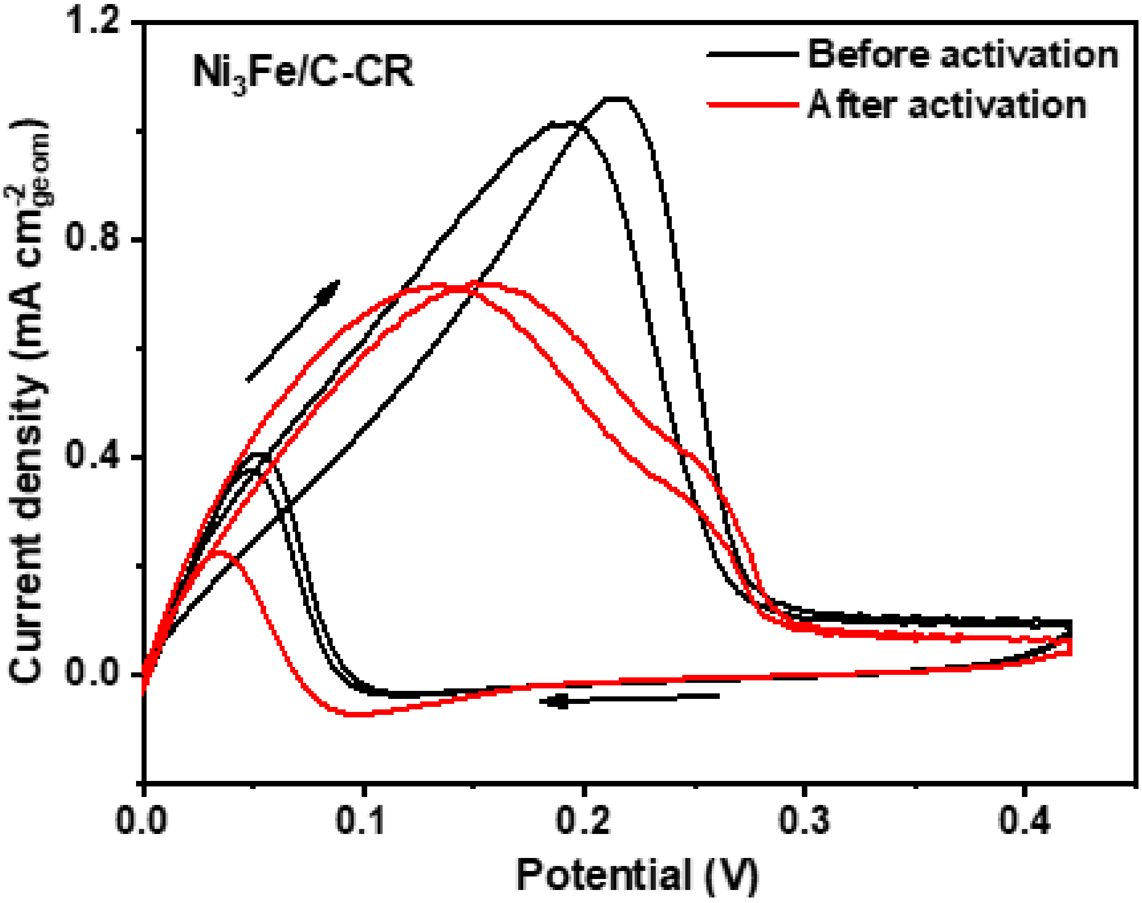

Figure S18: HOR polarisation curves on the as-prepared Ni_3Fe/C-CR catalyst (black) and on the same electrode after the electrochemical pre-activation procedure. H_2-purged 0.1 mol dm^{-3} KOH, 25 °C, sweep rate 1 mV s^{-1}, rotation rate 1600 rpm, catalyst loading 400 μg $cm^{-2}{}_{geom}$. The arrows show the direction of the potential sweeps.

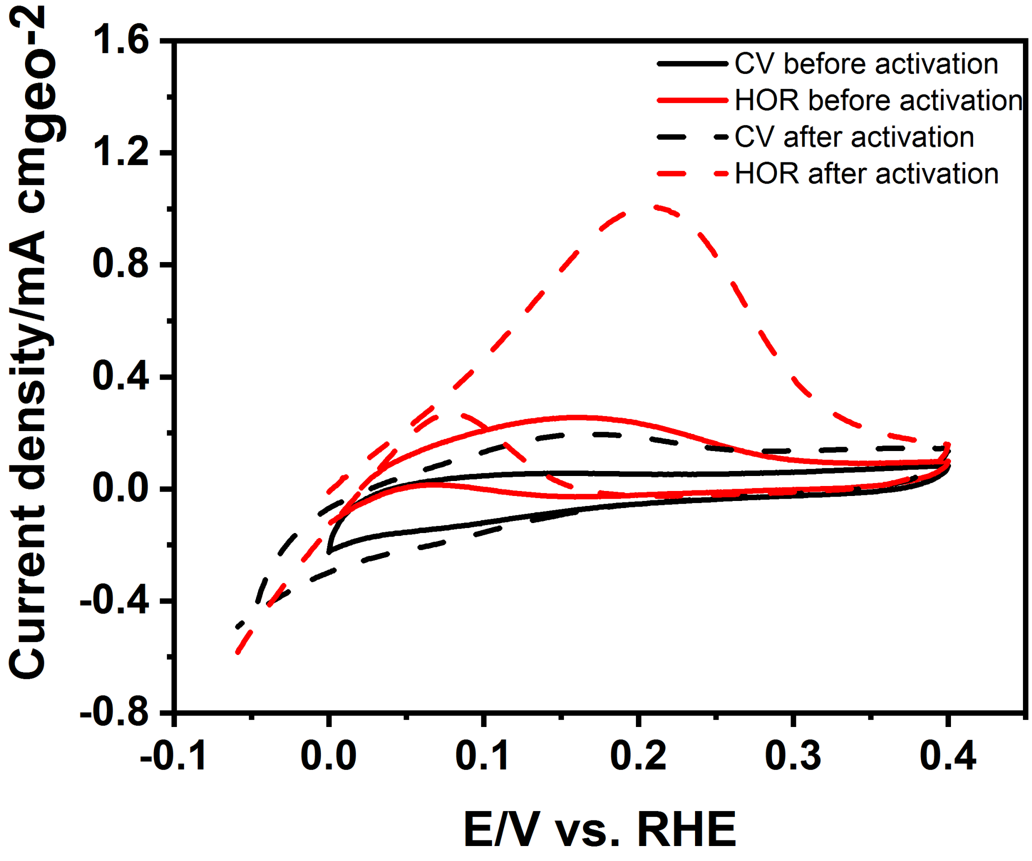

Figure S19: HOR polarisation curves on the as-prepared Ni_3Fe/C-STR catalyst (black) and on the same electrode after the electrochemical pre-activation procedure. H_2-purged 0.1 mol dm^{-3} KOH, 25 °C, sweep rate 1 mV s^{-1}, rotation rate 1600 rpm, catalyst loading 505 μg $cm^{-2}{}_{geom}$.

since the counter electrode was physically separated from the working electrode compartment for the samples synthesised by chemical reduction, and since the counter-electrode area was approximately ten times larger than the working electrode. These are all factors that would minimise Pt dissolution. It is nevertheless desirable to verify the absence of any effects due to Pt dissolution explicitly. We do this by comparing the voltammograms for our Ni-based catalyst to the currents at a polycrystalline Pt rotating disc electrode under the same conditions.

Figure 20(a) shows current vs. potential for a polycrystalline Pt rotating disc electrode in the potential range from 0 V through 1 V in a hydrogen-purged solution of KOH. The current reaches a plateau at 0.2 V, at which it becomes totally mass-transfer controlled as demonstrated in the plot of the current at 0.385 V vs. the square root of the angular velocity in Figure 20(b). At 0.8 V the current decreases sharply due to the onset of Pt oxide formation. The Pt and Ni_3Fe/C results are plotted jointly in Figure S21. The activated Ni_3Fe/C electrode shows no signs of any mass-transfer limited current as the Pt electrode does, and instead of decaying at potentials associated with Pt oxide formation (0.8 V) decays after reaching a potential associated with the formation of $Ni(OH)_2$ (0.2 V, see discussion below). This shows that the Ni-based electrodes are unaffected by the Pt counter electrode and that the activation procedure changes the catalysts themselves rather than doping them with Pt. In fact, the current on the return sweep can be taken as the current corresponding to any catalytically active impurity at the surface. Since this current is zero, so is the influence of impurities (including Pt).

Deconvolution of voltammograms

The deconvolution of sample voltammograms in argon are given in Figure S22 for all compositions and both synthesis series. The deconvolution was made as explained in the main article. The derivative of the current with respect to electrode potential, computed from

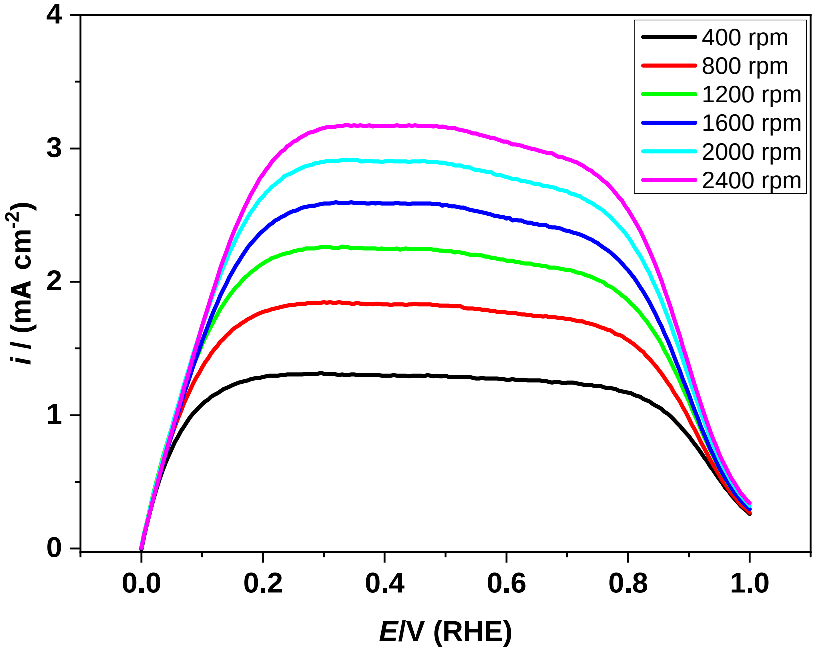

(a) Current vs. potential

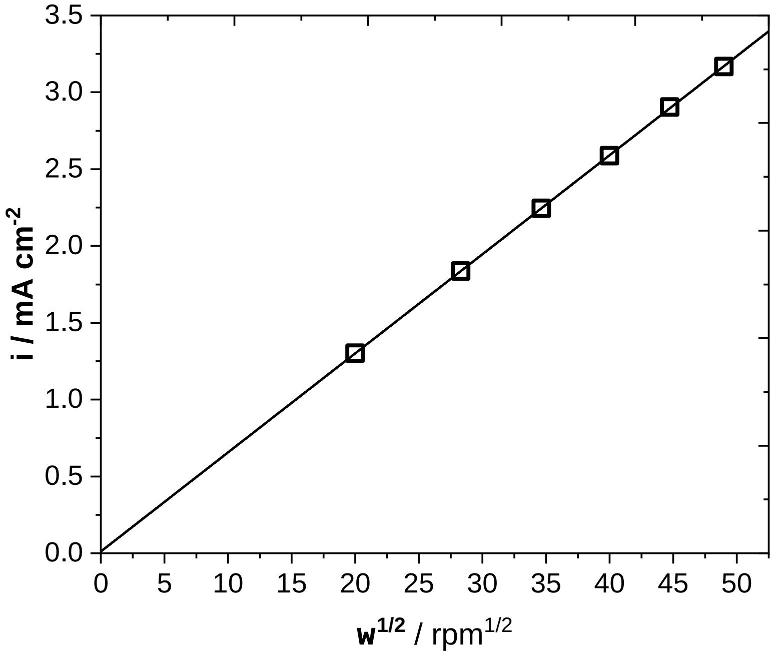

(b) Current vs square root of angular velocity

Figure S20: (a) Current vs. potential for a polycrystalline Pt electrode in a hydrogen-saturated solution of 01. mol dm^{-3} at the angular velocities ω given in the legend. The sweep rate was 5 mV s^{-1}. (b) Plot of currents in (a) at 0.385 V vs. $\omega^{1/2}$.

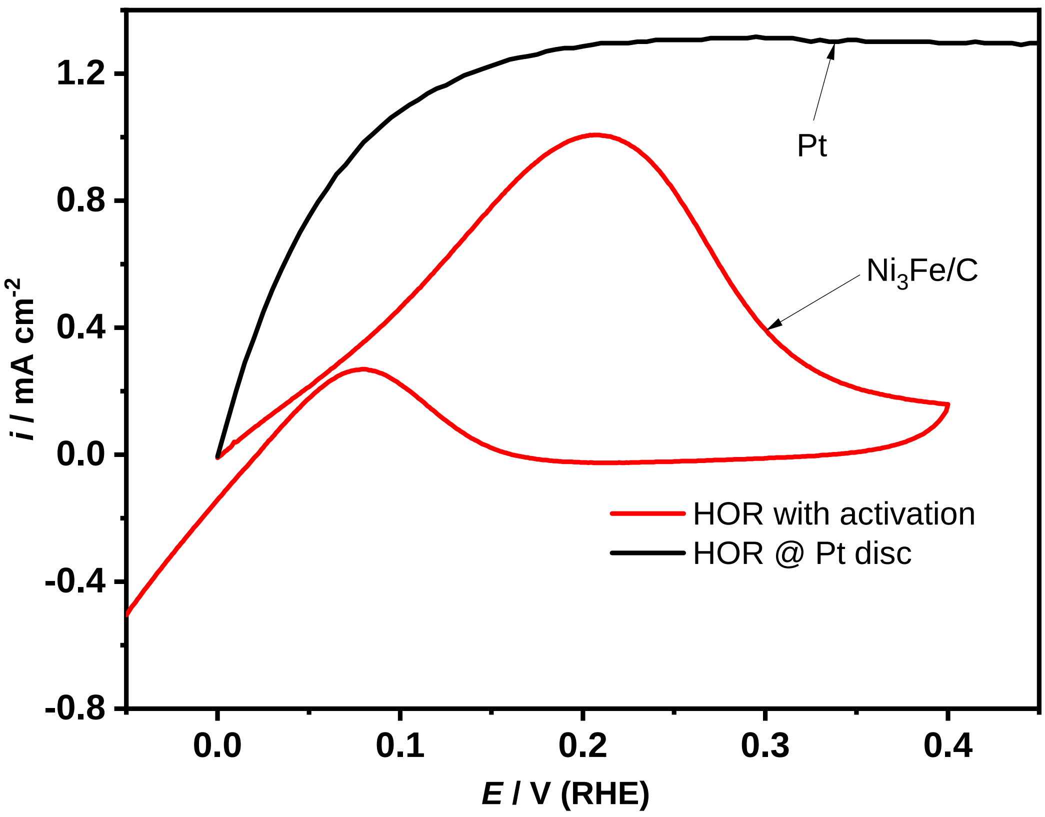

Figure S21: Cyclic voltammogram for Ni_3Fe/C (activated electrode, catalyst prepared by solvothermal reduction, data set from Figure S19) with current vs. potential for a Pt electrode included (data set for 400 rpm from Figure S20).

voltammograms recorded in hydrogen-purged solutions, is included in the figure.

Evaluation of electrochemical surface area and exchange current density

For the samples manufactured by chemical reduction, we used charge of peaks I and II in Figure 12 in the main article to calculate ECSA values, assuming a charge density of 514 $\mu C\ cm^{-2}$.

To ensure a proper reduction of the sample surface at the start of the experiments for the solvothermally synthesised samples, we used -0.06 V for the lower vertex potential in cyclic voltammetry. (Prior to this, the samples were preconditioned through sweeps from -0.2 V through 0.4 V in an Ar-purged electrolyte as described in the main article.) Whereas Oshchepkov *et al.*[4] found no significant influence on rotation for Ni rods and electrodeposited Ni on glassy carbon electrodes, in our case the currents in the cyclic voltammograms were lower with rotation than without. This is illustrated in Figure S23, comparing CVs with and without rotation for a Ni catalyst prepared by solvothermal reduction.

We assign the differences between the voltammograms in Figure S23 recorded with and those recorded without to hydrogen in the solution, generated as the potential is scanned from -0.06 V to 0 V. If the electrode is rotated this generated hydrogen is expected to be removed from the vicinity of the electrode surface by convection, whereas if the electrode is not rotated the hydrogen will only disappear from the electrode surface by the slower process of diffusion. As the potential passes 0 V any hydrogen remaining at the electrode-electrolyte interface will be oxidised, and since the amount of hydrogen is expected to be lower with rotation the current above 0 V is consequently smaller with rotation than without. Similar experiments at Pt appears to corroborate this interpretation, as they showed a significant influence of rotation even with lower vertex potentials equal to 0 V, as illustrated in Figure 24(a). In this case there is a large overshoot in the current without rotation both when the lower

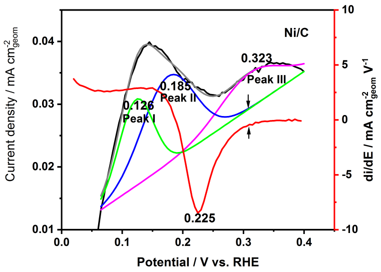

(a) Ni by chemical reduction

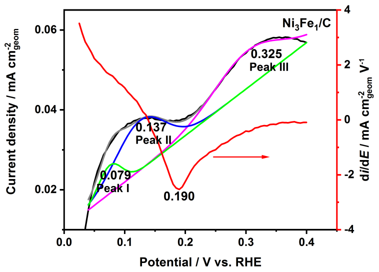

(b) Ni_3Fe by chemical reduction

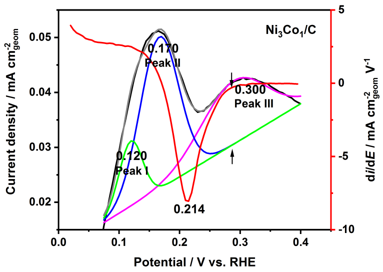

(c) Ni_3Co by chemical reduction

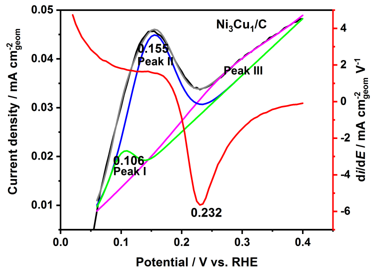

(d) Ni_3Cu by chemical reduction

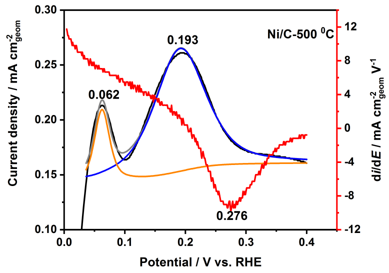

(e) Ni by solvothermal reduction

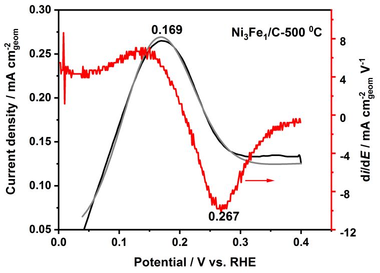

(f) Ni_3Fe by solvothermal reduction

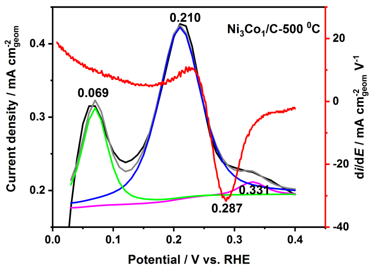

(g) Ni_3Co by solvothermal reduction

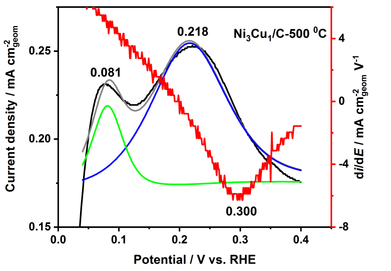

(h) Ni_3Cu by solvothermal reduction

Figure S22: Deconvolution of the forward (anodic) sweep of the CVs (black curves) for (a) Ni/C, (b) Ni_3Fe/C, (c) Ni_3Co/C, and (d) Ni_3Cu/C synthesised by chemical reduction (left axis). In the deconvolution presented the CV has been attributed to a contribution from a low-potential process (I, green curve), an intermediate-potential process (II, blue curve), and a a high-potential process (III, purple curve). The derivative di/dE (red curve, right axis) of the HOR polarisation curve obtained from the forward sweeps of the stable-response HOR polarisation. A similar set of deconvolution of the forward (anodic) sweep of the CVs in argon and the derivative di/dE in hydrogen is shown in (e) for Ni/C, (f) for Ni_3Fe/C, (g) for Ni_3Co/C, and (h) for Ni_3Cu/C synthesised by solvothermal reduction.

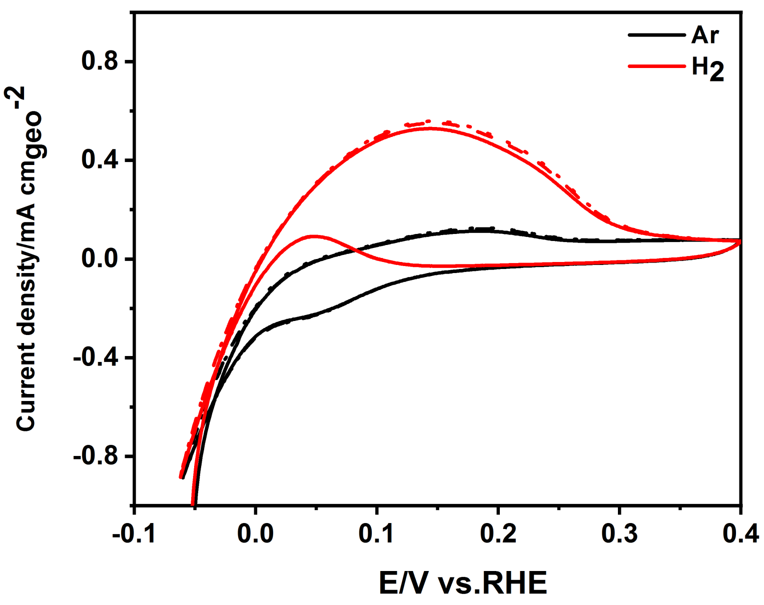

(a)

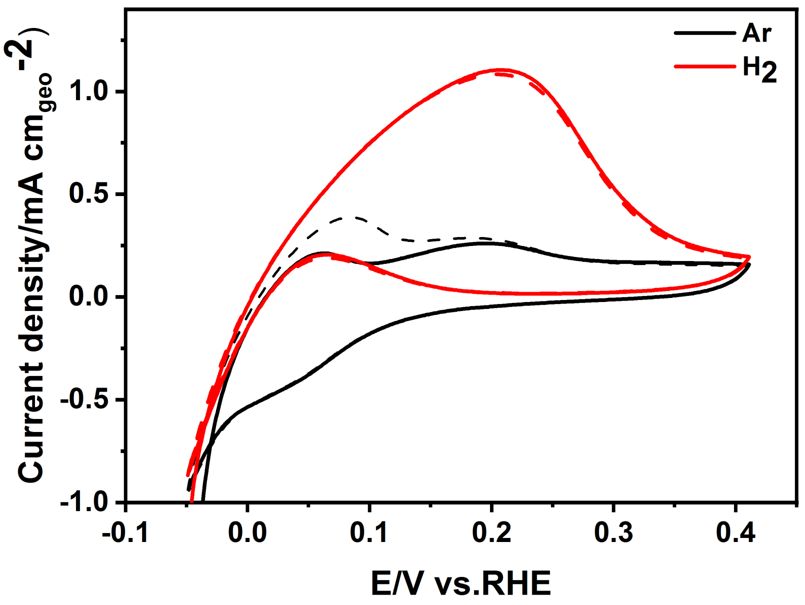

(b)

Figure S23: Comparison of cyclic voltammograms with rotation (1600 rpm) (a) and without rotation (b) in argon- and hydrogen-purged solutions as indicated. The sweep rate was 1 mV s^{-1}, and the sample was a Ni catalyst prepared by solvothermal reduction and supported on carbon. (First (solid lines) and second cycles (dashed lines) are shown.)

vertex potential is 0 V and when the lower vertex potential is -0.06 V. These results may indicate that a lower vertex potential of 0 V may be more appropriate. However, hydrogen still appears to influence the voltammograms for Pt even with a lower vertex potential of 0 V, c.f. Figure 24(b), possibly owing to adsorbed hydrogen at the electrode surface.

From Figure S24 it is clear that the hydrogen underpotential deposition (H-UPD) peaks at Pt are intact and superposed on a sloped baseline, and a similar feature is apparent in the Ni results, perhaps a little less pronounced though. We therefore evaluated the catalyst surface area from the charge by integration of the peak remaining after subtracting a sloped baseline from the inflection point prior to the peak to the inflection point above the peak. It was difficult to establish an automated routine for establishing the inflection points, and these were therefore chosen manually for each case. For the solvothermally synthesised samples we evaluated the surface charge in this manner both for samples with rotation and without, and results were reasonably consistent.

The procedure is illustrated in Figure S25 for five different samples in Ar, three without rotation and two with. The figure shows the positive part of the positive-going voltammogram, the peak after subtraction of a straight baseline, and the corresponding micropolarisation curves and full voltammograms in hydrogen-purged solutions.

The charges are similar for two of the three samples without rotation and the samples being rotated, whereas the charge for one of the samples without rotation the charge is apparently larger. However, this is to quite an extent reflected in a higher slope in the micropolarisation curve for the latter, Figure S24 (c). We therefore ascribe these differences in part to a slightly larger loading at the sample 2 electrode, and the normalised charge transfer conductance.

The electrochemical surface area was analysed in a similar fashion for the Ni_3Co/C, Ni_3Cu/C, and Ni_3Fe/C samples.

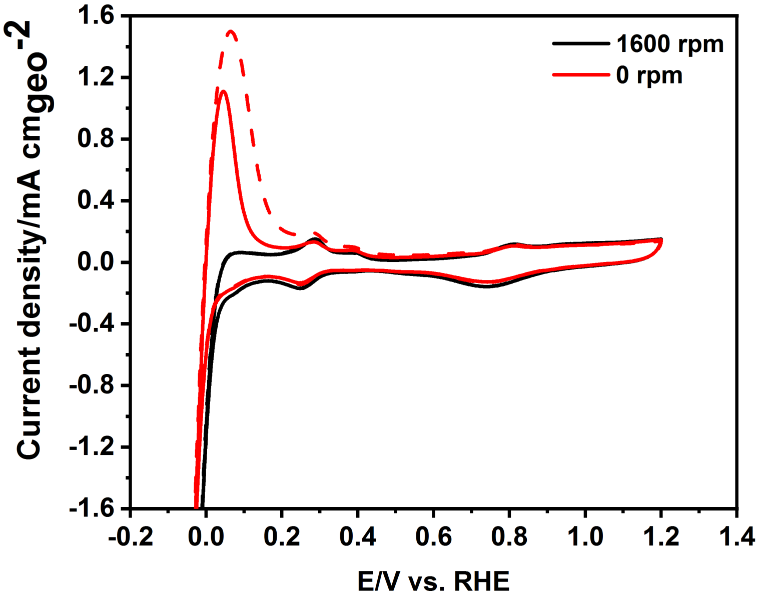

(a)

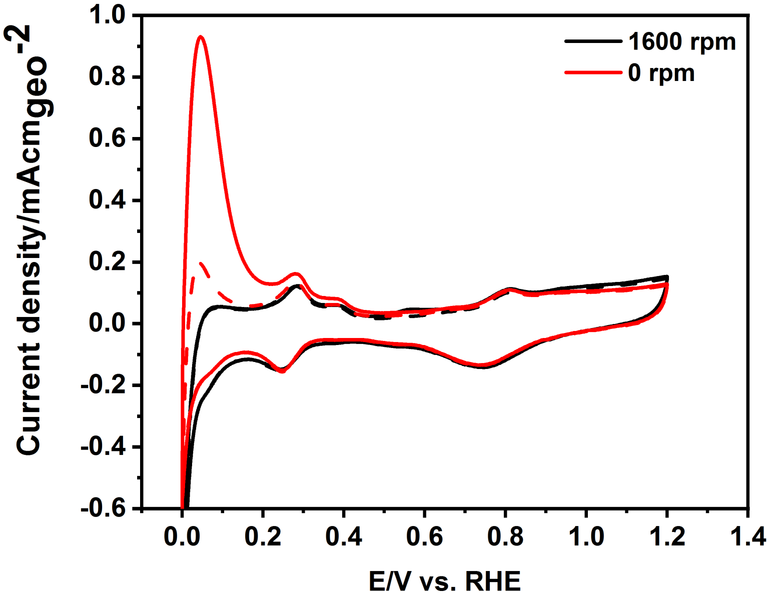

(b)

Figure S24: Comparison of cyclic voltammograms for Pt on carbon supported in argon-purged solutions with rotation (1600 rpm) and without with a lower vertex potential of -0.06 V (a) and with a lower vertex potential equal to 0 V (b). The sweep rate was 1 mV s^{-1}. The solid lines represent the first cycle and the dashed the second. (For the argon-purged solution the first and second cycles more or less overlap.)

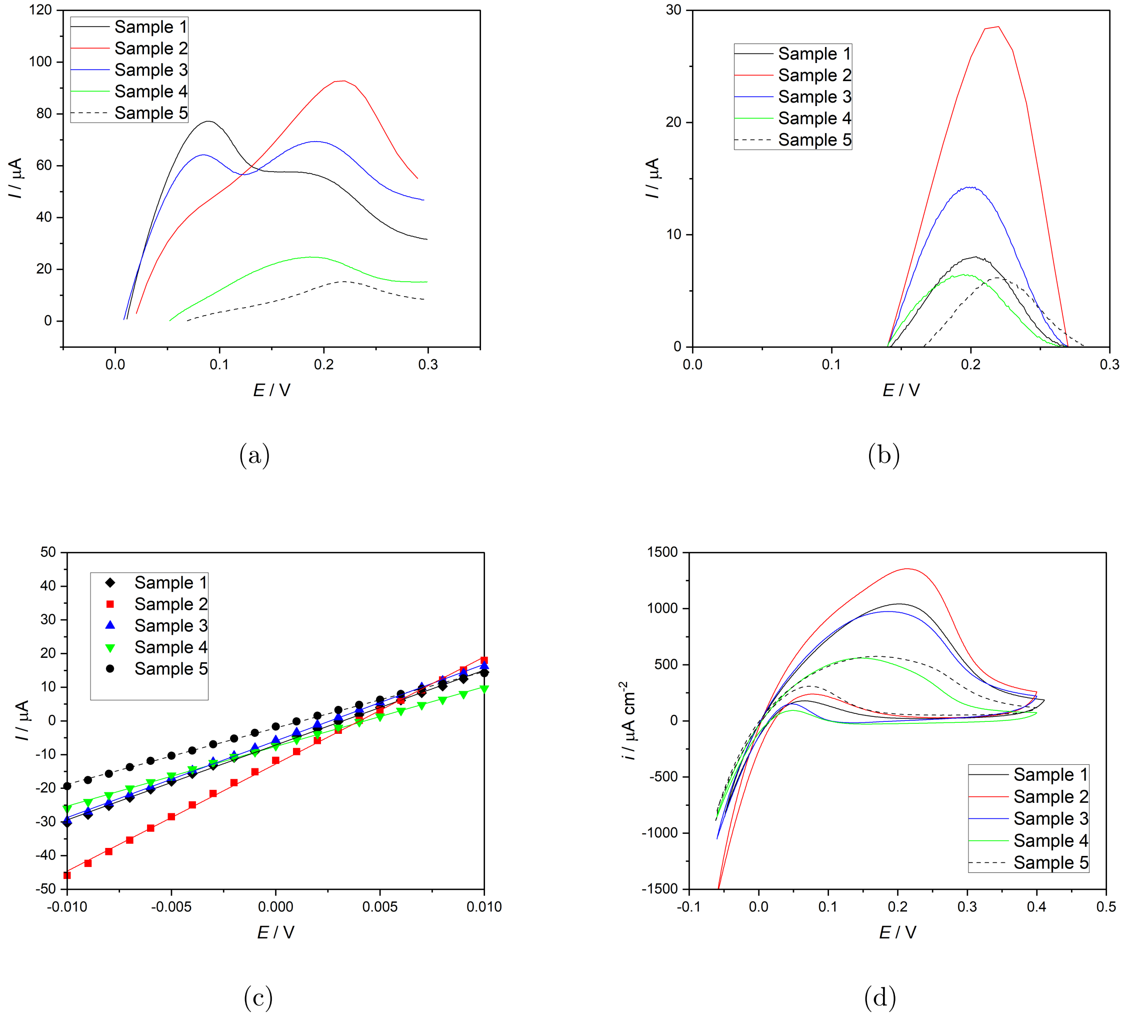

Figure S25: (a) The positive part of cyclic voltammograms (in A) for five different samples of Ni on carbon (Ni/C) in argon. Samples 1 (black curve), 2 (red curve), and 3 (blue curve) were recorded without rotation. Samples 4 (green curve) and 5 (black dashed curve) were recorded with rotation. The sweep rate was 1 mV s^{-1}. For samples 1 through 4 the lower and upper inflection points were selected as 0.14 V and 0.27 V, respectively. For sample 5 they were 0.17 V and 0.28 V. (b) The voltammetic peak after subtraction of a straight baseline for the same samples. (c) The corresponding micropolarisation curves for the same samples. (d) The full cyclic voltammograms (current density) for the same samples in A cm^{-2} based on the geometric electrode area.

Microkinetic modelling

Reaction mechanism

The limiting currents for the hydrogen oxidation in the same cell as that used for the experiments with the Ni-based catalysts were much larger than the peak currents in the voltammograms for the Ni-based catalysts. We therefore assume that the transport of hydrogen to the surface,

$$\mathrm{H_2(aq, y = \infty)} \overset{D_{\mathrm{H_2}}, c_{\mathrm{H_2}}(aq, y=\infty), \Omega}{\rightarrow} \mathrm{H_2(ad)} \tag{S1}$$

where $c_{\mathrm{H_2}}(aq, y = \infty)$ is the bulk concentration of hydrogen, is not affecting the reaction rates. We assume the following sequence of steps,[4–6]

$$\mathrm{H_2(ad)} \underset{k^0_{-1}}{\overset{k^0_1}{\rightleftharpoons}} \mathrm{2H(ad)} \tag{S2}$$

$$\mathrm{H_2(aq) + OH^-(aq)} \underset{k_{-2}}{\overset{k_2}{\rightleftharpoons}} \mathrm{H(ad) + H_2O}(\ell) + \mathrm{e^-} \tag{S3}$$

$$\mathrm{H(ad) + OH^-(aq)} \underset{k_{-3}}{\overset{k_3}{\rightleftharpoons}} \mathrm{H_2O}(\ell) + \mathrm{e^-} \tag{S4}$$

for the HOR and also that the reactions

$$\mathrm{OH^-(aq)} \underset{k_{-4}}{\overset{k_4}{\rightleftharpoons}} \mathrm{OH(ad) + e^-} \tag{S5}$$

$$\mathrm{OH(ad) + OH^-(aq)} \underset{k_{-5}}{\overset{k_5}{\rightleftharpoons}} \mathrm{(OH)_2\,(ad) + e^-} \tag{S6}$$

take place at the Ni surface.

In view of recent discussions on the possible participation of adsorbed hydroxyl ions in the hydrogen oxidation[7–10] reaction one might also consider the participation of this species in the reaction mechanism as in[11]

$$\mathrm{H(ad) + OH(ad)} \underset{k_{-3}}{\overset{k_3}{\rightleftharpoons}} \mathrm{H_2O}(\ell) \tag{S7}$$

Governing equations

We label the coverages, i.e. the number of sites occupied (surface excess) by an adsorbate divided by the total number of available adsorption sites, of H, OH^-, and $(OH)_2$ as θ_1, θ_2, and θ_3, respectively. Thus

$$v_1 = \Gamma^2 k_1^0 c_{H_2} (1 - \theta_1 - \theta_2 - \theta_3)^2 - \Gamma^2 k_{-1}^0 \theta_1^2 \quad (S8)$$

$$v_2 = \Gamma k_2 c_{H_2} c_{OH^-} (1 - \theta_1 - \theta_2 - \theta_3) - \Gamma k_{-2} \theta_1 \quad (S9)$$

$$v_3 = \Gamma k_3 c_{OH^-} \theta_1 - \Gamma k_{-3} (1 - \theta_1 - \theta_2 - \theta_3) \quad (S10)$$

$$v_4 = \Gamma k_4 c_{OH^-} (1 - \theta_1 - \theta_2 - \theta_3) - \Gamma k_{-4} \theta_2 \quad (S11)$$

$$v_5 = \Gamma k_5 c_{OH^-} \theta_2 - \Gamma k_{-5} \theta_3 \quad (S12)$$

where Γ is then the surface excess corresponding to $\theta_\mathrm{i} = 1$. The rate constants are

$$k_\mathrm{i} = k_\mathrm{i}^0 \exp\left[(1 - \alpha_\mathrm{i}) FE/RT\right] \; i = 2 \ldots 3, 5 \quad (S13)$$

$$k_{-\mathrm{i}} = k_{-\mathrm{i}}^0 \exp\left(-\alpha_\mathrm{i} FE/RT\right) \; i = 2 \ldots 3, 5 \quad (S14)$$

and

$$k_4 = k_4^0 \exp\left[(1 - \alpha_\mathrm{i} FE/RT - \gamma\theta_2/2)\right] \quad (S15)$$

$$k_{-4} = k_{-4}^0 \exp\left(-\alpha_\mathrm{i} FE/RT + \gamma\theta_2/2\right) \quad (S16)$$

We introduce dimensionless reaction rates

$$V_1 = \frac{RTv_1}{F\Gamma\nu} = K_1^0 \left(1-\theta_1-\theta_2-\theta_3\right)^2 - K_{-1}^0\theta_1^2 \tag{S17}$$

$$V_2 = \frac{RTv_2}{F\Gamma\nu} = K_2 \left(1-\theta_1-\theta_2-\theta_3\right) - K_{-2}\theta_1 \tag{S18}$$

$$V_3 = \frac{RTv_3}{F\Gamma\nu} = K_3\theta_1 - K_{-3} \left(1-\theta_1-\theta_2-\theta_3\right) \tag{S19}$$

$$V_4 = \frac{RTv_4}{F\Gamma\nu} = K_4 \left(1-\theta_1-\theta_2-\theta_3\right) - K_{-4}\theta_2 \tag{S20}$$

$$V_5 = \frac{RTv_5}{F\Gamma\nu} = K_5\theta_2 - K_{-5}\theta_3 \tag{S21}$$

where $\nu = dE/dt$ is the sweep rate and

$$K_1^0 = \frac{RT\Gamma k_1^0 c_{\mathrm{H_2}}}{F\nu} \tag{S22}$$

$$K_{-1}^0 = \frac{RT\Gamma k_{-1}^0}{F\nu} \tag{S23}$$

$$K_2 = \frac{RTk_2 c_{\mathrm{H_2}} c_{\mathrm{OH^-}}}{F\nu} = K_2^0 \exp\left[\left(1-\alpha_2\right)\Delta\right] \tag{S24}$$

$$K_{-2} = \frac{RTk_{-2}}{F\nu} = K_{-2}^0 \exp\left(-\alpha_2\Delta\right) \tag{S25}$$

$$K_3 = \frac{RTk_3 c_{\mathrm{OH^-}}}{F\nu} = K_3^0 \exp\left[\left(1-\alpha_3\right)\Delta\right] \tag{S26}$$

$$K_{-3} = \frac{RTk_{-3}}{F\nu} = K_{-3}^0 \exp\left(-\alpha_3\Delta\right) \tag{S27}$$

$$K_4 = \frac{RTk_4 c_{\mathrm{OH^-}}}{F\nu} = K_4^0 \exp\left[\left(1-\alpha_4\right)\Delta - \left(\gamma/2\right)\theta_2\right] \tag{S28}$$

$$K_{-4} = \frac{RTk_{-4}}{F\nu} = K_{-4}^0 \times \exp\left[-\alpha_4\Delta + \left(\gamma/2\right)\theta_2\right] \tag{S29}$$

$$K_5 = \frac{RTk_5 c_{\mathrm{OH^-}}}{F\nu} = K_5^0 \exp\left[\left(1-\alpha_5\right)\Delta\right] \tag{S30}$$

$$K_{-5} = \frac{RTk_{-5}}{F\nu} = K_{-5}^0 \exp\left(-\alpha_5\Delta\right) \tag{S31}$$

with $\Delta = FE/RT$.

If reaction (S7) replaces reaction (S4) then V_3 is replaced by

$$V_3' = \frac{RTv_5}{F\Gamma\nu} = K_3'\theta_1\theta_2 - K_{-3}' \left(1-\theta_1-\theta_2-\theta_3\right)^2 \tag{S32}$$

in which the rate constants K'_3 and K'_{-3} are now independent of the potential and appropriately redefined ($K_3 = RT\Gamma k_3/F\nu$ and $K_3 = RT\Gamma k_{-3}/F\nu$).

Note that at the equilibrium potential for the hydrogen evolution and oxidation reaction the rates of reactions (S2) through (S4) implies that $V_1 = 0$, $V_2 = 0$, and $V_3 = 0$. Dividing Eq. (S18) by Eq. (S19) and Eq. (S18) by the square root of Eq. (S17) and rearranging gives

$$\frac{K^0_{-3}}{K^0_3} \exp\left(-\Delta_{\mathrm{n,H_2O/H_2}}\right) = \frac{K^0_2}{K^0_{-2}} \exp\left(\Delta_{\mathrm{n,H_2O/H_2}}\right) = \sqrt{\frac{K^0_1}{K^0_{-1}}} \tag{S33}$$

where $\Delta_{\mathrm{n,H_2O/H_2}}$ is the dimensionless null potential $FE_{\mathrm{n,H_2O/H_2}}/RT$ for the hydrogen reaction, and which we take to be equal to zero (RHE scale).

If Eq. (S32) applies, division of this equation with Eqs. (S20) gives

$$\frac{K_3 V'_3}{K^0_{-1}} \frac{\theta_2}{\theta_1} = \frac{K_{-3} V'_3}{K^0_1} \tag{S34}$$

Division of Eq. (S20) by Eq. (S18) gives

$$\frac{K_{-4}}{K_{-2}} \frac{\theta_2}{\theta_1} = \frac{K_4}{K_2} \tag{S35}$$

Substituting Eq. (S35), written as

$$\frac{\theta_2}{\theta_1} = \frac{K^0_{-2} K^0_4}{K^0_2 K^0_{-4}}, \tag{S36}$$

in Eq. (S34) gives

$$\frac{K^0_{-2} K^0_4}{K^0_2 K^0_{-4}} = \frac{K^0_{-1} K'_{-3}}{K^0_1 K'_3} \tag{S37}$$

Finally, with the last relation in Eq. (S33)

$$\frac{K^0_4}{K^0_{-4}} = \frac{K^0_{-2} K'_{-3}}{K^0_2 K'_3} \exp\left(-2\Delta_{\mathrm{n,H_2O/H_2}}\right) \tag{S38}$$

For the coverage θ_i we write

$$\Gamma\frac{d\theta_1}{dt} = 2v_1 + v_2 - v_3 \tag{S39}$$

$$\Gamma\frac{d\theta_2}{dt} = v_4 - v_5 \tag{S40}$$

$$\Gamma\frac{d\theta_3}{dt} = v_5 \tag{S41}$$

where t is the time, or

$$\frac{d\theta_1}{d\tau} = 2V_1 + V_2 - V_3 \tag{S42}$$

$$\frac{d\theta_2}{d\tau} = V_4 - V_5 \tag{S43}$$

$$\frac{d\theta_3}{d\tau} = V_5 \tag{S44}$$

where we have introduced the dimensionless time

$$\tau = \frac{F\nu}{RT}t \tag{S45}$$

If reaction (S7) replaces reaction (S4) then Eq. (S43) is replaced by

$$\frac{d\theta_2}{d\tau} = V_4 - V_3' - V_5 \tag{S46}$$

and V_3' replaces V_3 in Eq. (S42).

Voltammograms

In the absence of transport limitations the current in hydrogen-purged solution becomes

$$I = AF\left(v_2 + v_3 + v_4 + v_5\right) \tag{S47}$$

or

$$\iota = \frac{RTI}{AF^2\Gamma\nu} = V_2 + V_3 + V_4 + V_5 \tag{S48}$$

where we use Eqs. (S17) through Eq. (S21).

If reaction (S7) replaces (S4) then

$$\iota = \frac{RTI}{AF^2\Gamma\nu} = V_2 + V_4 + V_5 \tag{S49}$$

In argon-purged solutions the currents was simulated as

$$\iota = \frac{RTI}{AF^2\Gamma\nu} = V_4 + V_5 \tag{S50}$$

Approximate solutions

Reaction mechanism through Heyrovsky and Volmer steps

Slow Heyrovsky step, reaction (S3). We seek a closed-form approximation to Eqs. (S42) through (S44), and assume that reaction (S2) is slow and does not contribute significantly to the overall reaction and that reactions (S3) and (S4) proceed at the same rate. In other words, we assume that the HOR is controlled by the rate of reaction (S3) and that reaction (S4) proceeds without delay once reaction (S3) has completed. Finally, we assume that the reaction (S5) is irreversible and that the reverse (cathodic) term in Eq. (S20) can be neglected so that

$$V_4 \approx K_4 \left(1 - \theta_1 - \theta_2 - \theta_3\right) \tag{S51}$$

With these approximations Eq. (S42) gives $d\theta_1/d\tau \approx 0$, and since V_5 cancels when adding Eqs. (S43) and (S44) we obtain

$$\frac{d\left(1 - \theta_1 - \theta_2 - \theta_3\right)}{d\tau} = \frac{d\left(1 - \theta_1 - \theta_2 - \theta_3\right)}{d\Delta} \approx -K_4 \left(1 - \theta_1 - \theta_2 - \theta_3\right) \tag{S52}$$

Integration of Eq. (S52) gives

$$
\begin{aligned}
1-\theta_1-\theta_2-\theta_3 &= (1-\theta_1-\theta_2-\theta_3)^* \\
&\times \exp\left(\frac{K_4^0}{1-\alpha}\left\{-\exp\left[(1-\alpha)\Delta\right]+\exp\left[(1-\alpha)\Delta_\mathrm{i}\right]\right\}\right) \quad \text{(S53)}
\end{aligned}
$$

assuming that $1-\theta_1-\theta_2-\theta_3$ has some specific value $(1-\theta_1-\theta_2-\theta_3)^*$ at the initial potential Δ_i and where θ_1^* is the (steady state) value of θ_1. In line with the assumptions above we neglect the last term, V_5, in Eq. (S48). Then

$$
\begin{aligned}
\iota &\approx V_2+V_3+V_4 \approx 2V_2+V_4 \\
&\approx 2K_2\left(1-\theta_1-\theta_2-\theta_3\right)-2K_{-2}\theta_1+K_4\left(1-\theta_1-\theta_2-\theta_3\right) \quad \text{(S54)}
\end{aligned}
$$

Inserting Eq. (S53) into Eq. (S54) gives

$$
\begin{aligned}
\iota &\approx (1-\theta_1-\theta_2-\theta_3)^*\left\{\left(2K_2^0+K_4^0\right)\exp\left[(1-\alpha)\Delta\right]\right\} \\
&\times \exp\left(-\frac{K_4^0}{1-\alpha}\left\{\exp\left[(1-\alpha)\Delta\right]-\exp\left[(1-\alpha)\Delta_\mathrm{i}\right]\right\}\right) \\
&-2K_{-2}^0\exp\left(-\alpha\Delta\right)\theta_1^* \quad \text{(S55)}
\end{aligned}
$$

where we have also assumed that $\alpha_2 \approx \alpha_3 \approx \alpha_4 = \alpha$ and in which we consider θ_1 in the last term constant.

The contributions to the current according to Eq. (S55) are shown in Figure S26. The total current is in qualitative agreement with the experimental curves, increasing initially due to the increasing rates of hydrogen oxidation as the potential increases with some contribution from the oxidation of the nickel itself. As the latter process saturates the surface with adsorbed hydroxyl species the rate of the HOR decreases since the rate-determining step Eq. (S3) depend on the availability of free catalyst surface.

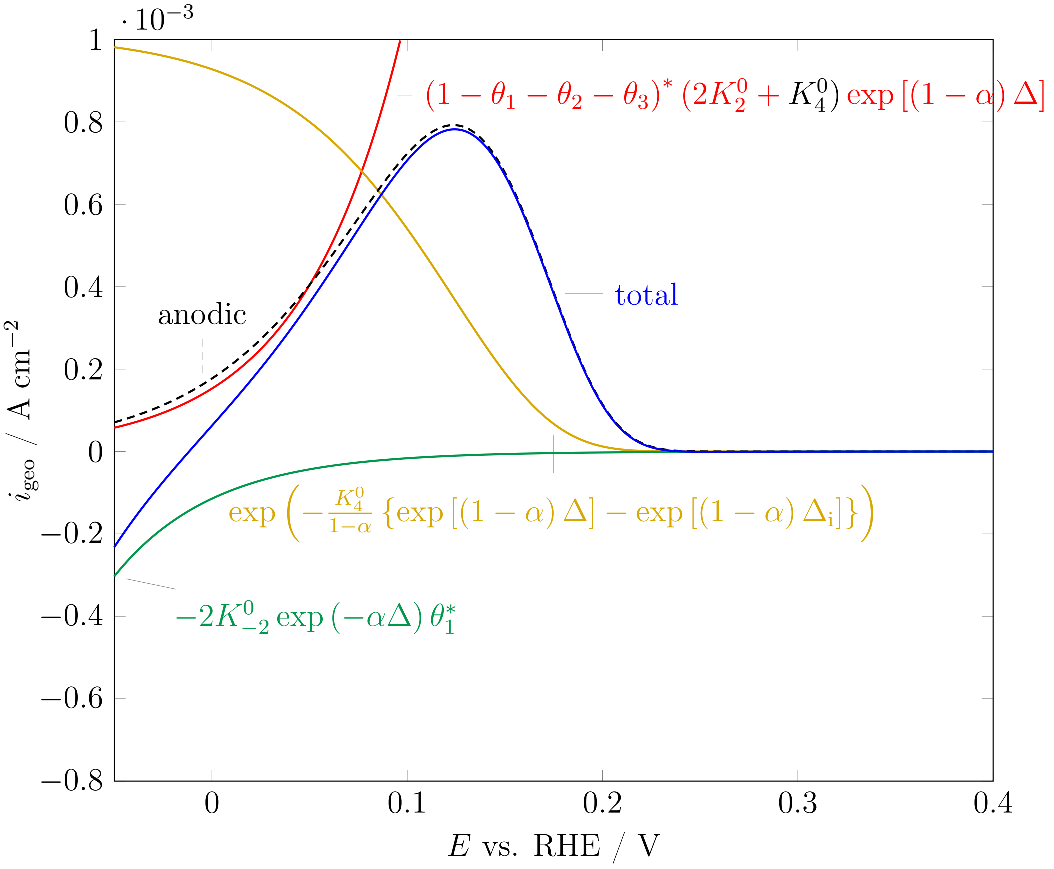

Figure S26: Approximate solution Eq. (S55) for the HOR if its rate is controlled by the Heyrovsky step reaction (S3) and the Tafel step proceeds at a negligible rate. The various contributions to the overall reaction rate are given as indicated in the figure. The red curve corresponds to the anodic part of the hydrogen reaction and the green curve to its cathodic term as they would appear on an electrode with no formation of surface hydroxides. The term $\exp\left(-\frac{K_4^0}{1-\alpha}\left\{\exp\left[(1-\alpha)\,\Delta\right] - \exp\left[(1-\alpha)\,\Delta_\text{i}\right]\right\}\right)$ represents the diminishing available area for the HOR due to reaction (S5).

Slow Volmer step, reaction (S4). If reaction (S4) is slow, the assumption that $d\theta_1/d\tau \approx 0$ may not hold. Subtracting the sum of Eqs. (S42) through (S44) from one we may write

$$\frac{d\left(1-\theta_1-\theta_2-\theta_3\right)}{d\tau} \approx -V_2+V_3-V_4 \tag{S56}$$

in which we have again neglected V_1. If we assume that rate constants K_2 and K_{-2} are large in the sense that

$$\theta_1 \approx \frac{K_2}{K_{-2}}\left(1-\theta_1-\theta_2-\theta_3\right) \tag{S57}$$

V_2 can be computed from Eq. (S42) by use of Eq. (S57) for θ_1,

$$V_2 \approx \frac{d\theta_1}{d\tau}+V_3 \approx \frac{K_2}{K_{-2}}\left(1-\theta_1-\theta_2-\theta_3\right)+\frac{K_2}{K_{-2}}\frac{d\left(1-\theta_1-\theta_2-\theta_3\right)}{d\tau}+V_3 \tag{S58}$$

still neglecting V_1. Inserting Eq. (S57) into Eq. (S19)

$$V_3 \approx \left(K_3\frac{K_2}{K_{-2}}-K_{-3}\right)\left(1-\theta_1-\theta_2-\theta_3\right) \tag{S59}$$

Substituting Eqs. (S51) and (S58) into Eq. (S56) gives

$$\frac{d\left(1-\theta_1-\theta_2-\theta_3\right)}{d\tau} \approx \left(-\frac{K_2/K_{-2}}{1+K_2/K_{-2}} \quad -\frac{K_4}{1+K_2/K_{-2}}\right)\left(1-\theta_1-\theta_2-\theta_3\right) \tag{S60}$$

We set with $x=\left(K_2^0/K_{-2}^0\right)\exp\Delta$ and assume that $\alpha=1/2$ and write Eq. (S60) as

$$\frac{d\left(1-\theta_1-\theta_2-\theta_3\right)}{dx} \approx \left[-\frac{1}{1+x}-K_4^0\sqrt{\frac{K_{-2}^0}{K_2^0}}\frac{x^{-1/2}}{1+x}\right]\left(1-\theta_1-\theta_2-\theta_3\right) \tag{S61}$$

where we have used the definitions of the rate constants, Eqs. (S24), (S25) and (S28).

Integration gives

$$1-\theta_1-\theta_2-\theta_3 \approx (1-\theta_1-\theta_2-\theta_3)^* \exp\left[-\ln\left(\frac{1+x}{1+x_{\mathrm{i}}}\right)\right.$$
$$\left.-2K_4^0\sqrt{\frac{K_{-2}^0}{K_2^0}}\left(\arctan\sqrt{x}-\arctan\sqrt{x_{\mathrm{i}}}\right)\right] \quad \text{(S62)}$$

As before the current is approximated by [Eq. (S54)]

$$\iota \approx V_2 + V_3 + V_4 \quad \text{(S63)}$$

Inserting Eqs. (S51), (S58), and (S59) into Eq. (S63), substituting Eq. (S61) for

$$d\left(1-\theta_1-\theta_2-\theta_3\right)/d\tau = xd\left(1-\theta_1-\theta_2-\theta_3\right)/dx$$

and Eq. (S62) for all factors $(1-\theta_1-\theta_2-\theta_3)$ then gives a closed-form solution for the current ι.

Also in this case the current is the product of a function exponential in Δ multiplied by a modulating function that decreases with increasing potential, Eq. (S62). However, the modulating function is decreasing too slowly to quench the current. This can be seen by considering the limit of large values Δ and equivalently large values of x. Since $\arctan\sqrt{x}$ will approach $\pi/2$ for large x and $1+x \approx x$, $1-\theta_1-\theta_2-\theta_3$ [Eq. (S62)] approaches a constant multiplied with $1/x$. Eq. (S59) for V_3 may therefore be written

$$V_3 \propto K_3 x\frac{1}{x} = K_3^0 \exp\Delta/2 \quad \text{(S64)}$$

for large x. Eq. (S64) therefore predicts that the rate V_3 will increase indefinitely with potential at large potentials, as shown by direct simulation.

A similar consideration for Eq. (S61) gives

$$\frac{d\left(1-\theta_1-\theta_2-\theta_3\right)}{dx} \propto \left[-\frac{1}{x^2} - K_4^0 \sqrt{\frac{K_{-2}^0}{K_2^0}} \frac{1}{x^{5/2}}\right] \tag{S65}$$

in which we have set $1-\theta_1-\theta_2-\theta_3 \propto 1/x$ as above. Combining the latter relation and Eq. (S65) with Eq. (S58) gives $V_2 \approx V_3$ at large Δ and the total current is $\iota \approx 2V_3$, which is then exponential in Δ by Eq. (S64).

The physical explanation for this behaviour is that the as an adsorbed hydrogen atom gets oxidised by reaction (S3) it is immediately filled with another from reaction (S3), the reactions then proceeding at the same rate.

Bifunctional mechanism

Slow Heyrovsky step, reaction (S3). If we assume that reaction (S7) dominates over (S4), then Eqs. (S42) through (S44) has to be replaced by

$$\frac{d\theta_2}{d\tau} = V_4 - V_3' - V_5 \tag{S66}$$

We neglect V_1 and the cathodic term in Eq. (S20) and also eliminate V_5 as before (i.e. adding Eqs. (S43) and (S44)). If also $V_2 \approx V_3'$ then $d\theta_1/d\tau \approx 0$ as before. We assume that reaction (S3) is the step that controls the rate of the HOR and obtain

$$\frac{d\left(1-\theta_1-\theta_2-\theta_3\right)}{d\tau} \approx -K_4\left(1-\theta_1-\theta_2-\theta_3\right) + K_2\left(1-\theta_1-\theta_2-\theta_3\right) - K_{-2}\theta_1 \tag{S67}$$

Neglecting the last term and solving we get

$$1-\theta_1-\theta_2-\theta_3 = \left(1-\theta_1-\theta_2-\theta_3\right)^* \times \exp\left(\frac{K_4^0+K_2^0}{1-\alpha}\left\{-\exp\left[\left(1-\alpha\right)\Delta\right] + \exp\left[\left(1-\alpha\right)\Delta_{\mathrm{i}}\right]\right\}\right) \tag{S68}$$

Therfore, if the HOR is controlled by the rate of reaction (S3), then

$$\iota \approx (1-\theta_1-\theta_2-\theta_3)^* \left\{\left(K_2^0+K_4^0\right)\exp\left[(1-\alpha)\Delta\right]\right\} \times \exp\left(-\frac{K_4^0+K_2^0}{1-\alpha}\left\{\exp\left[(1-\alpha)\Delta\right]-\exp\left[(1-\alpha)\Delta_\mathrm{i}\right]\right\}\right) - 2K_{-2}^0\exp(-\alpha\Delta)\,\theta_1^* \quad \text{(S69)}$$

The voltammogram predicted by Eq. (S69) will be similar to that of Eq. (S55), the mathematical form of Eq. (S69) being the same as that of Eq. (S55).

Parallel Heyrovsky-Volmer and bifunctional mechanism with slow steps (S4) **and** (S7). We now consider the situation in which the Heyrovsky step Eq. (S3) and also reaction (S5) (adsorption of OH) are fast, and reactions (S4) and (S7) are slow but both proceed in parallel. As before we will neglect reaction (S2). Then

$$\theta_2 \approx \frac{K_4}{K_{-4}}(1-\theta_1-\theta_2-\theta_3) \quad \text{(S70)}$$

Eqs. (S42) and (S43) are modified to

$$\frac{d\theta_1}{d\tau} \approx V_2 - V_3 - V_3' \quad \text{(S71)}$$

$$\frac{d\theta_2}{d\tau} = V_4 - V_3' - V_5 \quad \text{(S72)}$$

whereas Eq. (S44) remains as is. Then with Eq. (S70)

$$V_4 = \frac{K_4}{K_{-4}}(1-\theta_1-\theta_2-\theta_3) + \frac{K_4}{K_{-4}}\frac{d(1-\theta_1-\theta_2-\theta_3)}{d\tau} + V_3' + V_5, \quad \text{(S73)}$$

From Eq. (S57)

$$\theta_1 \approx \frac{K_2}{K_{-2}}(1-\theta_1-\theta_2-\theta_3)$$

and Eq. (S71) we obtain

$$V_2 \approx \frac{d\theta_1}{d\tau} + V_3 + V_3' \\ \approx \frac{K_2}{K_{-2}}(1-\theta_1-\theta_2-\theta_3) + \frac{K_2}{K_{-2}}\frac{d(1-\theta_1-\theta_2-\theta_3)}{d\tau} + V_3 + V_3' \quad \text{(S74)}$$

Subtracting the sum of Eqs. (S44), (S71) and (S72) from one gives

$$\frac{d(1-\theta_1-\theta_2-\theta_3)}{d\tau} \approx -V_2 + V_3 + 2V_3' - V_4 \quad \text{(S75)}$$

We insert Eq. (S74) and Eq. (S73) into Eq. (S75) and

$$\left(1+\frac{K_2}{K_{-2}}+\frac{K_4}{K_{-4}}\right)\frac{d(1-\theta_1-\theta_2-\theta_3)}{d\tau} \\ \approx -\left(\frac{K_2}{K_{-2}}+\frac{K_4}{K_{-4}}\right)(1-\theta_1-\theta_2-\theta_3) - V_5 \quad \text{(S76)}$$

We approximate V_5 with the anodic term on the right-hand side of Eq. (S21) so that

$$V_5 \approx K_5\theta_2 = K_5\frac{K_4}{K_{-4}}(1-\theta_1-\theta_2-\theta_3) \quad \text{(S77)}$$

in which we have used Eq. (S70).

Inserting Eqs. (S77) into Eq. (S76) finally gives

$$\left(1+\frac{K_2}{K_{-2}}+\frac{K_4}{K_{-4}}\right)\frac{d(1-\theta_1-\theta_2-\theta_3)}{d\tau} \\ \approx -\left(\frac{K_2}{K_{-2}}+\frac{K_4}{K_{-4}}+K_5\frac{K_4}{K_{-4}}\right)(1-\theta_1-\theta_2-\theta_3) \quad \text{(S78)}$$

We assume $\alpha = 0.5$ and set $x = \left(K_2^0/K_{-2}^0 + K_4^0/K_{-4}^0\right)\exp\Delta$ to obtain

$$\frac{d(1-\theta_1-\theta_2-\theta_3)}{dx} \approx -\left[\frac{1}{1+x}+\frac{Bx^{1/2}}{1+x}\right](1-\theta_1-\theta_2-\theta_3) \quad \text{(S79)}$$

in which

$$B = \frac{K_4^0}{K_{-4}^0} \frac{K_5^0}{\left(K_2^0/K_{-2}^0 + K_4^0/K_{-4}^0\right)^{3/2}}, \tag{S80}$$

The solution to Eq. (S79) is

$$1 - \theta_1 - \theta_2 - \theta_3 \approx (1 - \theta_1 - \theta_2 - \theta_3)^* \left(\frac{1 + x_\mathrm{i}}{1 + x}\right) \times \exp\left\{-2B\left[\left(\sqrt{x} - \arctan\sqrt{x}\right) - \left(\sqrt{x_\mathrm{i}} - \arctan\sqrt{x_\mathrm{i}}\right)\right]\right\} \tag{S81}$$

in which x_i refers to the initial value of x as before.

By Eq. (S49)

$$\iota = V_2 + V_3 + V_4 + V_5$$

in which the rates can be calculated from Eqs. (S32), (S59), (S73), (S74), and (S77). Factors θ_1 and θ_2 can be computed from Eq. (S57) and (S70), respectively. The factors $1-\theta_1-\theta_2-\theta_3$ can be computed from (S81) and its derivative from Eq. (S79).

Fitting models to experimental data

The full (numerical) model was fitted to experimental data through simulated annealing[13] since several downhill methods attempted proved inefficient. The initial temperature was set to 10^4 times the initial value of the objective function (Eq. (S82) below). For each step of the minimisation the differential equations (S42) through (S44) were solved by algorithms for stiff differential equations. The χ^2 objective function consisted of the sum of the difference between experimental current and model current squared,

$$\chi^2 = \sum_i \left\{i_\mathrm{exp}\left(E_i\right) - i_\mathrm{sim}\left(E_i\right)\right\}^2 \tag{S82}$$

where i_exp is the experimental current in A cm^{-2} and i_sim is the simulated current in A cm^{-2}. The parameters emerging from this fit was then used as initial values for a subsequent fit

employing a trust-region-reflective algorithm. In spite of this elaborate procedure, the fits were very sensitive to the initial values from which the final result rarely strayed far. The object functions for parameter sets giving significant differences in the appearance of the simulated voltammograms had very similar values, which is the presumable reason for the difficulty in locating the global minimum.

The minimisation was performed in terms of the logarithm of the parameters K_1^0, K_2^0, K_3^0, K_{-3}^0, K_4^0, K_{-4}^0, K_5^0, K_{-5}^0, γ, Γ, the initial values for the coverage of hydrogen, θ_1^0, and for OH(ad), θ_2^0. A penalty function was employed to ensure a total inital coverage of adsorbed species less than one. The simulated annealing minimisation appeared to be reasonably robust with respect to restarts and temperature, and similar results were obtained independent of these. Several uphill attempts were observed during the minimisation. However, they were to some extent dependent on initial values for the parameters, and the fits do not necessarily represent global minima but should rather be taken as a refined set of parameter values for a given set of initial values. A significant degree of parameter tweaking was therefore necessary in order to obtain reasonable fits.

Fitting of Eq. (S55) and the other analytical approximations was performed with a trust-region-reflective algorithm, but not preceded by simulated annealing. In view of the assumptions behind the analytical model only currents from the forward sweep and up to the current peak were included in the fit. As opposed to the numerical model, fitting to unique parameter sets in this case was straightforward.

Fit results

Figure 27(a) shows an experimental voltammogram in a hydrogen-purged solution for Ni on carbon synthesised by chemical reduction along with the results of fitting the full model to the data as explained above. The simulated voltammogram displays all the features of the experimental voltammogram, including the positive wave on the negative sweep. The forward peak of the simulated voltammogram appears at a potential that is a little too high

for the positive sweep and a little too high on the negative sweep.

The features of the voltammograms such as that in Figure 27(a) may be explained by reference to the model predictions for the reaction rates, which are illustrated in Figure S28. Figure S28 shows the breakdown of the total current in partial currents corresponding to the HOR reaction (steps (S3) and (S4)) and the electrosorption of OH^- to OH_{ad} and $(OH)_2$. It is clear that the forward current is dominated by the HOR steps $V_2 \sim V_3$. The rate of this reaction is, however, throttled by the increasing coverage of OH_{ad} due to reaction (S5), and the two currents reach a maximum at approximately the same potential, beyond which they both decrease due to the exhaustion of available reaction sites. On the return sweep all currents are of a comparable magnitude.

The results are consistent with the Pourbaix-diagram for nickel, Figure S29. The voltammograms in Figure S27 straddle the electrode potential for the formation of Ni hydroxide species from Ni metal.[14] The decrease in the currents beyond approximately 0.22 V in Figure S27 suggests that the hydroxide-covered surface is not catalytically active for the hydrogen-oxidation reaction and that some free Ni surface is required for the oxidation of hydrogen to occur. Therefore, in the return sweep there will be no hydrogen oxidation until part of the nickel surface is reduced back to metallic nickel. If this happens above 0 V on the RHE scale, this will re-start the hydrogen oxidation oxidation reaction. This oxidation current will be superposed on the cathodic current from the reduction of the nickel hydroxide, i.e. reaction (S5) and possibly also reaction (S6) in the cathodic direction (i.e. from right to left). Therefore the *net* current represents a difference and will be smaller than the current in the forward sweep at the same potential; In the forward sweep currents corresponding to reactions (S2) through (S6) all proceed in the anodic direction and therefore all contribute to the total current with the same sign.

The fit quality in the HOR region is not impressive in Figure 27(a), and the model appears to substantially underestimate the kinetics of the HOR. The results for the analytical model based on Eq. (S55), Figure 27(b), are similar to that of the full model for the forward sweep.

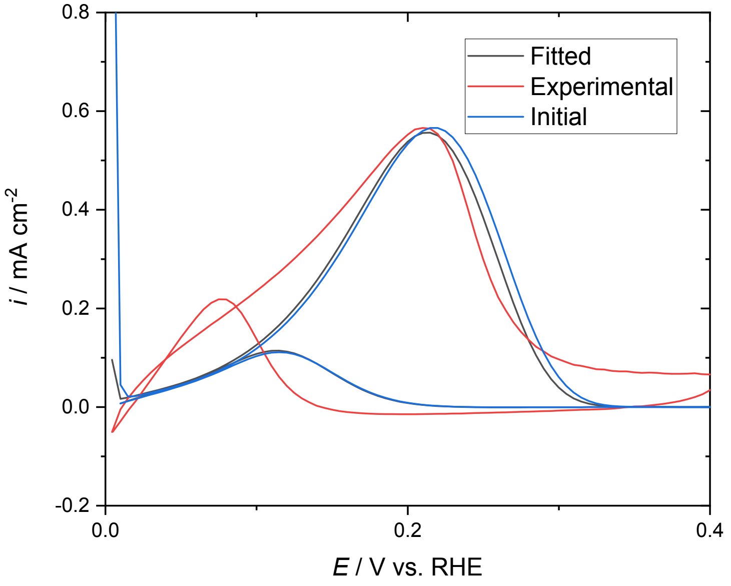

(a)

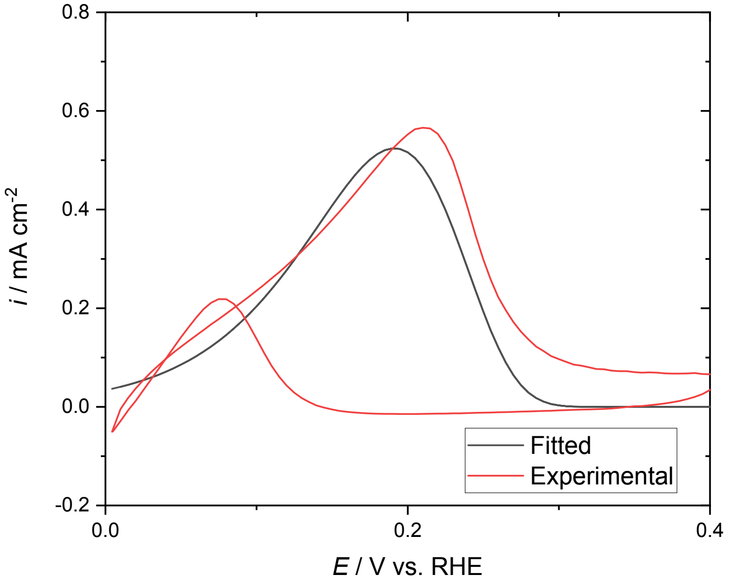

(b)

Figure S27: (a). Experimental and simulated results for sample 2 of Ni/C synthesised by chemical reduction. The simulated results were calculated from Eqs. (S17) through (S48). (b). Experimental and simulated results for sample 2 of Ni/C synthesised by chemical reduction. The simulated results were calculated from Eq. (S55).

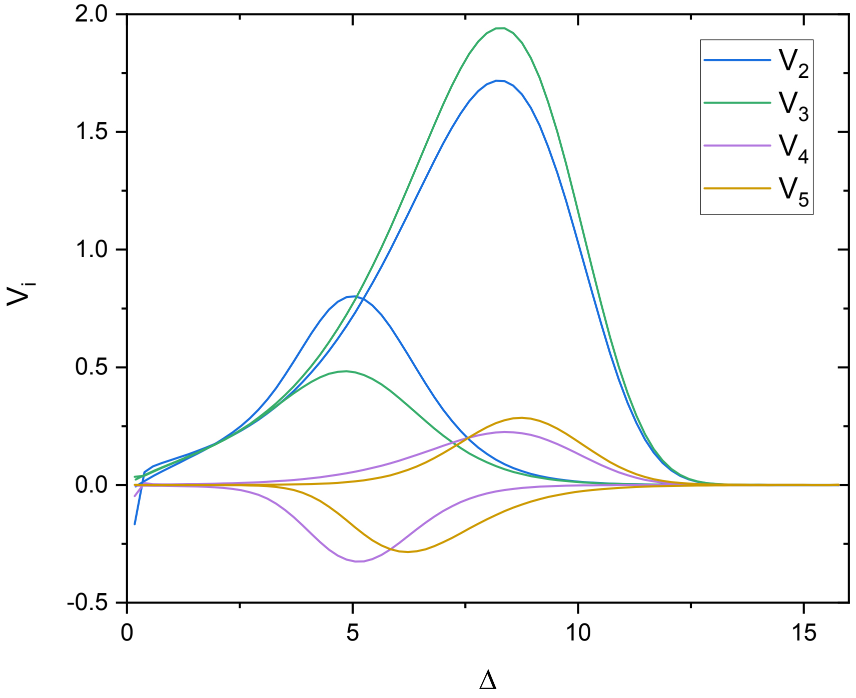

Figure S28: Dimensionless reaction rates with the parameters for the fit in Figure S27.

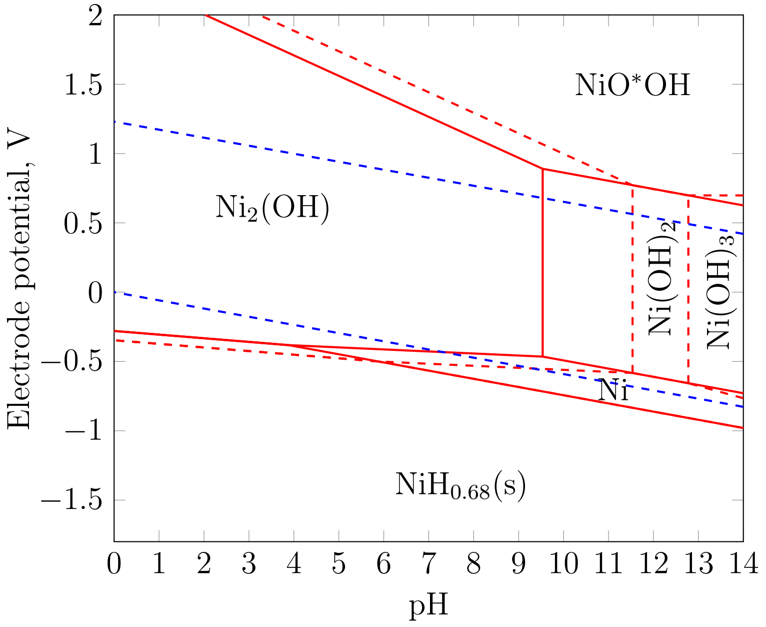

(a)

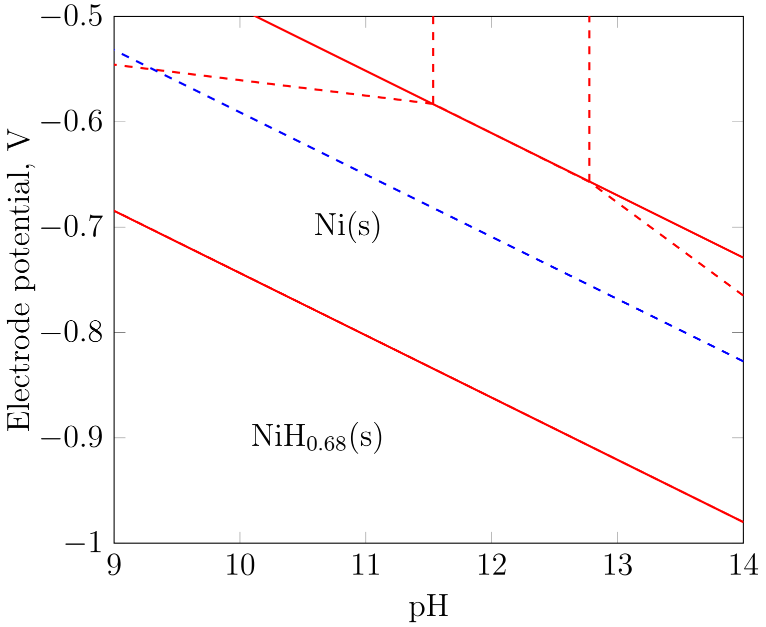

(b)

Figure S29: Pourbaix diagram for Ni. Solid lines represent the diagram for $a_{\mathrm{Ni}} = 1$, and dashed line the diagram for a molality of Ni equal to $m_{\mathrm{Ni}} = 10^{-6}$ mol kg^{-1} H_2O. (a) Complete diagram and (b) expanded view of the hydrogen region at high pH. The blue lines show the electrode potentials for the hydrogen and oxygen reactions. The diagrams were calculated by the EpH package in the HSC Chemistry 9 program, Ver. 9.2.3 by Outotec (`http://www.outotec.com/`).

(The data included in the fit to Eq. (S55) were only those up to the maximum current in the forward sweep, so that the predictions beyond this potential are an extrapolation of the model.) The fitted value for parameter K_4^0 is very different for the two fits, however.

The difference in the fitted values for K_4^0 indicates a significant influence of the part of the voltammogram not included on its value in the full fit. In order to simulate the maximum in current on the negative-going sweep a certain magnitude for K_{-4}^0 is required; otherwise if K_{-4}^0 is too small there would be no release of OH(ad) in the negative-going sweep. Hence there would be no current corresponding to the oxidation of hydrogen in the negative-going sweep since the surface would be fully covered by either OH(ad) or $Ni(OH)_2$ and no bare Ni would be available for catalysing the HOR. Therefore, since the reversible potential for the reaction (S5) and hence (in part) also the potential of the positive-going sweep is determined by the ratio K_4^0/K_{-4}^0, fitting the potential of the forward peak implies a (relatively) large value for K_4^0 also. Thus, the analytical model will underestimate K_4^0 substantially by not including the negative-going sweep.

However, the factor

$$\exp\left(-\frac{K_4^0}{1-\alpha}\left\{\exp\left[(1-\alpha)\,\Delta\right]-\exp\left[(1-\alpha)\,\Delta_{\mathrm{i}}\right]\right\}\right)$$

in Eq. (S55) appears to be an efficient phenomenological representation of the effects of closing the Ni surface for HOR through adsorption of OH(ad), unless one is specifically interested in the values of the rate constants for the adsorption of OH^-.

A fit to data for a Ni sample synthesised by solvothermal reduction is given in Figure S30, both for the full model, Figure 30(a), and to Eq. (S55), Figure 30(b). In this case both the analytical and full models both give reasonable fits in the low-potential region, which is presumably owing to these measurements being extended also to lower potentials. In all other respects the fit display the same features as for the sample in Figure S27. The discrepancy between the parameter K_4^0 was still substantial. However, the pre-exponential

factor $(1-\theta_1-\theta_2-\theta_3)^*(2K_2^0+K_4^0)$ in Eq. (S55) is in a similar magnitude as K_2^0 from the full fit, which is expected since the former would approach K_2^0 if $K_2^0 >> K_4^0$.

Also for the simulated voltammogram in Figure S30 were the reaction rates in reasonable agreement with the assumption that $V_2 \sim V_3$, as shown in Figure S31.

A fit to the same data set as in Figure S30 but with a different initial set of initial parameters is given in Figure S32. The corresonding reaction rates are given in Figure S33.

Figure S34 shows the results of fitting the analytical models to the experimental data. The data included in the fit were only those up to the maximum current in the forward sweep, so that the predictions beyond this potential are an extrapolation of the model. For each current-voltage relation the anodic contribution can be written as a term increasing exponentially with the potential and a modulating function $f(\Delta)$ that decreases with increasing potential. Thus, if the modulating term decreases faster than exponentially the current will display a maximum in potential in the positive-going sweep as in Figure 34(a). If the modulating function decreases less strongly with increasing potential, the current will increase indefinitely as in Figure 34(b). To which extent this happens depend on the relative rates of the reactions. For example if reactions (S3) and (S4) are both fast and reaction (S5) slow, the HOR will outcompete the formation of Ni(OH). We consider this to be unlikely to happen in practice, though, since eventually reaction (S3) is likely to become rate-determining at very high coverages of adsorbed OH, θ_2. The models assuming that the Heyrovsky step, Eq. (S3), is slow, i.e. Eq. (S55), give a reasonably good quantitative agreement with the experimental data. Figure S35 shows the fit of this model to the data for Ni_3Co/C also in this case the fit is, in view of the simplicity of the model, very good.

Evaluating the potential of surface passivation from di/dE

The derivative of the dimensionless current in a hydrogen-saturated solution with respect to potential for the parameters used for the simulated voltammograms in Figures Figure S30 and S31 is given in Figure S36. The figure also contains the rates V_4 and V_5 simulated both in

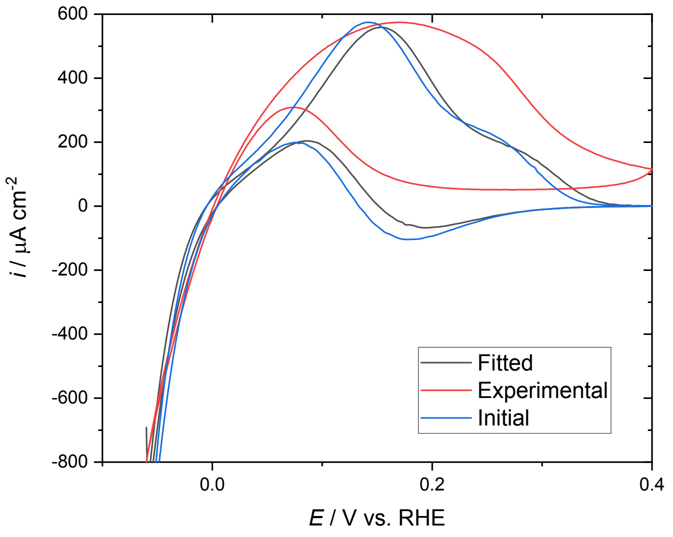

(a)

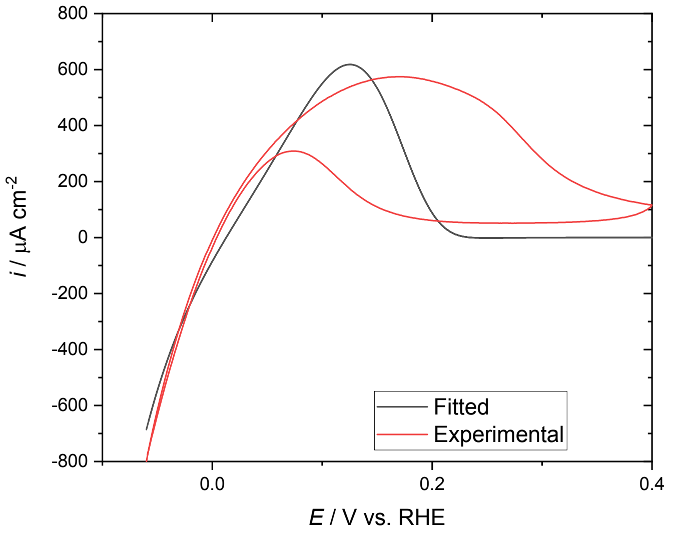

(b)

Figure S30: (a). Experimental and simulated results for sample 5 of Ni/C synthesised by the solvothermal reduction. The simulated results were calculated from Eqs. (S17) through (S48). (b). Experimental and simulated results for sample 5 of Ni/C synthesised by solvothremal reduction. The simulated results were calculated from Eq. (S55).

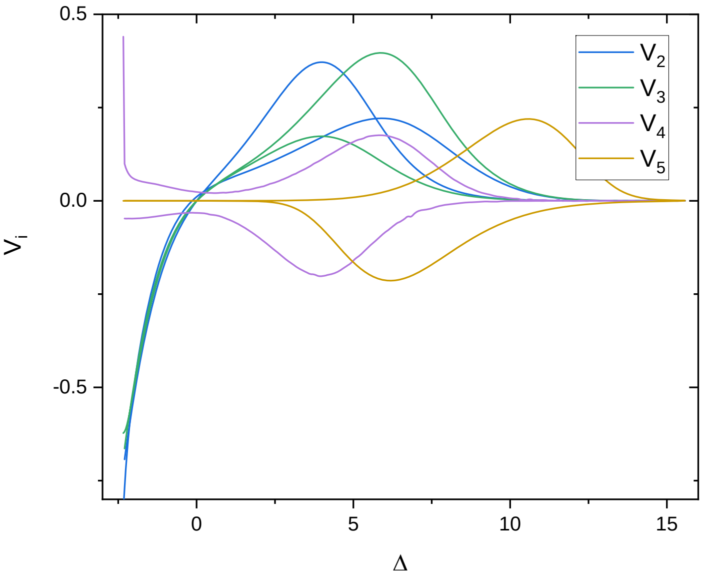

Figure S31: Simulated dimensionless reaction rates for reactions (S3) through (S6) for the voltammogram in Figure S30.

hydrogen-saturated and in argon-purged solutions. The two reactions (S5) and (S6) are more widely separated (in terms of potential) in Ar-containing solutions than for those purged with hydrogen. However, the minimum in the curve for di/dE still appears approximately at the potential corresponding to the potential at which the decrease in the rate of the HOR due to surface passivation by adsorbed OH and $Ni(OH)_2$ and increase in the rate of the HOR due to the increasing potential balance. Therefore, beyond this potential the rate of the HOR drops.

References

(1) Wei, R.; Fang, M.; Dong, G.; Ho, J. C. *Science Bulletin* **2017**, *62*, 971–973.

(2) Chen, R.; Yang, C.; Cai, W.; Wang, H.-Y.; Miao, J.; Zhang, L.; Chen, S.; Liu, B. *ACS Energy Letters* **2017**, *2*, 1070–1075.

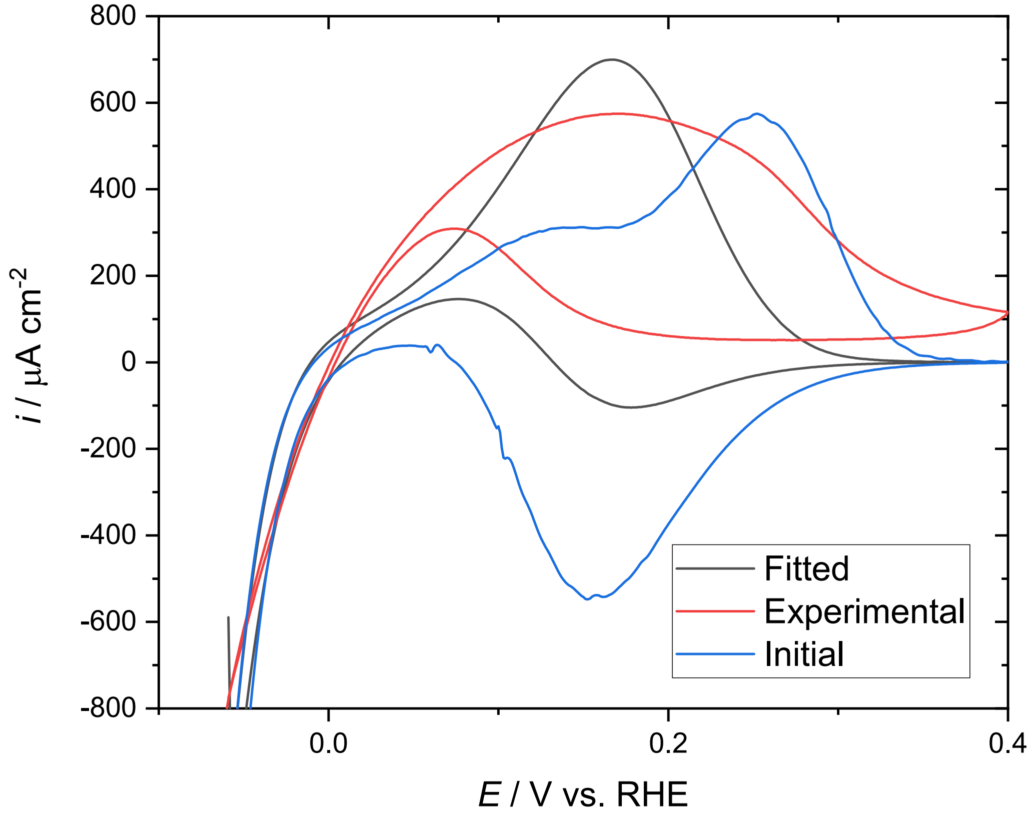

Figure S32: Experimental and simulated results for sample 5 of Ni/C synthesised by the solvothermal reduction but with a different set of initial parameters than in Figure S30. The simulated results were calculated from Eqs. (S17) through (S48)..

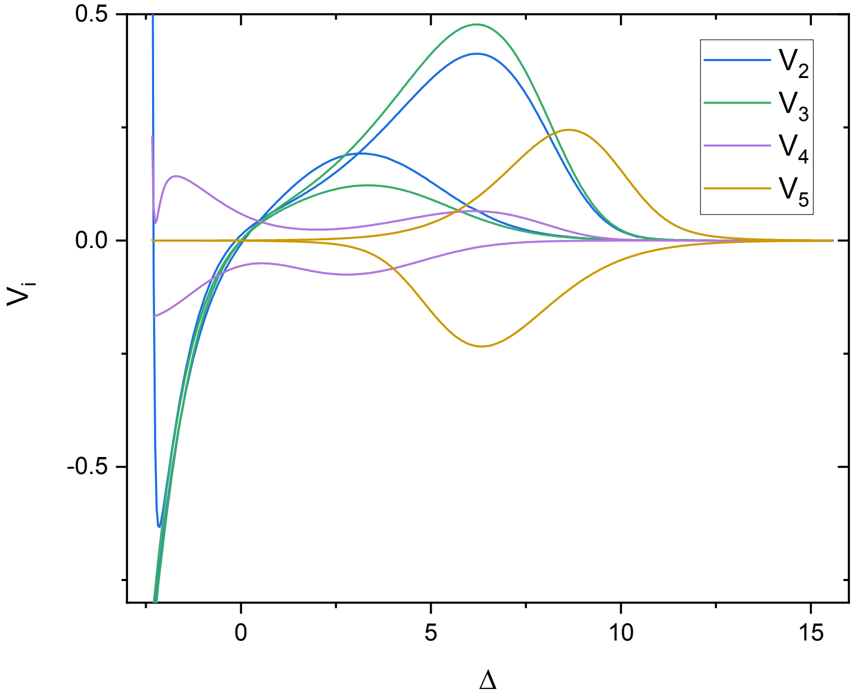

Figure S33: Dimensionless reaction rates with the parameters for the fit in Figure S32.

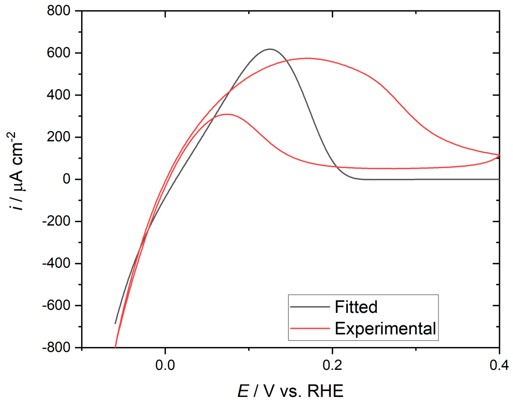

(a) Heyrovsky-Volmer mechanism, Eq. (S3) *rds*

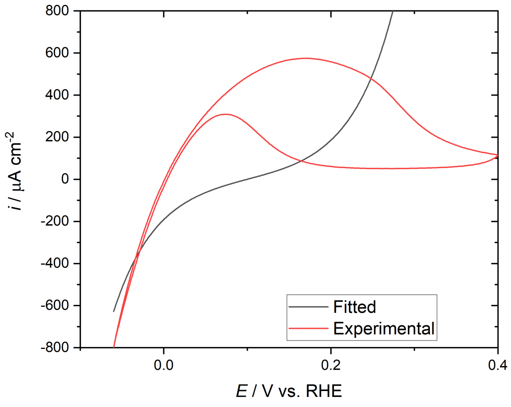

(b) Heyrovsky-Volmer mechanism, Eq. (S4) *rds*

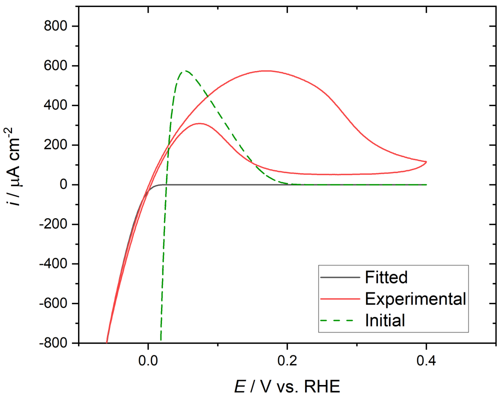

(c) Bifunctional mechanism, Eq. (S7) *rds*, approximation B

Figure S34: Experimental and simulated results for Ni/C synthesised by solvothermal reduction. The simulated results were calculated from the analytical, approximate solutions to the microkinetic model for the reactions in Eqs. (S2) through (S6). (a) Heyrovsky-Volmer mechanism, Eq. (S3) *rds*. (b) Heyrovsky-Volmer mechanism, Eq. (S4) *rds*. (c) Bifunctional mechanism, Eq. (S7) *rds*.

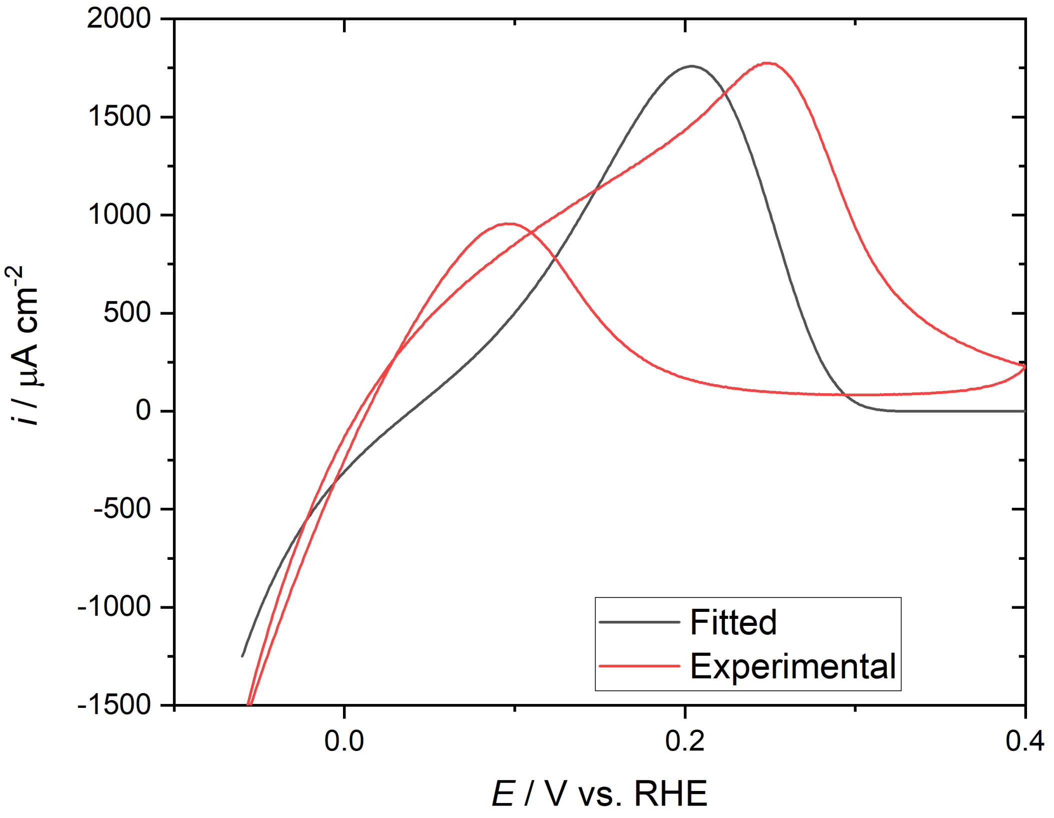

Figure S35: Experimental and simulated res ults for Ni_3Co/C synthesised by solvothermal reduction. The simulated results were calculated from the analytical, approximate solution to the microkinetic model for the Heyrovsky-Volmer mechanism, Eq. (S3) *rds*, Eqs. (S2) through (S6) assuming Eq. (S3) to represent the slow step.

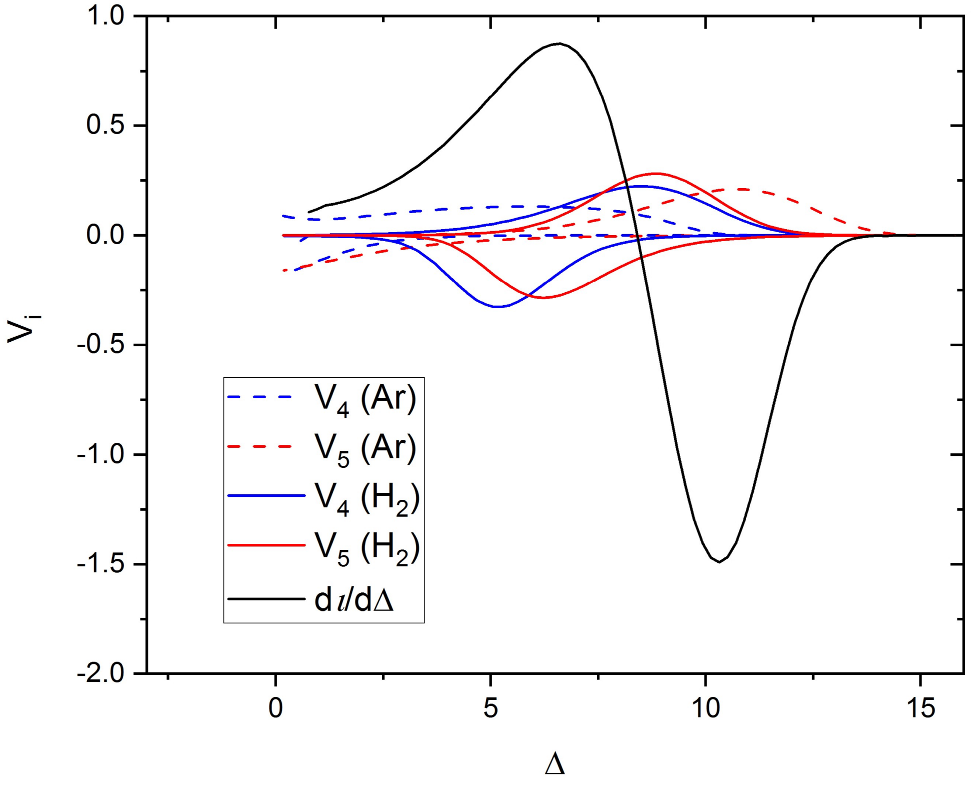

Figure S36: Simulated dimensionless reaction rates for reactions (S5) (V_4) and (S6) (V_5) in Ar- and H_2-purged solutions vs. dimensionless potential simulated with parameters corresponding to samples synthesised by chemical reduction. The red and blue solid lines are the rates in hydrogen-containing solutions, and the dashed lines are the rates in argon-purged solutions. The solid black line is the simulated curve for the derivative of the dimensionless current with respect to the dimensionless potential, $d\iota/d\Delta$, corresponding to di/dE.

(3) Rodriguez, P.; Tichelaar, F. D.; Koper, M. T. M.; Yanson, A. I. *Journal of the American Chemical Society* **2011**, *133*, 17626–17629.

(4) Oshchepkov, A. G.; Bonnefont, A.; Saveleva, V. A.; Papaefthimiou, V.; Zafeiratos, S.; Pronkin, S. N.; Parmon, V. N.; Savinova, E. R. *Topics in Catalysis* **2016**, *59*, 1319–1331.

(5) Kabir, S.; Lemire, K.; Artyushkova, K.; Roy, A.; Odgaard, M.; Schlueter, D.; Oshchepkov, A.; Bonnefont, A.; Savinova, E.; Sabarirajan, D. C.; Mandal, P.; Crumlin, E. J.; Zenyuk, I. V.; Atanassov, P.; Serov, A. *J. Mat. Chem. A* **2017**, *5*, 24433–24443.

(6) Oshchepkov, A. G.; Bonnefont, A.; Pronkin, S. N.; Cherstiouk, O. V.; Ulhaq-Bouillet, C.; Papaefthimiou, V.; Parmon, V. N.; Savinova, E. R. *J. Power Sources* **2018**, *402*, 447–452.

(7) Durst, J.; Siebel, A.; Simon, C.; Hasche, F.; Herranz, J.; Gasteiger, H. A. *Energy & Environmental Science* **2014**, *7*, 2255–2260.

(8) Jia, Q.; Liu, E.; Jiao, L.; Li, J.; Mukerjee, S. *Current Opinion in Electrochemistry* **2018**, *12*, 209–217.

(9) Sheng, W.; Zhuang, Z.; Gao, M.; Zheng, J.; Chen, J. G.; Yan, Y. *Nature Communications* **2015**, *6*, 5848.

(10) Strmcnik, D.; Uchimura, M.; Wang, C.; Subbaraman, R.; Danilovic, N.; van der Vliet, D.; Paulikas, A. P.; Stamenkovic, V. R.; Markovic, N. M. *Nature Chemistry* **2013**, *5*, 300–306.

(11) Salmazo, D.; Juarez, M. F.; Oshchepkov, A. G.; Cherstiouk, O., V; Bonnefont, A.; Shermukhamedov, S. A.; Nazmutdinov, R. R.; Schmickler, W.; Savinova, E. R. *Electrochimica Acta* **2019**, *305*, 452–458.

(12) Press, W. H.; Teukolsky, S.; Vetterling, W. T.; Flannery, B. P. *Numerical Recipes in Fortran*; Cambridge University Press, Cambridge, 1994.

(13) Ref. 12, p. 436 – 438.

(14) Alsabet, M.; Grden, M.; Jerkiewicz, G. *Electrocatal.* **2011**, *2*, 317–330.
